# From Coordination to Noncoordination: Syntheses and Substitution Lability Studies of Titanium Triflato Complexes

**DOI:** 10.1021/acs.inorgchem.4c01033

**Published:** 2024-07-26

**Authors:** Kevin Schwitalla, Zainab Yusufzadeh, Marc Schmidtmann, Rüdiger Beckhaus

**Affiliations:** Institut für Chemie, Carl von Ossietzky Universität Oldenburg, Oldenburg D-26111, Federal Republic of Germany

## Abstract

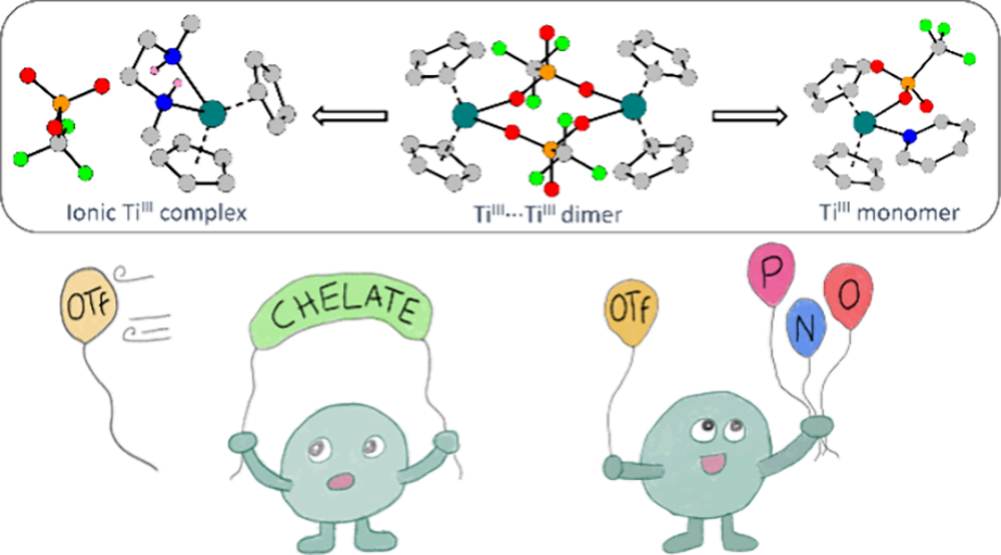

A new concept for obtaining cationic complexes with triflate counteranions from coordinating triflato ligands was developed. Various routes are leading to titanium(IV) and titanium(III) triflato complexes efficiently. The reactions of pentafulvene titanium complexes with either triflic acid or silver triflate give the corresponding titanium(IV) triflato complexes in excellent yields. Hydrolysis of the titanium(IV) bistriflato complexes leads to cationic aqua complexes via displacement of the triflato ligand, which consequently acts as a noncoordinating anion. A functionalized titanium(IV) monotriflato complex was synthesized by insertion of a nitrile into the Ti–C bond and the triflato ligand was displaced by an NHC. While the titanium(IV) complexes are mostly inert toward substrates, the donor-free titanium(III) triflato complex is a strong Lewis acid and forms various adducts with monodentate Lewis bases. The titanium(III) complex was oxidized by reaction with TEMPO, resulting in a diamagnetic titanium(IV) complex. The reaction with bidentate ligands results in cationic titanium(III) complexes due to displacement of the triflato ligand by the bidentate ligands. Treatment with acetone leads to an aldol reaction of two acetone molecules and the formation of a cationic diacetone alcohol complex.

## Abstract 2

The synthesis of titanium triflate complexes and substitution reactions leading to the formation of ionic complexes are described. While the Ti(IV) complexes only react with strong donors such as water or NHCs, the Ti(III) triflate complex is a strong Lewis acid and forms various adducts with monodentate Lewis bases. Reaction with acetone induces an aldol reaction, forming diacetone alcohol. The use of bidentate ligands causes displacement of the triflato ligand into a triflate anion, thus forming ionic complexes.

## Introduction

**Scheme 1:**
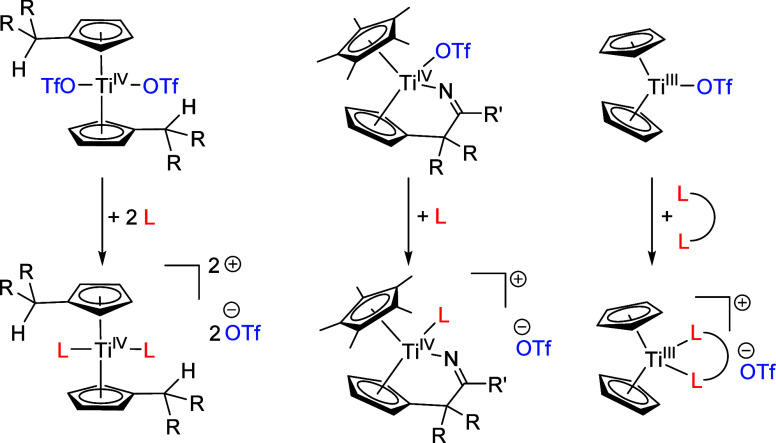
This Work’s Concept of Triflato Ligand Displacement by Other Ligands

Early transition metal triflato (or trifluoromethanesulfonato, CF_3_SO_3_^–^, TfO^–^) complexes play an important role in homogeneous catalysis. They are involved in the catalytic Mukaiyama aldol,^1,2^ Sakurai–Hosomi,^3,4^ Diels–Alder^5−7^ and Friedel–Crafts reactions,^8−10^ which are powerful C–C coupling reactions. One of the earliest and most simple Ti(IV) triflato complexes was the titanocene(IV) triflato complex, which was synthesized and described via X-ray diffraction as early as 1980 by Thewalt et al.^11^ The synthesis was further improved and expanded by Luinstra using the respective metallocene dimethyl complex and triflic acid.^12^ Despite its early synthesis, this complex is of recent interest in catalytic studies of radical arylation reactions and tetrahydrofuran synthesis and an improved, silver-free synthesis route was developed.^13^ Since the first synthesis of the titanocene(IV)triflato complex, various chiral titanocene-based complexes have been used to perform catalytic, enantioselective reactions.^14−16^ Although there are many examples of Ti(IV) triflato complexes, the number of Ti(III) triflato complexes is very limited.^17,18^ One of the rare examples, namely Cp*_2_Ti(OTf), was synthesized by oxidation of a Cp*_2_Ti(acetylene) complex with Fe(OTf)_3_ and was used to study water splitting reactions.^19^ Another Ti(III) triflato complex (Cp_2_Ti(OTf)(THF)) was described earlier by Berhalter and Thewalt,^20^ however, this complex was not further studied and is to date the only example of a Cp_2_Ti(III) triflato complex. Regarding the reactivity of early transition metal triflato complexes, there are numerous examples of salt-metathesis reactions that substitute the triflate anion entirely.^21−23^ Less attention has been paid to the displacement of the triflato ligand out of the coordination sphere of the metal, despite being seen as a weakly coordinating anion in the past.^24^ The triflate anion is currently regarded as a moderately coordinating anion^24−26^ and thus, studies of early transition metal complexes with triflates as noncoordinating anions are rather limited.^19,27−32^ In this work, we developed facile and efficient routes toward Ti(IV) and Ti(III) triflato complexes. We studied their reactivity toward a series of Lewis bases and demonstrated the substitution lability of the coordinating triflato ligands by displacing them with water, NHC and bidentate ligands (Scheme 1).

## Results and Discussion

### Titanium(IV) Bistriflato Complexes

**Scheme 2:**
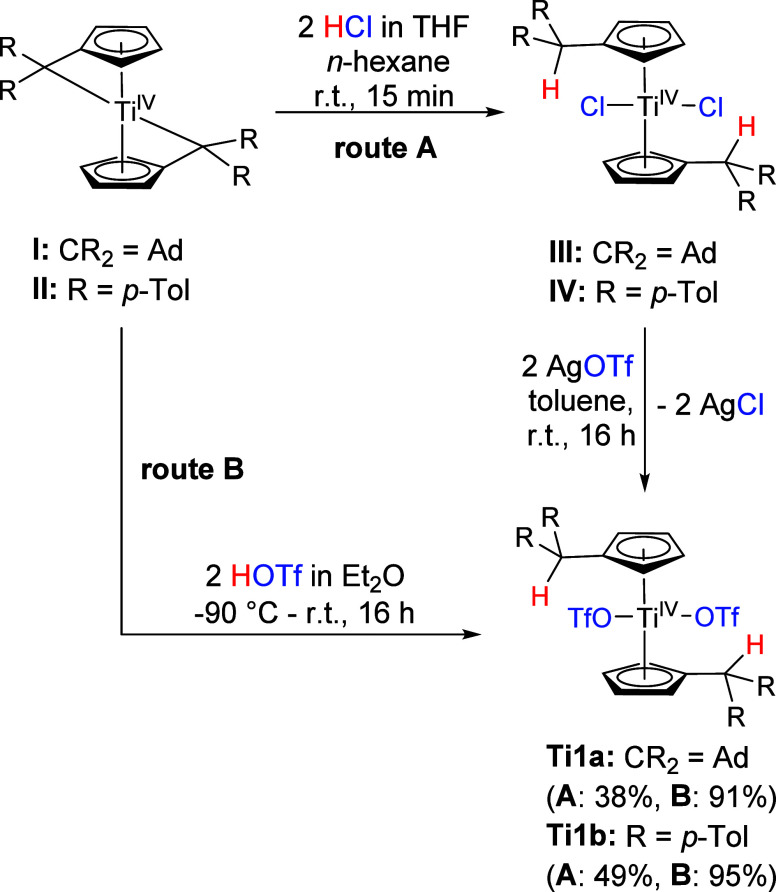
Reaction of **I** and **II** with HCl in THF to Intermediates **III** and **IV** and Consecutive Reaction with AgOTf to form **Ti1a,b** (Route **A**); Direct Reaction of **I** and **II** with HOTf in Et_2_O to form **Ti1a,b** (Route **B**)

The Ti(IV) triflato complexes **Ti1a,b** are accessible via routes **A** and **B** (Scheme 2) using the bis(π–η^5^:σ–η^1^-pentafulvene)titanium complexes **I** and **II** as starting materials. Because their chemistry is characterized by a broad range of E—H activation reactions,^33^ they are excellent starting materials for obtaining triflato complexes. Route **A** is a two-step reaction, starting with the protonolysis of the pentafulvene ligands by HCl to yield the dichloride complexes **III** and **IV** as intermediates. Consecutive salt metathesis with silver triflate (AgOTf)^34^ forms the triflato complexes **Ti1a,b**. In route **B**, the bis(π–η^5^:σ–η^1^-pentafulvene)titanium complexes **I** and **II** react directly with triflic acid (HOTf), diluted in ether, to form **Ti1a,b** via protonolysis by HOTf. In comparison, route **B** is clearly preferred as it is a one-step reaction without coproducts and superior isolated yields, but it is important to comply with the reaction conditions as the concentration of the HOTf and the right temperature are crucial to the outcome of these reactions.

**Figure 1:**
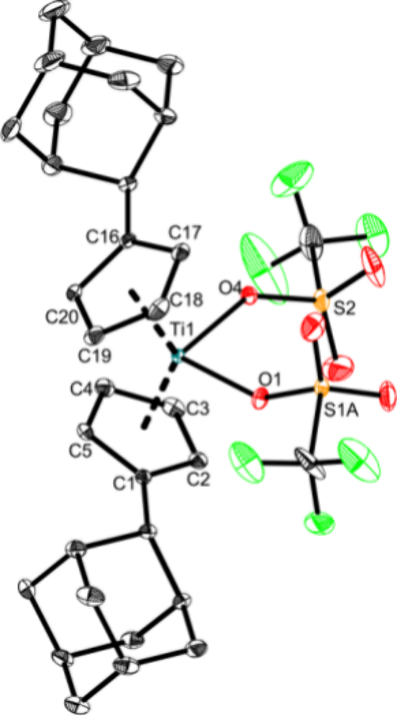
Molecular structure of complex **Ti1a**. Displacement ellipsoids are drawn at the 50% probability level. H atoms and solvent molecules have been omitted for clarity. Selected bond lengths (Å) and angles (deg): Ti1—O1 2.0285(12), Ti1—O4 2.0385(12), S1A—O1 1.4651(14), S2—O4 1.4804(12), O1—Ti1—O4 88.65(5), Ct1—Ti1—Ct2 131.6 (Ct1 = centroid of C1–C5; Ct2 = centroid of C16–C20).

Both products were confirmed via NMR spectroscopy and in case of **Ti1a**, also by single crystal X-ray diffraction (Figure 1). The ^1^H NMR spectra of **Ti1a** and **Ti1b** show the Cp-H signals as two multiplets with integrals of 4 and the C_exo_H protons as singlets of 2H, which demonstrates the high symmetry of the complexes (SI, Figures S1 and S3). The characteristic CF_3_ groups of **Ti1a** and **Ti1b** can be found as a quartet in the ^13^C NMR spectra at 120.1 and 120.0 ppm respectively (^1^*J*_CF_= 318 Hz each) and correlate to a coordinating triflato ligand.^35^ The ^19^F chemical shift (−76.8 ppm, −76.4 ppm) are in accordance with other coordinating triflato ligands but are less characteristic to determine the coordination of the triflato ligand.^35^

The molecular structure of **Ti1a** reveals two coordinating triflato ligands with slightly elongated Ti–O single bonds (2.0285(12) Å and 2.0385(12) Å) when compared to the sum of covalent radii (Σr_cov_(Ti–O) = 1.99 Å),^36^ which are in accordance with the Ti–O bonds (2,000(7) Å, 2,003(7) Å) of Cp_2_Ti(OTf)_2_.^11^

**Scheme 3:**
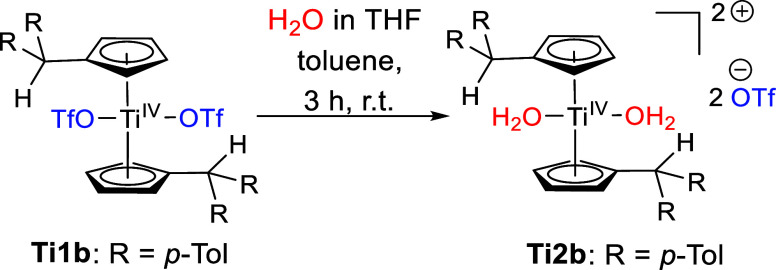
Reaction of **Ti1b** with H_2_O in THF to Form **Ti2b**

Due to the interesting interactions of triflato complexes with water (water splitting of Cp*_2_TiOTf),^19^ we studied the reactivity of **Ti1a**,**b** toward water. The stoichiometric reaction of **Ti1b** with H_2_O, diluted in THF, resulted in the formation of the dicationic diaqua complex **Ti2b** by substitution of the two triflato ligands with two H_2_O ligands (Scheme 3).

**Figure 2:**
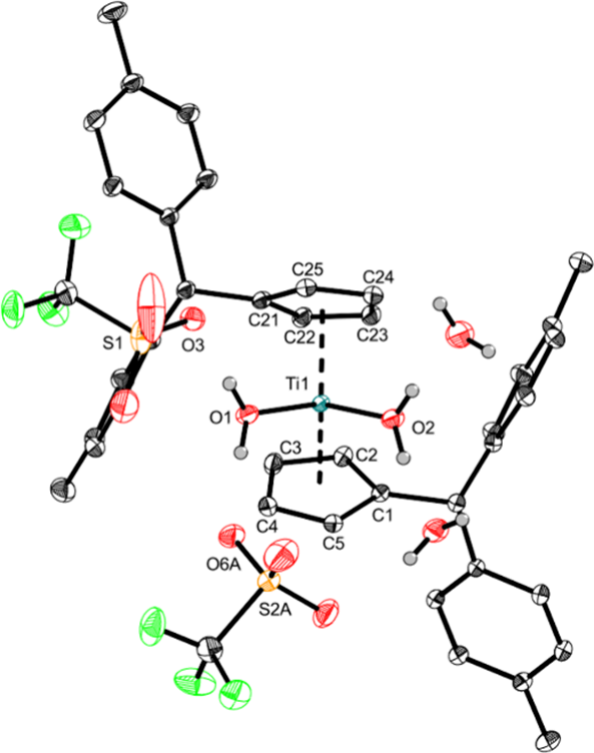
Crystal structure of complex **Ti2b**. Displacement ellipsoids are drawn at the 50% probability level. H atoms (apart from H_2_O H atoms) and solvent molecules (apart from two adjoining H_2_O units) have been omitted for clarity. Selected bond lengths (Å) and angles (deg): Ti1—O1 2.0517(7), Ti1—O2 1.9990(7), O1—Ti1—O2 91.15(3), Ct1—Ti1—Ct2 131.8 (Ct1 = centroid of C1–C5; Ct2 = centroid of C21–C25).

The displacement of the triflato ligands by water is indicated by NMR experiments. A broad signal at 8.88 ppm in the ^1^H NMR spectrum with 4 H is assigned to the protons of the aqua ligands and the signal at −78.9 ppm in the ^19^F NMR spectrum is shifted by approximately −2.1 ppm. Despite our best efforts, the CF_3_ group could not be detected in the ^13^C NMR spectrum. The stoichiometric reaction of **Ti1a** with H_2_O only resulted in a mixture of products. No suitable single crystals could be obtained directly for **Ti2b** either. However, by adding of an excess of H_2_O to saturated solutions of **Ti1a** and **Ti1b** in C_6_D_6_, suitable crystals of **Ti2a** (SI, Figure S15) and **Ti2b** (Figure 2) were obtained.

The crystal structure of **Ti2b** revealed a significant difference between the two Ti–O bonds (2.0517(7) Å and 1.9990(7) Å) of the water ligands. This is due to the different hydrogen bonding. The hydrogen atoms of O1 form hydrogen bonds to the triflate anions (H···O = 1.791(19) Å and 1.82(2) Å), whereas the hydrogen atoms of O2 form hydrogen bonds to cocrystallizing water molecules (1.752(19) Å and 1.78(2) Å), which can be described as moderately strong hydrogen bonds.^37^ In other examples [Cp_2_Ti(H_2_O)_2_](CF_3_SO_3_)_2_),^32,38^ the Ti–O bonds (2.004(3) Å, 2.035(3) Å; 2.062(6) Å, 2.089(6) Å) are more similar forming more equal hydrogen bonds. We further performed reactivity studies of **Ti1a**,**b** with a broad range of substrates (amines, pyridines, alcohols, nitriles, phosphine oxides NHC). However, we could not detect any reactivity. The substitution reactions of **Ti1a,b** with 2,2-bipyridine were also investigated, but the reactions resulted in mixtures of products. The products were identified as the 2,2-bipyridine adducts **Ti1a,bipy** and **Ti1b,bipy** with Cp groups substituted instead of the triflato ligands and redox reactions occurred to Ti(III) (SI, Figures S16 and S17).

### Titanium(IV) Monotriflato Complexes

**Scheme 4:**
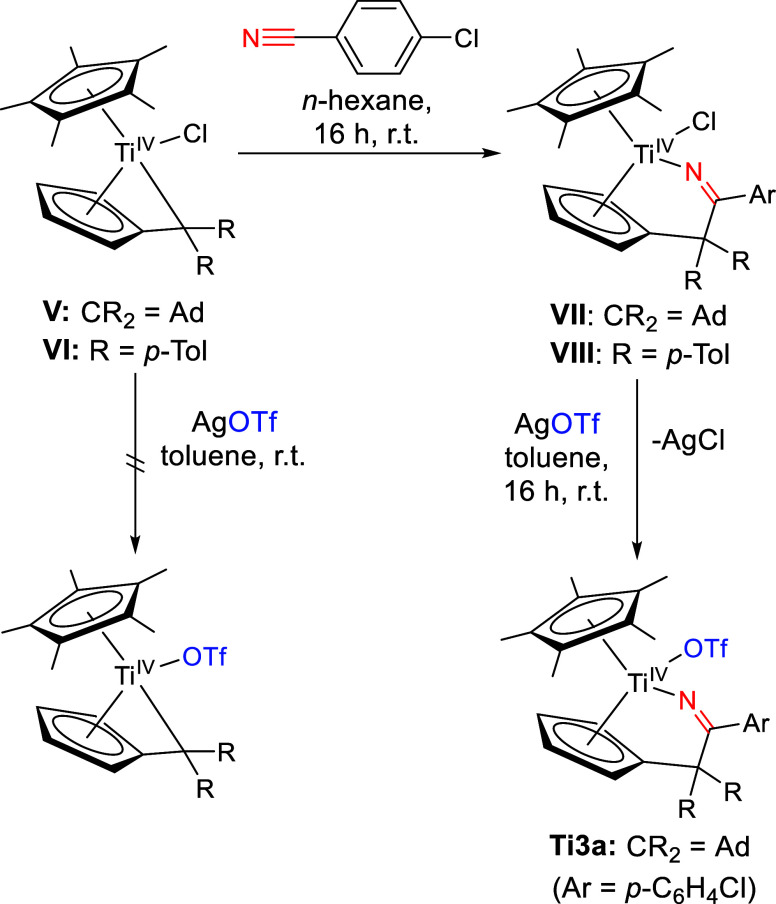
Reaction of **V** and **VI** with 4-Chlorobenzonitrile to Intermediates **VII** and **VIII** and Consecutive Reaction of **VII** with AgOTf to Form **Ti3a** Direct reactions of **V** and **VI** with AgOTf did not form the desired product.

We previously synthesized cationic titanium complexes with multidentate ligand frameworks for FLP reactivity via Ti–C functionalization of mono(pentafulvene)titanium complexes.^39−41^ Because of their successful FLP reactivity, we were also interested in the potential applications of the respective triflato complexes. The direct reaction of Cp*(π–η^5^:σ–η^1^-pentafulvene)titanium(IV) complexes **V** and **VI** with AgOTf did not yield the desired triflato complexes and the reaction only resulted in a mixture of products. Therefore, the rather vulnerable pentafulvene unit was functionalized by forming the insertion products **VII** and **VIII** via reaction with 4-chlorobenzonitrile. This was followed by reaction with AgOTf, substituting the chloride group for triflate, though only **Ti3a** was isolated (Scheme 4).

**Figure 3:**
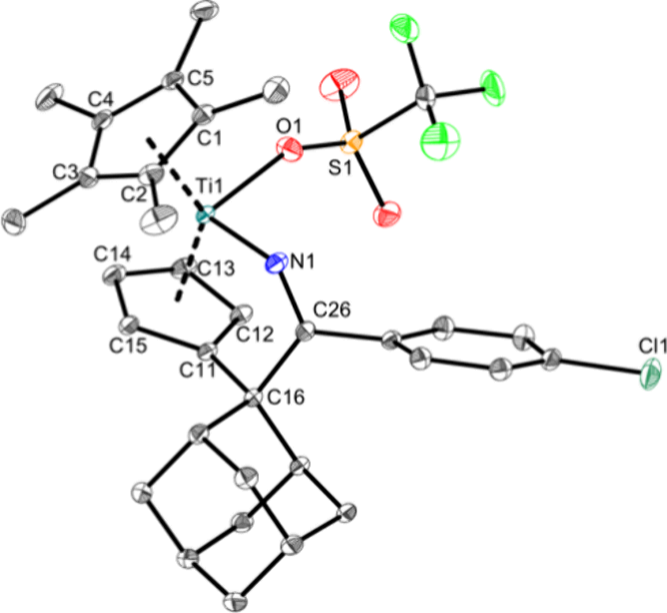
Molecular structure of complex **Ti3a**. Displacement ellipsoids are drawn at the 50% probability level. H atoms and solvent molecules have been omitted for clarity. Selected bond lengths (Å) and angles (deg): Ti1—O1 2.0732(8), Ti1—N1 1.9422(9), N1—C26 1.2712(12), C16—C26 1.5666(14), S1—O1 1.4609(7), O1—Ti1—N1 95.744(40), N1—C26—C27 117.805(83), Ct1—Ti1—Ct2 134.8 (Ct1 = centroid of C1–C5; Ct2 = centroid of C11–C15).

The formation of **Ti3a** was confirmed via NMR spectroscopy and single-crystal X-ray diffraction. The CF_3_ group correlates to a quartet in the ^13^C NMR spectrum at 120.3 ppm (^1^*J*_CF_= 319 Hz), and the ^19^F signal is located at −77.0 ppm, corresponding to a coordinating triflato ligand,^35^ which was also confirmed by the X-ray structure (Figure 3).

The Ti–O bond with 2.0732(8) Å is again slightly elongated compared with the covalent radii (Σr_cov_(Ti–O) = 1.99 Å)^36^ and also slightly longer than in **Ti1a** and Cp_2_Ti(OTf)_2_.^42^ The Ti–N bond with 1.9422(9) Å shows some double bond character (Σr_cov_(Ti–N) = 2.07 Å, Σr_cov_(Ti = N) = 1.77 Å))^43^ due to the attractive Ti(d_π_)–N(p_π_) interactions.^39^

**Scheme 5:**
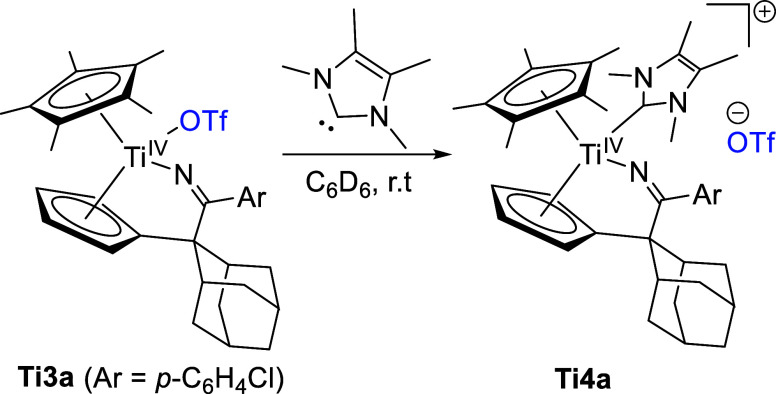
Reaction of **Ti3a** with 1,3,4,5-Tetramethylimidazol-2-ylidene to Form **Ti4a**

The substitution reactions of **Ti3a** with most substrates (water, pyridine, nitriles, phosphine oxides) was unsuccessful and **Ti3a** was reisolated. However, the reaction with 1,3,4,5-tetramethylimidazol-2-ylidene (NHC) led to the cationic NHC complex **Ti4a** (Scheme 5).

**Figure 4:**
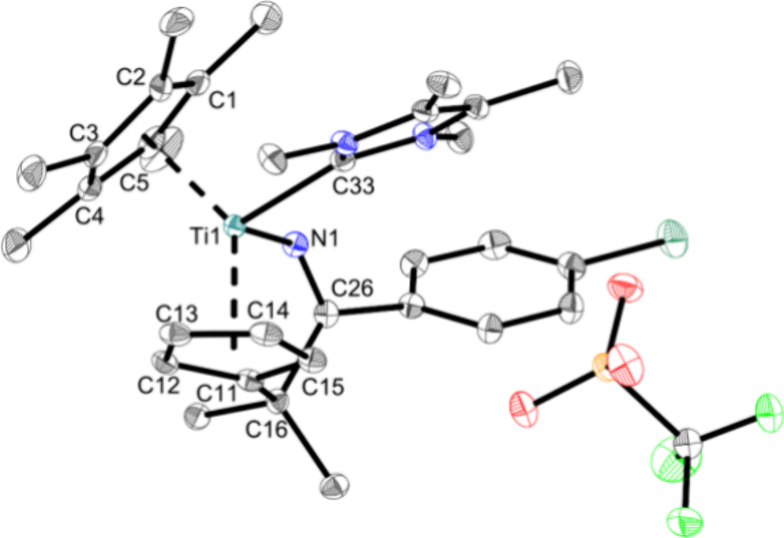
Crystal structure of complex **Ti4a**. Displacement ellipsoids are drawn at the 50% probability level. H atoms and solvent molecules have been omitted for clarity. Selected bond lengths (Å) and angles (deg): Ti1—C33 2.277(3), Ti1—N1 1.940(3), N1—C26 1.267(4), C16—C26 1.564(4), C33—Ti1—N1 100.46(11), Ct1—Ti1—Ct2 136.1 (Ct1 = centroid of C1–C5; Ct2 = centroid of C11–C15).

The ^19^F NMR shift of **Ti4a** (−77.8 ppm) indicates that the triflato ligand was displaced by the NHC because it is slightly shifted by 0.8 ppm from **Ti3a** (−77.0 ppm). Despite our best efforts, the CF_3_ group could not be detected in the ^13^C NMR spectrum. However, the crystal structure of **Ti4a** revealed that the displacement of the triflato ligand occurred (Figure 4).

Similar to **Ti3a**, the Ti–N bond of **Ti4a** can be described as a shortened single bond with 1.940(3) Å, which is comparable to similar complexes.^39^ The substituted triflate anion does not interact with the metal center. Instead, the NHC interacts via the Ti–C33 bond (2.277(3) Å), which correlates to an elongated single bond according to the covalent radii (Σr_cov_(Ti–C) = 2.11 Å)^36^ and is similar to other Ti NHC complexes (Ti(AdFv)_2_(NHC) (2.342(2) Å), Ti(NMe_2_)_2_Cl_2_(NHC) (2.313(5) Å), TiCl_2_(NMe_2_)(NHC)_2_) (2.293(3)).^44−46^ Because of the limitation of substitution reactions we found for these Ti(IV) complexes, we opted for midvalent Ti(III) complexes.

### Titanium(III) Triflato Complexes

**Scheme 6:**
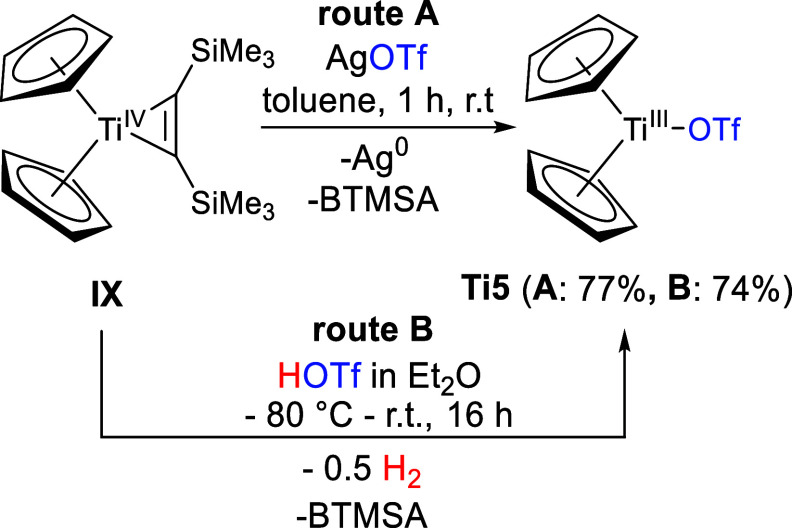
Reaction of **IX** with AgOTf (Route **A**) and HOTf (Route **B**) to Form **Ti5**

For the synthesis of the Ti(III) triflato complex **Ti5**, titanocene bis(trimethylsilyl)acetylene **IX** is either oxidized with AgOTf, yielding Ag^0^ and bis(trimethylsilyl)acetylene (BTMSA) as coproducts (Scheme 6, route **A**) or by reaction with 0.1 M triflicic acid (HOTf) via reduction of the proton to hydrogen and the oxidized Ti(III) triflato complex (Scheme 6, route **B**). This is possible because of the redox reactivity of **IX**.^47,48^

**Figure 5:**
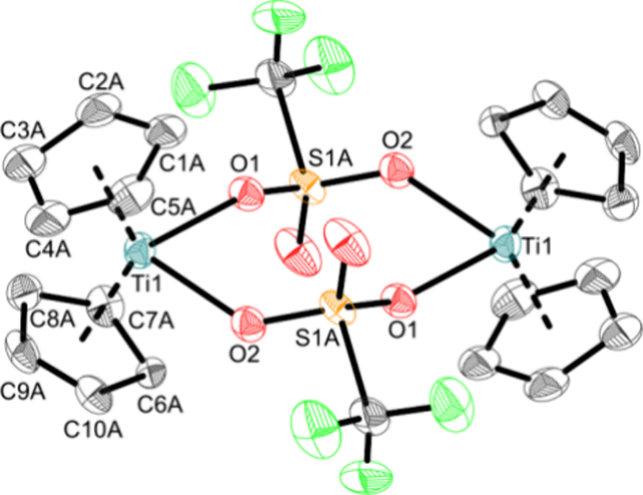
Molecular structure of complex **Ti5**. Displacement ellipsoids are drawn at the 50% probability level. H atoms and solvent molecules have been omitted for clarity. Selected bond lengths (Å) and angles (deg): Ti1—O1 2.1528(19), Ti1—O2 2.180(2), S1A—O1 1.442(2), S1A—O2 1.427(2), O1—Ti1—O2 81.51(7), Ct1—Ti1—Ct2 133.5 (Ct1 = centroid of C1A–C5A; Ct2 = centroid of C6A–C10A).

Due to their paramagnetic nature, the characterization of **Ti5** and its Ti(III) products is not conclusive via NMR spectroscopy as the signals are highly broadened (example **Ti5a**, SI, Figure S12). Therefore, the molecular structures and the purity of the compounds were identified by single-crystal X-ray diffraction, EPR spectroscopy (SI, Figures S22–S30) and elemental analyses. The molecular structure of **Ti5** is shown in Figure 5.

**Scheme 7:**
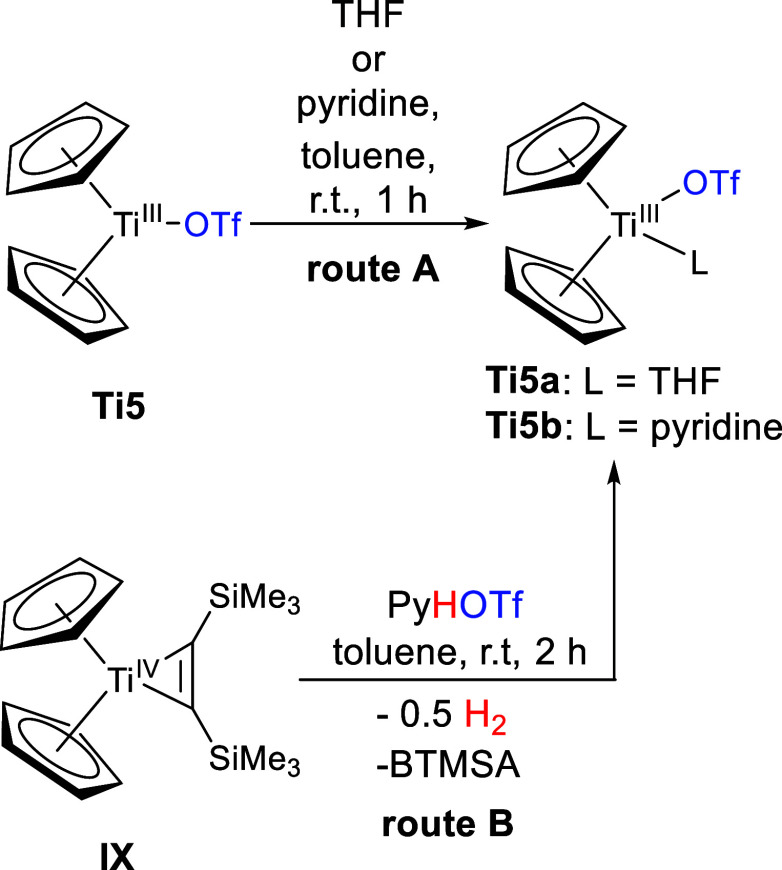
Reaction of **Ti5** with THF or Pyridine to form **Ti5a,b** (Route **A**); Reaction of **IX** with Pyridinium Triflate to Form **Ti5b** (Route **B**)

As revealed by the molecular structure (Figure 5), **Ti5** is a dimeric structure in the solid state with two bridging triflato units. This complex is most likely also dimeric in solution in noncoordinating solvents. Nonetheless for reasons of simplification, the monomer is used in the following schemes. This structural motif is typical for unsaturated metal triflato complexes and can be found in several works.^21,49−52^ The slightly asymmetrical Ti–O bonds (2.1528(19) Å and 2.180(2) Å) are best described as elongated single bonds according to the covalent radii (Σr_cov_(Ti–O) = 1.99 Å) and are similar to other triflato bridged titanium complexes ([Cp*Ti(BH_4_)(μ-O_2_SOCF_3_)_2_]: 2.123(3) Å,^53^ [Cp*TiCp*(CF_3_SO_3_)(μ-O_2_SOCF_3_)_2_)X]_2_: 2.164(3) Å, 2.142(4) Å).^52^ The Ti–O bonds are much longer than those of the monomeric complexes **Ti1a**, **Ti3a**, and Cp_2_Ti(OTf)_2_.^11^ This is due to the resonance of this bridged complex, and the covalent bond of each bridging triflato ligand is shared across the two titanium centers. **Ti5** must be treated under strict anaerobe conditions because it readily reacts with trace amounts of oxygen and/or water to the μ-oxo complex **Ti5oxo** (SI, Figure S18), which is similar to the μ-oxo zirconium analog [ZrCp_2_(CF_3_SO_3_)]_2_O.^54^**Ti5** acts as a Lewis acid and reacts with Lewis bases. By treating **Ti5** with coordinating solvents such as tetrahydrofuran or pyridine, the monomeric Lewis adducts **Ti5a,b** are formed (Scheme 7, route **A**). This can be performed either stoichiometrically or with an excess of the solvent. Alternatively, **Ti5b** is synthesized directly by reaction of **IX** with pyridinium triflate (PyHOTf) via reduction of the proton to hydrogen and exchange of BTMSA with pyridine and the triflato ligand (Scheme 7, route **B**).

**Figure 6:**
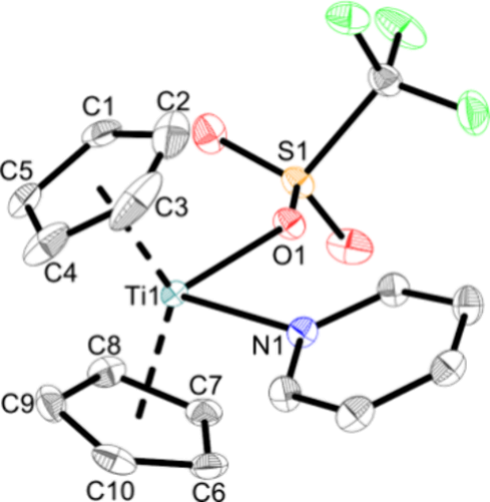
Molecular structure of complex **Ti5b**. Displacement ellipsoids are drawn at the 50% probability level. H atoms and solvent molecules have been omitted for clarity. Selected bond lengths (Å) and angles (deg): Ti1—O1 2.1459(16), Ti1—N1 2.2531(19), S1—O1 1.4747(17), O1—Ti1—N1 83.44(7), Ct1—Ti1—Ct2 135.2 (Ct1 = centroid of C1–C5; Ct2 = centroid of C6–C10).

The molecular structures of **Ti5a,b** were confirmed by single-crystal X-ray diffraction and while the structure of **Ti5b** is new (Figure 6), **Ti5a** was already known and confirmed by the unit cell parameters.^20^

The molecular structure of **Ti5b** shows the monomeric titanocene(III) triflato complex with a coordinating pyridine ligand. The Ti–N bond (2.2531(19) Å) corresponds to a typical Ti–N(pyridine) bond^55^ and is in accordance with other pyridine adducts of titanocene-based Lewis acids (Cp*Ti(NNPh_2_)Cl(py): 2.266(2) Å,^56^ Cp_2_Ti = NTer(py): 2.268(4) Å^57^). The Ti–O bond (2.1459(16) Å) is comparable to **Ti5** and longer than in the Ti(IV) complexes **Ti1a**, **Ti3a** and Cp_2_Ti(OTf)_2_.^11^ This either indicates that the oxidation state might play a role^48^ or that the pyridine affects the bonding of the triflato ligand.

**Scheme 8:**
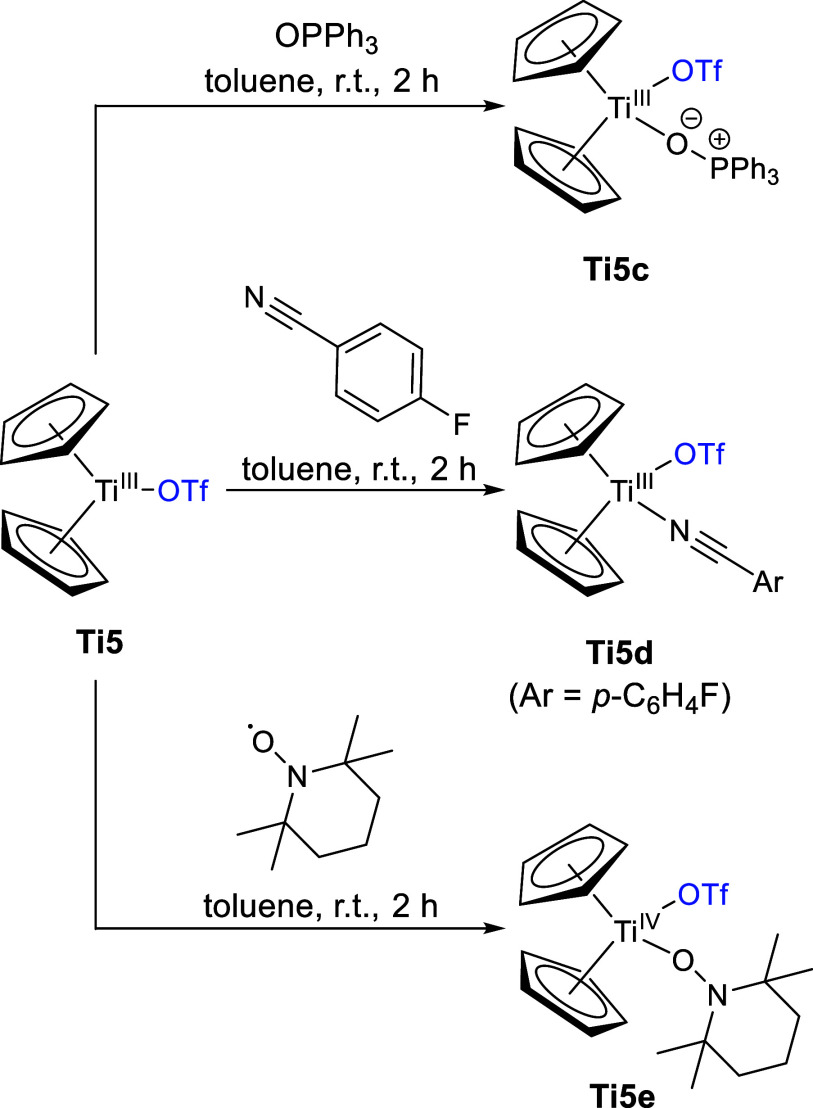
Reaction of **Ti5** with Triphenylphosphine Oxide, 4-Fluorobenzonitrile and TEMPO to form **Ti5c,d,e**

**Figure 7:**
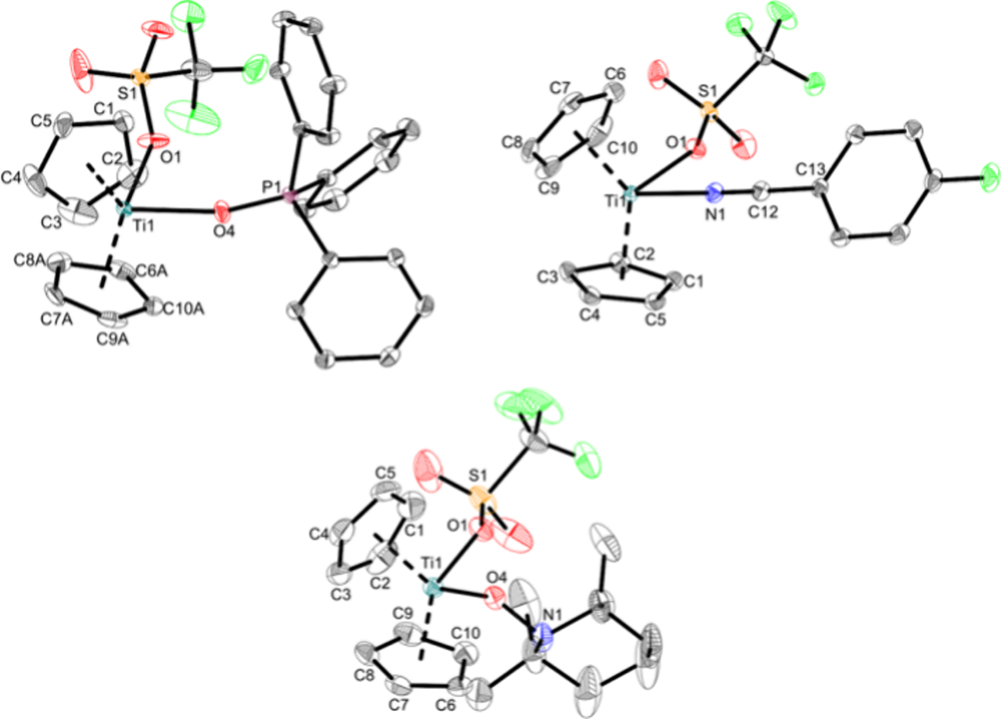
Molecular structures of complexes **Ti5c** (top left), **Ti5d** (top right), and **Ti5e** (bottom). Displacement ellipsoids are drawn at the 50% probability level. H atoms and solvent molecules have been omitted for clarity. Selected bond lengths (Å) and angles (deg): **Ti5c**, Ti1—O1 2.1922(6), Ti1—O4 2.0960(6), S1—O1 1.4562(6), P1—O4 1.4987(6), O1—Ti1—O4 78.83(3), Ti1—O4—P1 164.68(4), Ct1—Ti1—Ct2 132.6; **Ti 5d**, Ti1—O1 2.1527(9), Ti1—N1 2.1602(10), S1—O1 1.4570(9), N1—C12 1.1485(15), C12—C13 1.4305(16), N1—Ti1—O1 77.54(4), Ti1—N1—C12 175.27(10), N1—C12—C13 178.15(13), Ct1—Ti1—Ct2 135.5; **Ti5e**, Ti1—O1 2.0932(8), Ti1—O4 1.8474(10), S1—O1 1.4677(9), N1—O4 1.4346(16), O1—Ti1—O4 94.24(4), Ti1—O4—N1 142.08(11), Ct1—Ti1—Ct2 130.3 (Ct1 = centroid of C1–C5; Ct2 = centroid of C6–C10).

Given the successful reactions with coordinating solvents, we performed further reactions with triphenylphosphine oxide, 4-fluorobenzonitrile, and (2,2,6,6-tetramethylpiperidin-1-yl)oxyl (TEMPO) (Scheme 8). Similar to **Ti5a,b**, the titanocene(III) triflato complex forms monomeric adducts with the respective Lewis base, forming **Ti5c** with triphenylphosphine oxide, **Ti 5d** with 4-fluorobenzonitrile, and **Ti5e** with TEMPO, all characterized by single crystal X-ray diffraction (Figure 7). Because of the oxidizing effect of TEMPO, the resulting complex is a Ti(IV) complex, which was additionally characterized via NMR spectroscopy (SI, Figure S13).

The ^1^H NMR spectrum confirms the addition of one TEMPO ligand to the complex because the relative integrals of the Cp ligands match the set of signals from the TEMPO moiety. The ^19^F signal is found at −77.6 ppm, and the chemical shift of the respective triflato carbon atom in the ^13^C NMR spectrum (120.7 ppm) corresponds to a coordinating triflato ligand.^35^

**Ti5c** displays an even longer Ti–O1 bond (2.1922(6) Å) of the triflato ligand than **Ti5b** (2.1459(16) Å) while the Ti–O4 bond of the phosphine oxide (2.0960(6) Å) corresponds to an elongated single bond (Σr_cov_(Ti–O) = 1.99 Å)^36^ and is slightly longer in comparison with other titanium triphenylphosphine oxide complexes (2.017(6) Å,^58^ 2.0404(11) Å^57^). The Ti–O bond of **Ti 5d** (2.1527(9) Å) is similar to that of **Ti5b** (2.1459(16) Å), indicating that N-based ligands have a similar effect on the Ti–OTf bond. The Ti–N bond (2.1602(10) Å) and the C–N bond of the nitrile (1.1485(15) Å) are similar to that of another Ti-*p*-FBN adduct (Ti–N: 2.1740(14) Å, C–N: 1.143(2) Å).^57^ The Ti1—O1 bond of the triflato ligand of **Ti5e** (2.0932(8) Å) is similar to those of the Ti(IV) complexes **Ti1a**, **Ti3a**, and Cp_2_Ti(OTf)_2_,^11^ while the Ti1—O4 bond (1.8474(10) Å) of the TEMPO ligand is similar to those of other titanium TEMPO complexes (1.8228(16) Å,^29^ 1.824(2) Å^59^).

**Scheme 9:**
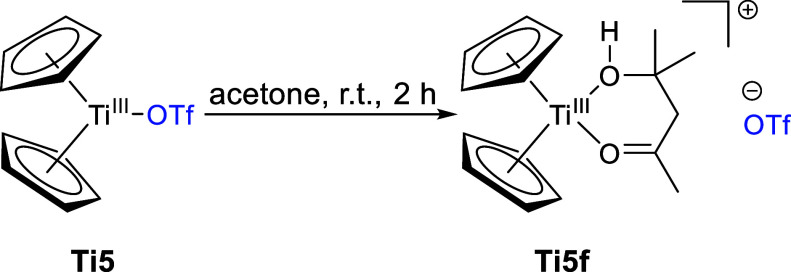
Reaction of **Ti5** with Acetone to form **Ti 5f**

The reaction of **Ti5** with an excess of acetone does not yield the Lewis adduct, instead, we observed the aldol reaction of two acetone units to diacetone alcohol (DAA), as confirmed by single-crystal X-ray diffraction (Scheme 9).

**Figure 8:**
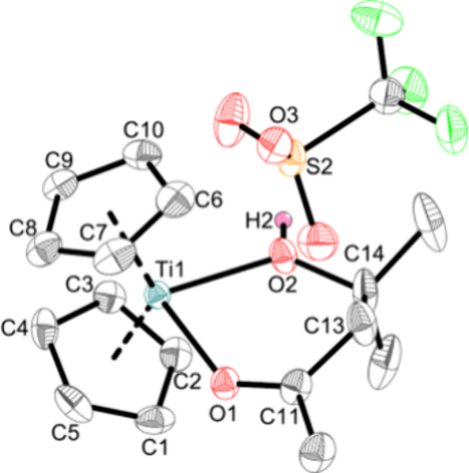
Crystal structure of complex **Ti5f**. Displacement ellipsoids are drawn at the 50% probability level. H atoms and solvent molecules have been omitted for clarity. Selected bond lengths (Å) and angles (deg): Ti1—O1 2.135(2), Ti1—O2 2.1474(18), S2—O3 1.450(2), C11—O1 1.234(3), C14—O2 1.459(3), C11—C13 1.503(4), C11—C13 1.527(4), O3—H2 1.69(4), O1—Ti1—O2 79.73(7), Ct1—Ti1—Ct2 134.1 (Ct1 = centroid of C1–C5; Ct2 = centroid of C6–C10).

The DAA coordinates in bidentate fashion with both oxygen atoms, displacing the triflato ligand, which is coordinated to the hydrogen atom of the alcohol via a hydrogen bond with an H···O bond distance of 1.69(4) Å (Figure 8).

Both Ti–O bonds (2.135(2) Å, 2.1474(18) Å) are elongated single bonds according to the sum of covalent radii (Σr_cov_(Ti–O) = 1.99 Å),^36^ confirming the dative bonding nature of these bonds. It is rare to observe such complexes and only a few complexes reveal a DAA ligand after the aldol reaction of two acetone molecules.^60−62^

**Scheme 10:**
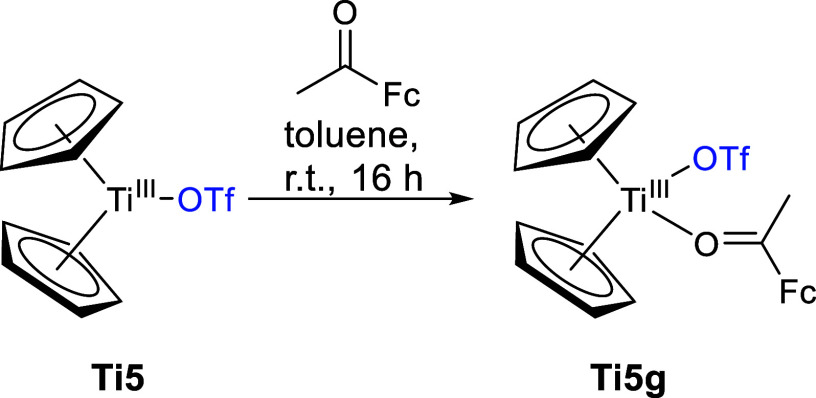
Reaction of **Ti5** with Acetyl Ferrocene to form **Ti5g**

The reaction with the sterically more demanding acetyl ferrocene (Scheme 10) does not produce the aldol condensation product but rather the ketone adduct **Ti5g** via coordination of the oxygen site.

**Figure 9:**
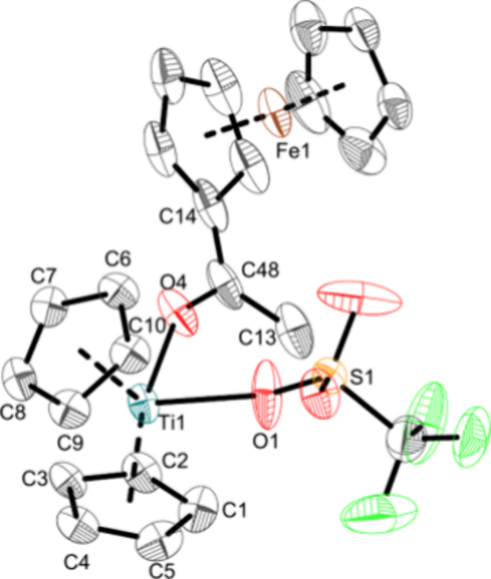
Molecular structure of complex **Ti5g**. Displacement ellipsoids are drawn at the 50% probability level. H atoms have been omitted for clarity. Selected bond lengths (Å) and angles (deg): Ti1—O1 2.163(3), Ti1—O4 2.104(2), S1—O1 1.437(3), C48—O4 1.243(3), C13—C48 1.490(5), C14—C48 1.446(5), O1—Ti1—O4 80.22(14), C48—O4—Ti1 150.2(2), Ct1—Ti1—Ct2 133.5 (Ct1 = centroid of C1–C5; Ct2 = centroid of C6–C10).

The molecular structure of **Ti5g** was confirmed by single-crystal X-ray diffraction (Figure 9).

The Ti–OTf bond (2.163(3) Å) is comparable to those in **Ti5a–e** and corresponds also to an elongated Ti–O single bond (Σr_cov_(Ti–O) = 1.99 Å).^36^ The Ti–O4 bond of the ketone adduct (2.104(2) Å) also corresponds to an elongated Ti–O single bond and is comparable with other titanium carbonyl adducts (2.100(3) Å,^63^ 2.146(2) Å,^64^ 2.111(1) Å^65^).

**Scheme 11:**
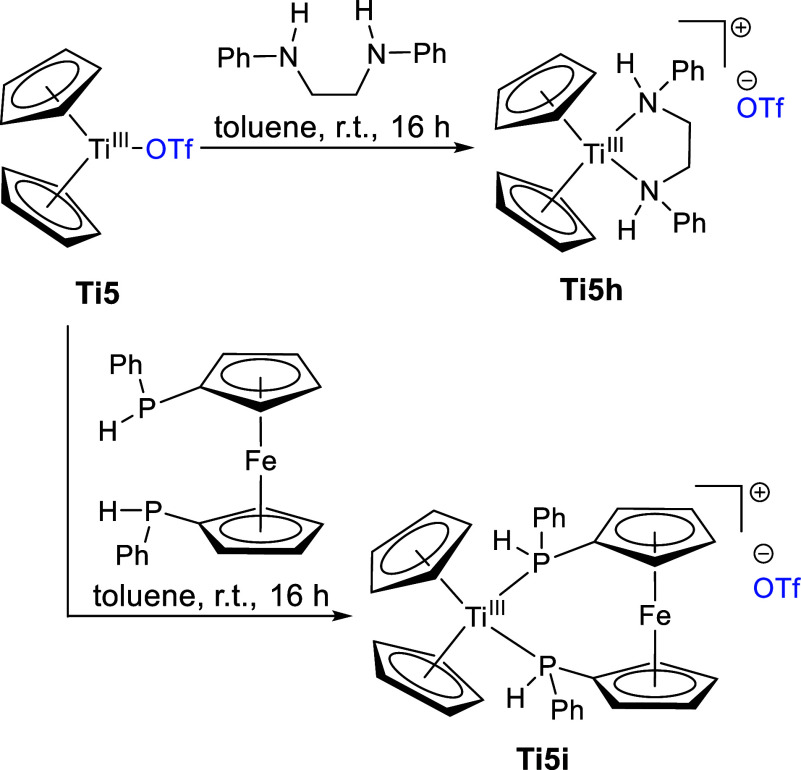
Reaction of **Ti5** with *N*,*N*′-Diphenylethylenediamine and 1,1′-Bis(phenylphosphine)ferrocene to Form **Ti5h**,**i**

The formation of **Ti 5f** indicates that bidentate ligands displace the triflato ligand due to its substitution labile nature and since we previously also encountered the displacement of the triflato ligand of **Ti5** by 2-pyridinecarboxaldehyde phenylhydrazone to form **Ti5x** (SI, Figure S19), we tested this hypothesis with several bidentate ligands (TMEDA, DPPE, DPPF), but only N,*N*′-diphenylethylenediamine and 1,1′-bis(phenylphosphine)ferrocene^66^ reacted with **Ti5** and led to the products **Ti5h** and **Ti5i** (Scheme 11).

**Figure 10:**
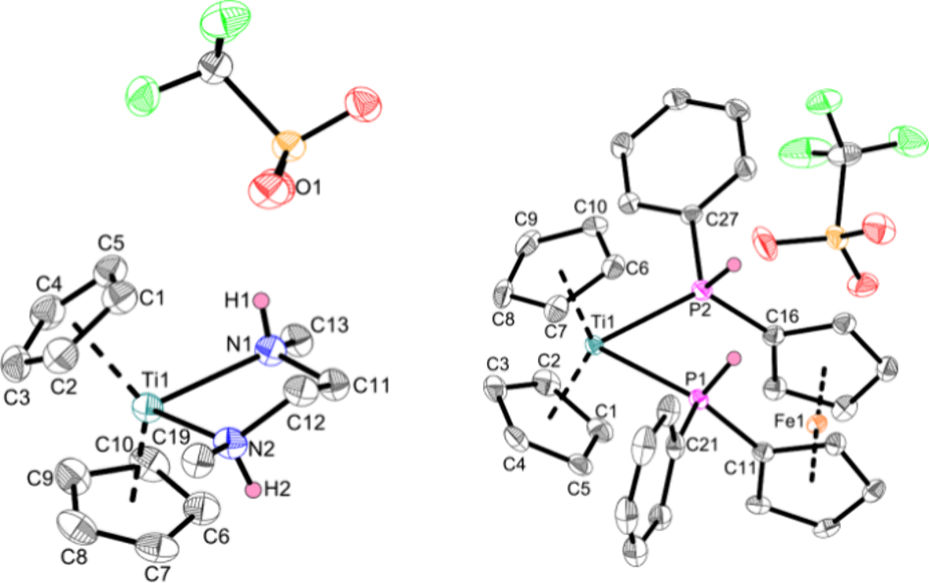
Crystal structures of complexes **Ti5h** (left) and **Ti5i** (right). Displacement ellipsoids are drawn at the 50% probability level. Redundant H atoms and phenyl rests of **Ti5h** have been omitted for clarity. Selected bond lengths (Å) and angles (deg): **Ti5h**, Ti1—N1 2.346(2), Ti1—N2 2.332(2), H1—O1 2.09(4), N1—Ti1—N2 74.16(8), Ct1—Ti1—Ct2 131.7; **Ti5i**, Ti1—P1 2.6003(10), Ti1—P2 2.6351(10), P1—C11 1.795(3), P2—C16 1.808(4), P1—Ti1—P2 76.21(3), Ct1—Ti1—Ct2 134.1 (Ct1 = centroid of C1–C5; Ct2 = centroid of C6–C10).

As confirmed by single-crystal X-ray diffraction of **Ti5h** and **Ti5i** (Figure 10), both bidentate ligands displace the triflato ligand from the coordination sphere of Ti and coordinate with both donor sides.

The Ti–N bonds (Ti1—N1:2.346(2) Å, Ti1—N2:2.332(2) Å) of **Ti5h** are best described as elongated single bonds according to the sum of covalent radii (Σr_cov_(Ti–N) = 2.07 Å)^36^ and are comparable to those in other diamine complexes (2.343(4) Å,^67^ 2.270(3) Å,^68^ 2.273(14) Å^69^). Complex **Ti5h** is an excellent example of the ambiguous supramolecular features caused by the triflate anion.^70−72^ The cationic titanium complexes are interconnected by the oxygen atoms of the triflate anions via NH hydrogen bonds (H1···O1 2.09(4) Å, H2···O2* 2.07(3) Å, * = generated by symmetry) (SI, Figure S20). In addition, CH···F interactions (2.54 Å, 2.73 Å) were observed between the Cp protons and the CF_3_ group of the triflate anion, further interconnecting the complexes (SI, Figure S21). These interactions are the reason for the outstanding crystallization properties of titanium triflato complexes.

**Ti5i** shows slightly asymmetrical Ti–P bonds (Ti1—P1:2.6003(10) Å, Ti1—P2:2.6351(10) Å), which are best described as elongated single bonds (Σr_cov_(Ti–P) = 2.47 Å).^36^ This feature is shared by other titanium complexes with chelating phosphine ligands (2.640(2) Å,^73^ 2.6312(9) Å,^74^ 2.604(1) Å^74^). Unlike **Ti5h**, there are no or only weak PH···O interactions between the cationic complex and the triflate anion. At the time of writing, **Ti5i** represents the first ferrocene phosphine-based titanium complex.

## Summary and Conclusions

In this work, we achieved highly effective syntheses of Ti(IV) and Ti(III) triflato complexes and demonstrated the possibilities and limitations of substituting the triflate anion. Reactions of the Ti(IV) complexes with water and NHC resulted in the displacement of the triflate anion out of the coordination sphere of the metal. The Ti(III) triflato complex is an effective Lewis acid and represents a brilliant titanocene(III) synthon for various reactions. The donor-free complex reacts with Lewis bases to form Lewis adducts and can be oxidized with TEMPO to form the respective Ti(IV) complex. It is capable of aldol reactions as demonstrated with acetone, and the use of bidentate ligands results in the displacement of the triflate anion. This concept could be expanded to other Lewis acidic metal complexes to synthesize cationic complexes without using classical weakly coordinating anions.

## Experimental Section

All reactions were carried out under a dry nitrogen or argon atmosphere using standard Schlenk and glovebox techniques. Caution! Extreme care should be taken both in the handling of cryogen liquid nitrogen and its use in the Schlenk line trap to avoid the condensation of oxygen from air. The pentafulvene complexes **I**, **II**, **V**, **VI**,^75,76^ the chloride complexes **III**, **IV**,^77^ the insertion products **VII**, **VIII**,^39^ and Cp_2_Ti(η^2^-Me_3_SiC_2_SiMe_3_)^78^ were prepared according to published procedures. Solvents were dried according to standard procedures over Na/K alloy with benzophenone as indicator and subsequently distilled and stored under a nitrogen atmosphere. NMR spectra were recorded on a Bruker AVANCE III 500 spectrometer (^1^H 500 MHz). IR spectra were recorded on a Bruker Tensor 27 spectrometer using an attenuated total reflection (ATR) method. Elemental analyses were carried out on a Euro EA 3000 Elemental Analyzer. Deviation in C and H values are due to the sensitivity of the compounds and possible carbide and nitride formation. Melting points were determined using a “Mel-Temp” from Laboratory Devices, Cambridge, or a Mettler Toledo MP30. High-resolution mass spectra were measured on a Thermo Fisher Scientific DFS Magnetic Sector HRMS System spectrometer using EI and LIFDI. Further exact details of syntheses, NMR spectra, crystallographic data, EPR spectra, and IR spectra are given in the Supporting Information (SI).

## Supporting Information Available

Syntheses, NMR spectra, crystallographic data, EPR spectra, and IR spectra (PDF)
