# Supplementary material for: From Coordination to Noncoordination: Syntheses and Substitution Lability Studies of Titanium Triflato Complexes
Source: Inorg Chem. 2024 Jul 26;63(31):14392–401. doi: 10.1021/acs.inorgchem.4c01033 (PMC11304387; doi:10.1021/acs.inorgchem.4c01033)
Supplement: Supplementary file 1 — ic4c01033_si_001.pdf [file ic4c01033_si_001.pdf]

Supporting Information

From Coordination to Non-Coordination: Syntheses and Substitution Lability Studies of Titanium Triflato Complexes.

Kevin Schwitalla, Zainab Yusufzadeh, Mark Schmidtmann and Rüdiger Beckhaus*

Chemistry Department, Carl von Ossietzky University of Oldenburg, 26111 Oldenburg, Germany

E-mail: ruediger.beckhaus@uol.de

TABLE OF CONTENTS

Synthesis and characterization of compounds.. 2

NMR spectra of complexes... 17

Additional molecular structures... 25

Supramolecular features.. 28

Crystallographic data.. 29

EPR spectra 34

IR spectra...39

References... 46

Synthesis and characterization of compounds

Synthesis of Ti1a:

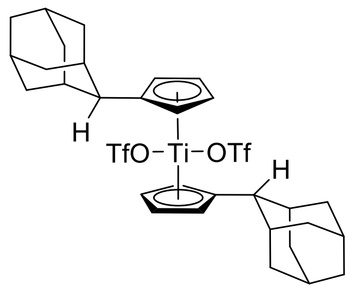

Route A: **III** (200 mg, 0.387 mmol) and AgOTf (198 mg, 0.773 mmol) were dissolved in 10 ml of dry toluene. The reaction mixture was stirred for 16 h at room temperature and the resulting suspension was filtered. The solvent was removed under reduced pressure and the residue was washed with *n*-hexane (3 x 5 mL). The residue was dried under vacuum to yield the product as a brown solid.

Yield: 110 mg, 0.148 mmol, 38 %.

Route B: **I** (200 mg, 0.387 mmol) was dissolved in 10 mL of dry diethyl ether and cooled to -90 °C. Trifluoromethanesulfonic acid (30.0 mL, 0.003 M in Et_2O, 0.900 mmol) was added dropwise to the stirred solution. The reaction mixture was slowly heated to room temperature and stirred for 16 h at room temperature. The resulting brown solid was isolated by separation of the supernatant and the residue was washed with 10 mL of *n*-hexane. The residue was dried under vacuum to yield the product as a brown solid. Red crystals suitable for single-crystal X-ray diffraction analysis precipitated from a slowly evaporating solution of **Ti1a** in C_6D_6 after several days.

Yield: 263 mg, 0.353 mmol, 91 %.

^{1}H NMR (500 MHz, 305 K, C_6D_6): δ = 1.24-2.22 (m, 28H, Ad-H), 2.85 (s, 2H, C_{exo}H), 6.31-6.35 (m, 4H, Cp-H), 6.47-6.51 (m, 4H, Cp-H) ppm.

^{13}C{^{1}H} NMR (126 MHz, 305 K, C_6D_6): δ = 27.6 (2 x Ad-CH), 27.8 (2 x Ad-CH), 31.7 (4 x Ad-CH), 32.3 (4 x Ad-CH_2), 37.6 (2 x Ad-CH_2), 38.0 (4 x Ad-CH_2), 44.0 (2 x C_{exo}H), 120.1 (q, $^1J_{CF}$ = 318 Hz, CF_3), 120.3 (Cp-CH), 124.2 (Cp-CH), 148.0 (2 x $C_{q,ipso}$) ppm.

^{19}F{^{1}H} NMR (470 MHz, 305 K, C_6D_6): δ = -76.8 ppm.

IR (ATR): ṽ = 3115, 2904, 2850, 1476, 1449, 1350, 1237, 1194, 1181, 1171, 1100, 999, 953, 842, 621, 594 cm^{-1}.

Melting point: 188 °C (dec.).

EA: calcd. for $C_{32}H_{38}F_6S_2O_6Ti$: C 51.62, H 5.14. Found: C 52.29, H 5.41.

HR/MS: calculated: m/z = 744.1493 [M+], measured (EI): m/z = 744.1482.

Synthesis of Ti1b:

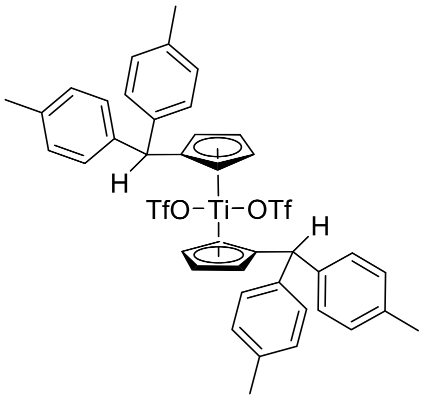

Route A: **IV** (1000 mg, 1.569 mmol) and AgOTf (806 mg, 3.138 mmol) were dissolved in 50 ml of dry toluene. The reaction mixture was stirred for 16 h at room temperature and the resulting suspension was filtered. The solvent was removed under reduced pressure and the residue was washed with *n*-hexane (3 x 5 mL). The residue was dried under vacuum to yield the product as a brown solid.

Yield: 788 mg, 0.911 mmol, 49 %.

Route B: **II** (200 mg, 0.314 mmol) was dissolved in 10 mL of dry diethyl ether and cooled to --90 °C. Trifluoromethanesulfonic acid (10.0 mL, 0.01 M in Et_2O, 0.708 mmol) was added slowly to the stirred solution. The reaction mixture was slowly heated to room temperature and stirred for 16 h at room temperature. The resulting brown solid was isolated by separation of the supernatant and the residue was washed with 10 mL of *n*-hexane. The residue was dried under vacuum to yield the product as a dark green solid.

Yield: 258 mg, 0.298 mmol, 95 %.

^{1}H NMR (500 MHz, 305 K, C_6D_6): δ = 2.10 (s, 12H, *p*-Tol-Me), 5.29 (s, 2H, C_{exo}H), 6.04-6.08 (m, 4H, Cp-H), 6.20-6.23 (m, 4H, Cp-H), 6.91-6.94 (m, 8H, Ar-H), 6.95-6.98 (m, 8H, Ar-H) ppm.

^{13}C{^{1}H} NMR (126 MHz, 305 K, C_6D_6): δ = 20.9 (4 x *p*-Tol-CH_3), 51.0 (2 x C_{exo}H), 120.0 (q, $^1J_{CF}$ = 318 Hz, CF_3), 122.7 (4 x Cp-CH), 123.6 (4 x Cp-CH), 129.0 (8 x Ar-CH), 129.8 (8 x Ar-CH), 137.1 (4 x Ar-C_q), 139.7 (4 x Ar-C_q), 146.6 (2 x $C_{ipso,q}$) ppm.

^{19}F{^{1}H} NMR (470 MHz, 305 K, C_6D_6): δ = -76.4 ppm.

IR (ATR): ṽ = 3116, 3051, 3026, 2991, 2924, 1510, 1348, 1296, 1236, 1192, 1153, 995, 969, 847, 824, 808, 762, 627, 594, 575 cm^{-1}.

Melting point: 117-120 °C (dec.).

EA: calcd. for $C_{42}H_{38}F_6S_2O_6Ti$: C 58.34, H 4.43. Found: C 55.22, H 4.33.

Synthesis of Ti2b:

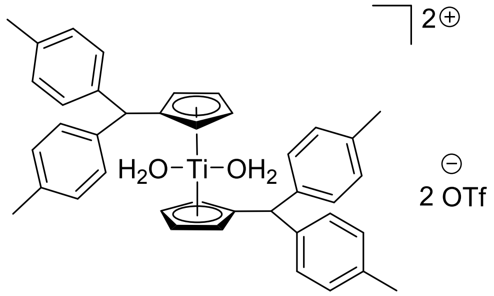

Ti1b (200 mg, 0.231 mmol) was dissolved in 50 mL of dry toluene. Degassed water (0.46 mL, 1 M in THF, 0.463 mmol) was added slowly to the stirred solution. The reaction mixture was stirred for 3 h at room temperature. The resulting yellow solid was isolated by separation of the supernatant and the residue was washed with 10 mL of *n*-hexane. The residue was dried under vacuum to yield the product as a yellow solid. Caution: The obtained solid is only stable for a couple of hours and decomposes back to **Ti1b** over time. Yellow crystals suitable for single-crystal X-ray diffraction analysis precipitated from a slowly evaporating solution of **Ti2b** in benzene after several days.

Yield: 138 mg, 0.156 mmol, 68 %.

^{1}H NMR (500.0 MHz, 305.0 K, THF-D_8): δ = 2.28 (s, 12H, p-Tol-Me), 5.14 (s, 2H, C_{exo}H), 5.98-6.07 (m, 4H, Cp-H), 6.68-6.78 (m, 4H, Cp-H), 6.92-6.97 (m, 8H, Ar-H), 7.08-7.11 (m, 8H, Ar-H), 8.88 (s (br), 4H, H_2O), ppm.

^{13}C{^{1}H} NMR (126 MHz, 305 K, THF-D_8): δ = 21.1 (4 x *p*-Tol-CH_3), 51.7 (2 x C_{exo}H), 126.2 (4 x Ar-C_q), 129.1 (4 x Cp-CH), 129.7 (8 x Ar-CH), 129.8 (4 x Cp-CH), 130.3 (8 x Ar-CH), 137.8 (Ar- C_q), 138.6 ($C_{ipso,q}$) ppm. The CF_3 group of the triflate anion could not be detected.

^{19}F{^{1}H} NMR (470 MHz, 305 K, THF-D_8): δ = -78.9 ppm.

Due to the presence of a second species in the ^{19}F{^{1}H} NMR spectrum (**Figure S7**) and the general instability of the solid, further characterization was omitted.

Synthesis of Ti3a:

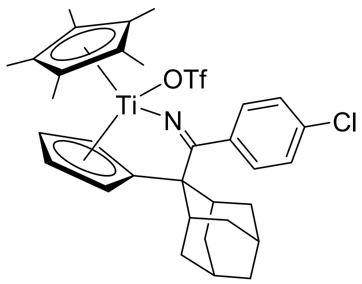

VII (360 mg, 0.649 mmol) and AgOTf (167 mg, 0.649 mmol) were dissolved in 15 mL of dry toluene and stirred for 16 h at room temperature. The resulting mixture was filtered and the solvent was removed under reduced pressure. The residue was washed with 5 mL of *n*-hexane andwas dried under vacuum to yield the product as a red solid. Red crystals suitable for single-crystal X-ray diffraction analysis precipitated from a saturated toluene solution of **Ti3a** after several days.

Yield: 310 mg, 0.464 mmol, 72 %.

^{1}H NMR (500 MHz, 305 K, C_6D_6): δ = 1.02-2.51 (m, 14H, Ad-H), 1.69 (s, 15H, Cp*-Me), 4.99-5.02 (m, 1H, Cp-H), 5.43-5.46 (m, 1H, Cp-H), 6.28-6.33 (m, 1H, Cp-H), 6.71-6.98 (m (br), 1H, Ar-CH), 7.03-7.05 (m, 1H, Cp-H), 7.10-7.15 (m , 2H, Ar-CH), 7.33-7.65 (m (br), 1H, Ar-CH) ppm.

^{13}C{^{1}H} NMR (126 MHz, 305 K, C_6D_6): δ = 12.3 (5 x Cp*-CH_3), 27.4 (Ad-CH), 27.6 (Ad-CH), 32.7 (Ad-CH_2), 33.4 (Ad-CH), 33.8 (Ad-CH_2), 34.5 (Ad-CH_2), 35.2 (Ad-CH_2), 36.4 (Ad-CH), 38.5 (Ad-CH_2), 60.0 ($C_{exo,q}$), 108.8 (Cp-CH), 110.2 (Cp-CH), 114.3 (Cp-CH), 117.1 (Cp-CH), 120.3 (q, $^1J_{CF}$ = 319 Hz, CF_3), 125.2 (5 x Cp*-C_q), 125.7 (2 x Ar-CH), 129.3 (2 x Ar-CH), 133.9 (Ar-C_q), 140.6 (Ar-C_q), 152.6 ($C_{ipso,q}$), 203.8 (C=N) ppm.

^{19}F{^{1}H} NMR (470 MHz, 305 K, C_6D_6): δ = -77.0 ppm.

IR (ATR): ṽ = 2910, 2859, 1594, 1486, 1454, 1378, 1328, 1280, 1258, 1233, 1207, 1179, 1160, 1089, 1074, 1027, 835, 810, 792, 762, 739, 712, 675, 630, 608, 590, 571, 518 cm^{-1}.

Melting point: 130 - 135 °C.

EA: calcd. for $C_{33}H_{37}ClF_3NSO_3Ti$: C 59.33, H 5.58. Found: C 58.31, H 5.89.

Synthesis of Ti4a:

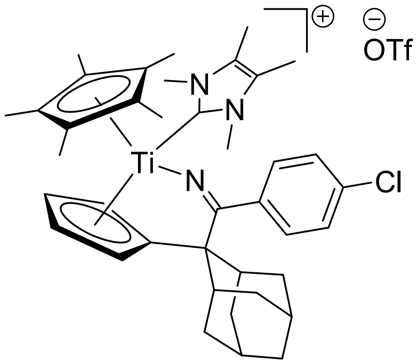

Ti3a (30 mg, 0.045 mmol) and 1,3,4,5-Tetramethylimidazol-2-ylidene (5.6 mg, 0.045 mmol) were dissolved in 0.7 mL of dry C_6D_6. Red-orange crystals suitable for single-crystal X-ray diffraction analysis precipitated from the C_6D_6 solution of **Ti4a** after several days.

Yield: quantitative (NMR-scale).

^{1}H NMR (500 MHz, 305 K, C_6D_6): δ = 1.23-3.01 (m, 14H, Ad-H), 1.64 (s, 15H, Cp*-Me), 1.75 (s, 3H, NHC-Me), 1.90 (s, 3H, NHC-Me), 2.73 (s, 3H, NHC-Me), 3.21 (s, 3H, NHC-Me), 5.48-5.50 (m, 1H, Cp-H), 5.74-5.76 (m, 1H, Cp-H), 6.57-6.62 (m, 2H, Cp-H), 6.82-6.87 (m, 1H, Ar-H), 7.17-7.20 (m , 1H, Ar-CH), 7.52-7.58 (m, 1H, Ar-CH), 7.84-7.90 (m, 1H, Ar-CH) ppm.

^{13}C{^{1}H} NMR (126 MHz, 305 K, C_6D_6): δ = 8.9 (NHC-CH_3), 9.4 (NHC-CH_3), 12.5 (5 x Cp*-CH_3), 27.6 (Ad-CH), 27.6 (Ad-CH), 32.9 (Ad-CH_2), 33.1 (Ad-CH), 33.9 (Ad-CH_2), 34.1 (Ad-CH_2), 34.8 (Ad-CH_2), 35.5 (NHC-CH_3), 36.0 (Ad-CH), 37.6 (NHC-CH_3), 38.4 (Ad-CH_2), 60.4 ($C_{exo,q}$), 104.9 (Cp-CH), 111.0 (Cp-CH), 111.8 (Cp-CH), 120.7 (Cp-CH), 123.1 (5 x Cp*-C_q), 125.2 (Ar-CH), 126.5 (Ar-CH), 128.8 (Ar-CH), 129.0 (Ar-CH), 134.4 (Ar-C_q), 135.6 (2 x NHC-C_q-CH_3), 140.8 (Ar-C_q), 154.4 ($C_{ipso,q}$), 183.8 (N-C_q-N), 205.6 (C=N) ppm. The CF_3 group of the triflate anion could not be detected.

^{19}F{^{1}H} NMR (470 MHz, 305 K, C_6D_6): δ = -77.8 ppm.

Synthesis of Ti5:

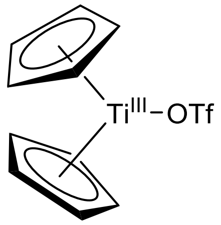

Route A: Titanocene bis(trimethylsilyl)acetylene (1.00 g, 2.87 mmol) and silver(I) trifluoromethanesulfonate (737 mg, 2.87 mmol) were dissolved in 40 ml of dry toluene. The reaction mixture was stirred for 1 h at room temperature to give a dark green solution. Ag(0) was removed by filtration and the residue was washed with toluene (3 x 20 mL) and filtered. The solvent was removed under reduced pressure and the residue was dried under vacuum to yield the product as a green blue solid. Crystals of **Ti5** suitable for single-crystal X-ray diffraction analysis were obtained by storing the toluene solution of **Ti5** at -20 °C over night, forming greenish blue crystals.

Yield: 0.725 g, 2.22 mmol, 77%.

Route B: Titanocene bis(trimethylsilyl)acetylene (500 mg, 1.43 mmol) was dissolved in 20 ml of dry *n*-hexane. The resulting solution was cooled to -80 °C and trifluoromethanesulfonic acid (14.3 mL, 0.1 M in Et_2O, 1.43 mmol) was added slowly to the stirred solution. The reaction mixture was stirred for 1 h at -80 °C to give an orange red suspension. After warming to room temperature and stirring for an additional hour, the reaction mixture was stored at -20 °C over night. A pale green solid precipitated which was isolated by separation of the supernatant. The residue was dried under vacuum to yield the product as a green blue solid.

Yield: 0.345 g, 1.05 mmol, 74%.

IR (ATR): $\tilde{v}$ = 3113, 1443, 1313, 1234, 1197, 1178, 1124, 1066, 1034, 1019, 808, 765, 704, 622, 585, 512 cm^{-1}.

Melting point: 250 °C (dec.).

HR/MS: calculated: m/z = 326.9782 [M+], measured (LIFDI): m/z = 326.9777.

EA: calcd. for $C_{22}H_{20}F_6S_2O_6Ti_2$: C 40.39, H 3.08. Found: C 40.58, H 2.88.

EPR: g = 1.944

Synthesis of Ti5a:

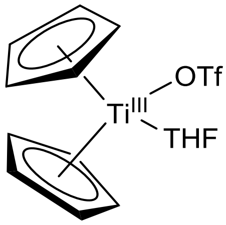

Titanocene triflate **Ti5** (100 mg, 0.306 mmol) was dissolved in 5 ml of THF and the reaction mixture was stirred for 1 h at room temperature to give a green blue solution. The solvent was removed under reduced pressure and the residue was dried under vacuum to yield the product as a blue solid. Crystals of **Ti5a** suitable for single-crystal X-ray diffraction analysis were obtained by storing the THF solution of **Ti5a** at -20 °C over night, forming blue crystals.

Yield: 92 mg, 0.230 mmol, 75%.

IR (ATR): $\tilde{\nu}$ = 3114, 1444, 1317, 1234, 1209, 1178, 1065, 1015, 861, 829, 804, 759, 707, 628, 584 cm^{-1}.

Melting point: 260 °C (dec.).

EA: calcd. for $C_{15}H_{18}F_3SO_4Ti$: C 45.13, H 4.54. Found: C 43.61, H 4.42.

EPR: g = 1.953

Synthesis of Ti5b:

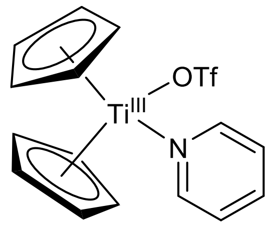

Route A: Titanocene triflate **Ti5** (100 mg, 0.306 mmol) was dissolved in 5 ml of dry toluene. 0.1 mL of pyridine were added and the reaction mixture was stirred for 1 h at room temperature to give a green solution. The solvent was removed under reduced pressure and the residue was dried under vacuum to yield the product as a green solid.

Yield: 99 mg, 0.248 mmol, 81%.

Route B: Titanocene bis(trimethylsilyl)acetylene (200 mg, 0.574 mmol) and pyridinium trifluoromethanesulfonat (132 mg, 0.574 mmol) were dissolved in 10 ml of dry toluene. The reaction mixture was stirred for 2 h at room temperature to give a yellow green solution. Green crystals precipitated from the reaction mixture. The solid was isolated by separation of the supernatant and dried under vacuum to yield the product as a green solid. Crystals of **Ti5b** suitable for single-crystal X-ray diffraction analysis were obtained by storing the toluene solution of **Ti5b** at -20 °C over night, forming green crystals.

Yield: 194 mg, 0.478 mmol, 83%.

IR (ATR): $\tilde{\nu}$ = 3111, 1604, 1489, 1447, 1316, 1235, 1208, 1175, 1069, 1016, 927, 808, 759, 701, 630, 585, 568 cm^{-1}.

Melting point: 157 °C (dec.).

HR/MS: calculated: m/z = 406.0204 [M+], measured (LIFDI): m/z = 406.0199.

EA: calcd. for $C_{16}H_{15}F_3NSO_3Ti$: C 47.31, H 3.72. Found: C 46.26, H 3.68

EPR: g = 1.961

Synthesis of Ti5c:

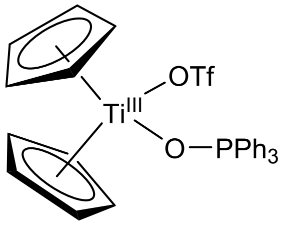

Titanocene triflate **Ti5** (100 mg, 0.306 mmol) and triphenylphophine oxide (85.1 mg, 0.306 mmol) were dissolved in 5 ml of dry toluene. The reaction mixture was stirred for 2 h at room temperature to give a green suspension. Green crystals precipitated from the reaction mixture. The solid was isolated by separation of the supernatant and dried under vacuum to yield the product as a green solid. Crystals of **Ti5c** suitable for single-crystal X-ray diffraction analysis were obtained by storing the toluene solution of **Ti5c** at -20 °C over night, forming green crystals.

Yield: 0.151 g, 0.249 mmol, 82%.

IR (ATR): $\tilde{\nu}$ = 1590, 1485, 1439, 1306, 1237, 1225, 1174, 1118, 1096, 1072, 1032, 808, 759, 722, 692, 632, 582 cm^{-1}.

Melting point: 238 °C (dec.).

EA: calcd. for $C_{29}H_{25}F_3SO_4PTi$: C 57.53, H 4.16. Found: C 58.10, H 3.93.

EPR: g = 1.957

Synthesis of Ti5d:

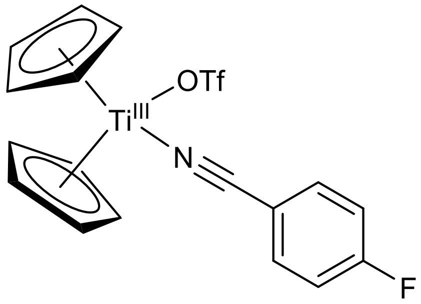

Titanocene triflate **Ti5** (100 mg, 0.306 mmol) and 4-flourobenzonitrile (37 mg, 0.306 mmol) were dissolved in 5 ml of dry toluene. The reaction mixture was stirred for 2 h at room temperature to give a yellow solution. Green crystals precipitated from the reaction mixture. The solid was isolated by separation of the supernatant and dried under vacuum to yield the product as a green solid. Crystals of **Ti5d** suitable for single-crystal X-ray diffraction analysis were obtained by storing the toluene solution of **Ti5d** at -20 °C over night, forming green crystals.

Yield: 92 mg, 0.205 mmol, 67%.

IR (ATR): $\tilde{\nu}$ = 3110, 2257, 1601, 1506, 1439, 1410, 1307, 1222, 1156, 1065, 1026, 1011, 818, 760, 706, 629, 585 cm^{-1}.

Melting point: 149 °C (dec.).

HR/MS: calculated: m/z = 448.0110 [M+], measured (LIFDI): m/z = 448.0105.

EA: calcd. for $C_{18}H_{14}F_4NSO_3Ti$: C 48.23, H 3.15. Found: C 48.05, H 3.17.

EPR: g = 1.961

Synthesis of Ti5e:

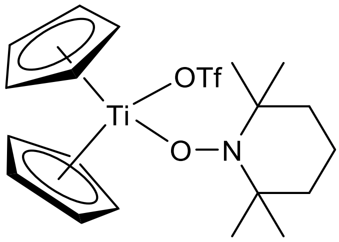

Titanocene triflate **Ti5** (100 mg, 0.306 mmol) and TEMPO (47.8 mg, 0.306 mmol) were dissolved in 5 ml of dry toluene. The reaction mixture was stirred for 2 h at room temperature to give a brown yellow solution. The solvent was removed under reduced pressure. The residue was washed with *n*-hexane and dried under vacuum to yield the product as a yellow solid. Yellow crystals suitable for single-crystal X-ray diffraction analysis precipitated from a slowly evaporating solution of **Ti5e** in benzene after several days.

Yield: 106 mg, 0.219 mmol, 72%.

^{1}H NMR (500 MHz, 305 K, C_6D_6): δ = 0.95 (s, 12H, Me), 1.15-1.23 (m, 2H, CH_2-**<u>CH_2</u>**-CH_2), 1.32-1.35 (s, 4H, **<u>CH_2</u>**-CH_2-**<u>CH_2</u>**), 6.16 (s, 10H, Cp-H) ppm.

^{13}C{^{1}H} NMR (125 MHz, 305 K, C_6D_6): 16.9 (CH_2-**<u>CH_2</u>**-CH_2), 40.7 (**<u>CH_2</u>**-CH_2-**<u>CH_2</u>**), 62.5 (2 x N-C_qMe_2), 118.2 (10 x Cp-CH), 120.3 (q, $^{1}J_{CF}$ = 319 Hz, CF_3). The methyl groups could not be detected.

^{19}F{^{1}H} NMR (470 MHz, 305 K, C_6D_6): δ = -77.6 ppm.

IR (ATR): ṽ = 3128, 2990, 2964, 2932, 1454, 1375, 1361, 1332, 1234, 1203, 1174, 1130, 1072, 1021, 976, 956, 927, 879, 842, 822, 804, 762, 694, 652, 630, 587 cm^{-1}.

Melting point: 147 °C (dec.).

HR/MS: calculated: m/z = 483.1171 [M+], measured (LIFDI): m/z = 483.1165.

EA: calcd. for $C_{20}H_{28}F_3NSO_4Ti$: C 49.70, H 5.84. Found: C 49.75, H 5.14.

Synthesis of Ti5f:

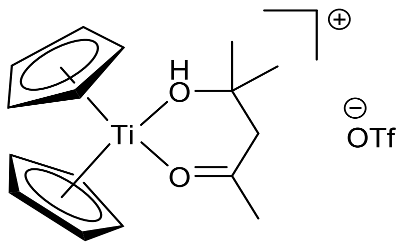

Titanocene triflate **Ti5** (100 mg, 0.306 mmol) and acetone (0.1 mL, 1.380 mmol) were dissolved in 10 ml of dry toluene. The reaction mixture was stirred for 2 h at room temperature to give a green solution. The solvent was removed under reduced pressure. The residue was washed with *n*-hexane and dried under vacuum to yield the product as a pale green solid. Crystals of **Ti5f** suitable for single-crystal X-ray diffraction analysis were obtained by a saturated toluene solution of **Ti5f** at -20 °C, forming green crystals.

Yield: 107 mg, 0.241 mmol, 79%.

IR (ATR): $\tilde{\nu}$ = 3112, 2979, 1669, 1442, 1407, 1392, 1374, 1314, 1287, 1234, 1223, 1203, 1163, 1067, 1026, 1020, 910, 808, 764, 707, 630, 622, 594, 571, 514 cm^{-1}.

Melting point: 106 °C (dec.).

EA: calcd. for $C_{17}H_{22}F_3SO_5Ti$: C 46.06, H 5.00. Found: C 46.64, H 5.07.

EPR: g = 1.970

Synthesis of Ti5g:

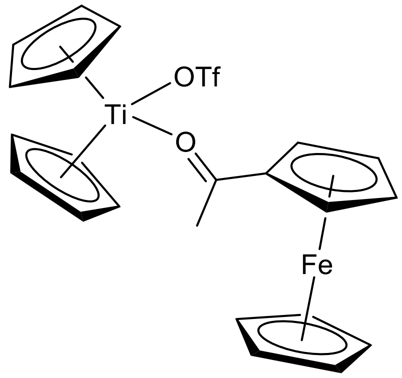

Titanocene triflate **Ti5** (100 mg, 0.306 mmol) and acetyl ferrocene (69.7 mg, 0.306 mmol) were dissolved in 10 ml of dry toluene. The reaction mixture was stirred for 16 h at room temperature to give a dark red solution. The solvent was removed under reduced pressure. The residue was washed with *n*-hexane and dried under vacuum to yield the product as a red solid. Red crystals suitable for single-crystal X-ray diffraction analysis precipitated from a slowly evaporating solution of **Ti5g** in benzene after several days.

Yield: 137 mg, 0.247 mmol, 81%.

IR (ATR): $\tilde{\nu}$ = 3105, 1662, 1600, 1557, 1456, 1414, 1381, 1361, 1333, 1305, 1293, 1277, 1236, 1211, 1163, 1115, 1107, 1028, 894, 815, 799, 762, 730, 670, 631, 593, 585, 571, 553 cm^{-1}.

Melting point: 88 °C (dec.).

EA: calcd. for $C_{23}H_{22}F_3FeSO_4Ti$: C 49.76, H 3.99. Found: C 49.98, H 4.30.

EPR: g = 1.973

Synthesis of Ti5h:

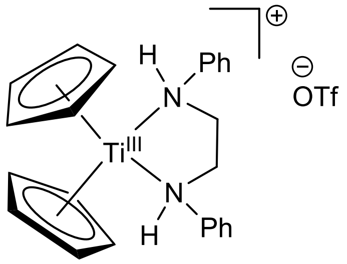

Titanocene triflate **Ti5** (100 mg, 0.306 mmol) and N,N'-Diphenylethylenediamine (64.9 mg, 0.306 mmol) were dissolved in 10 ml of dry toluene. The reaction mixture was stirred for 16 h at room temperature to give a blue suspension. The solid was isolated by separation of the supernatant and dried under vacuum to yield the product as a blue solid. Crystals of **Ti5h** suitable for single-crystal X-ray diffraction analysis were obtained by a saturated toluene solution of **Ti5h** at room temperature, forming blue crystals.

Yield: 129 mg, 0.239 mmol, 78%.

IR (ATR): ṽ = 3231, 1601, 1495, 1466, 1436, 1268, 1256, 1233, 1225, 1206, 1176, 1167, 1156, 1084, 1068, 1058, 1028, 1012, 1004, 993, 830, 821, 813, 798, 751, 687, 637, 619, 575, 523, 516 cm^{-1}.

Melting point: 177 °C (dec.).

EA: calcd. for $C_{25}H_{26}F_3N_2SO_3Ti$: C 55.67, H 4.86. Found: C 54.92, H 4.79

EPR: g = 1.973

Synthesis of Ti5i:

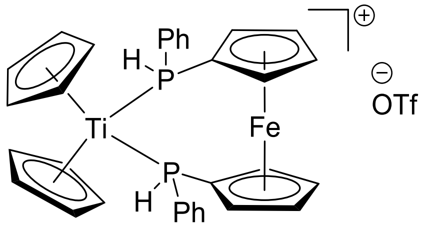

Titanocene triflate **Ti5** (50 mg, 0.153 mmol) and 1,1´-bis (phenylphosphine)ferrocene (61.5 mg, 0.153 mmol) were dissolved in 5 ml of dry toluene. The reaction mixture was stirred for 16 h at room temperature to give a yellow suspension. The solid was isolated by separation of the supernatant and dried under vacuum to yield the product as a yellow solid. Crystals of **Ti5i** suitable for single-crystal X-ray diffraction analysis were obtained by a saturated toluene solution of **Ti5i** at room temperature, forming yellow crystals.

Yield: 82 mg, 0.112 mmol, 74%.

IR (ATR): ṽ = 3112, 1436, 1282, 1259, 1225, 1165, 1147, 1098, 1073, 1030, 1012, 933, 880, 818, 745, 694, 636, 573, 517 cm^{-1}.

Melting point: 176 °C (dec.).

EA: calcd. for $C_{33}H_{30}F_3FeP_2SO_3Ti$: C 54.35, H 4.15. Found: C 54.57 H 3.91.

EPR: g = 1.974

NMR Spectra of complexes

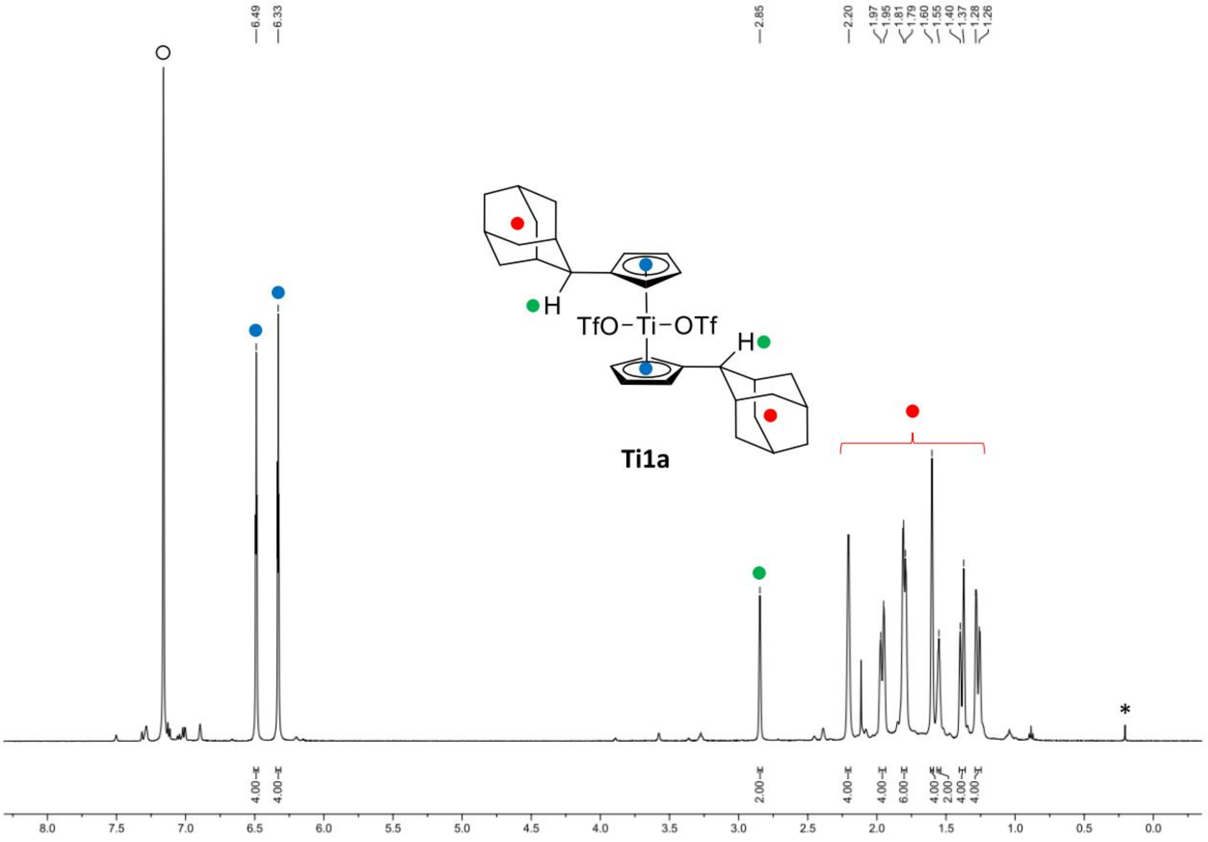

Figure S1: ^{1}H NMR spectrum (500 MHz, C_6D_6, 305 K) of **Ti1a**. Product signals given in colours (° = C_6D_5H, * = grease).

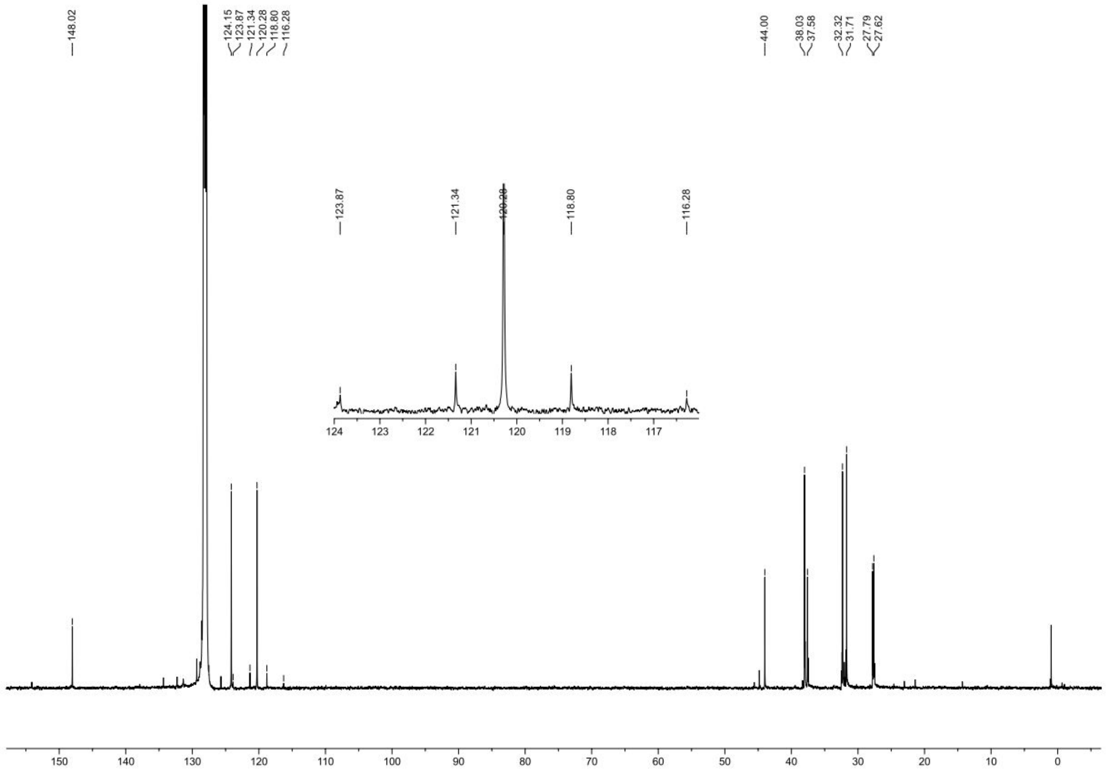

Figure S2: ^{13}C NMR spectrum (126 MHz, C_6D_6, 305 K) of **Ti1a**.

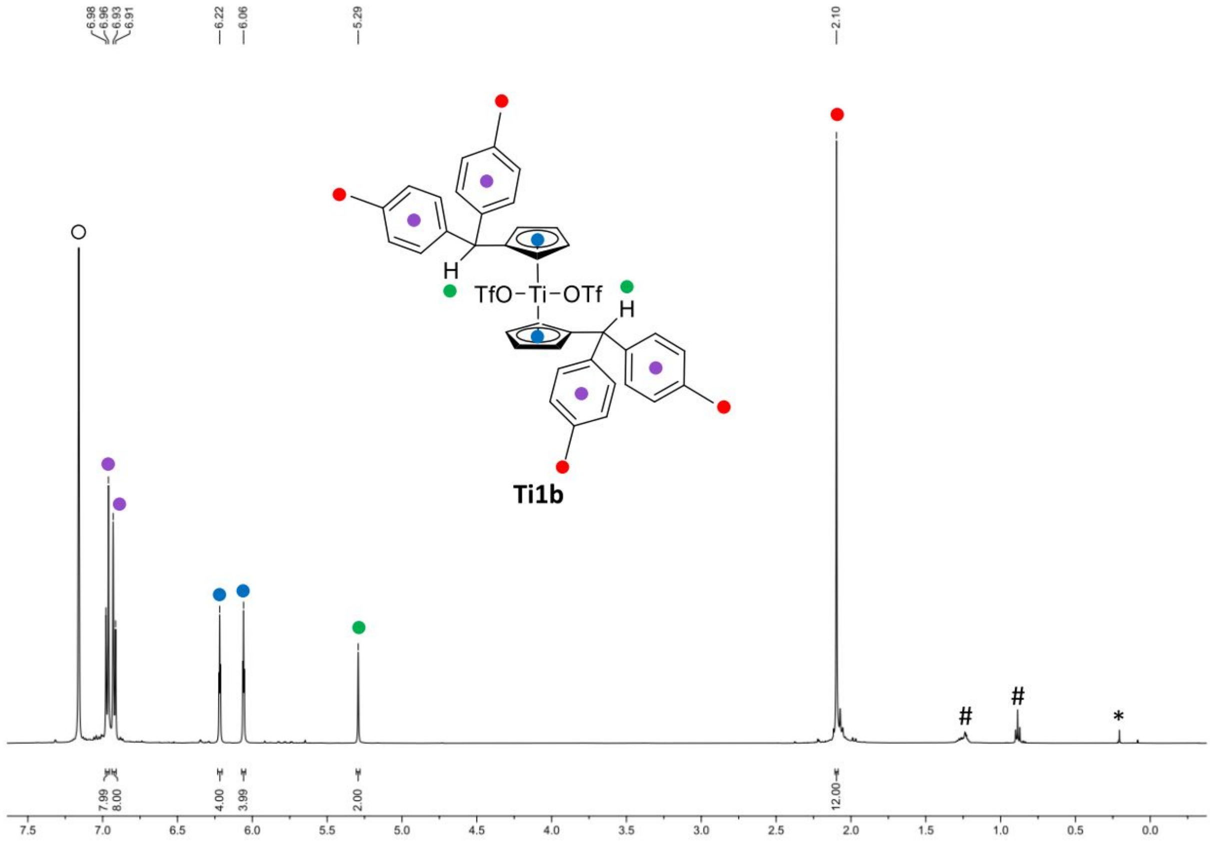

Figure S3: ^{1}H NMR spectrum (500 MHz, C_6D_6, 305 K) of **Ti1b**. Product signals given in colours (^{o} = C_6D_5H, # = *n*-hexane, * = grease).

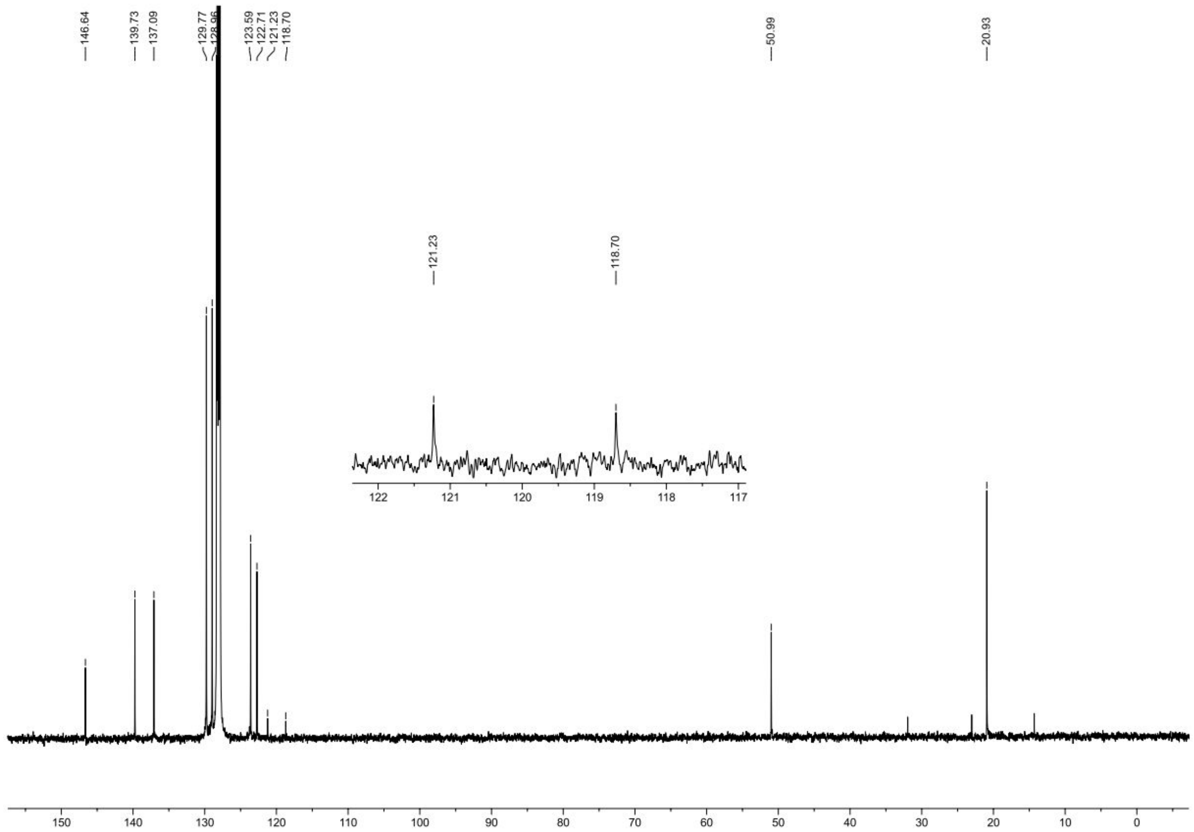

Figure S4: ^{13}C NMR spectrum (126 MHz, C_6D_6, 305 K) of **Ti1b**.

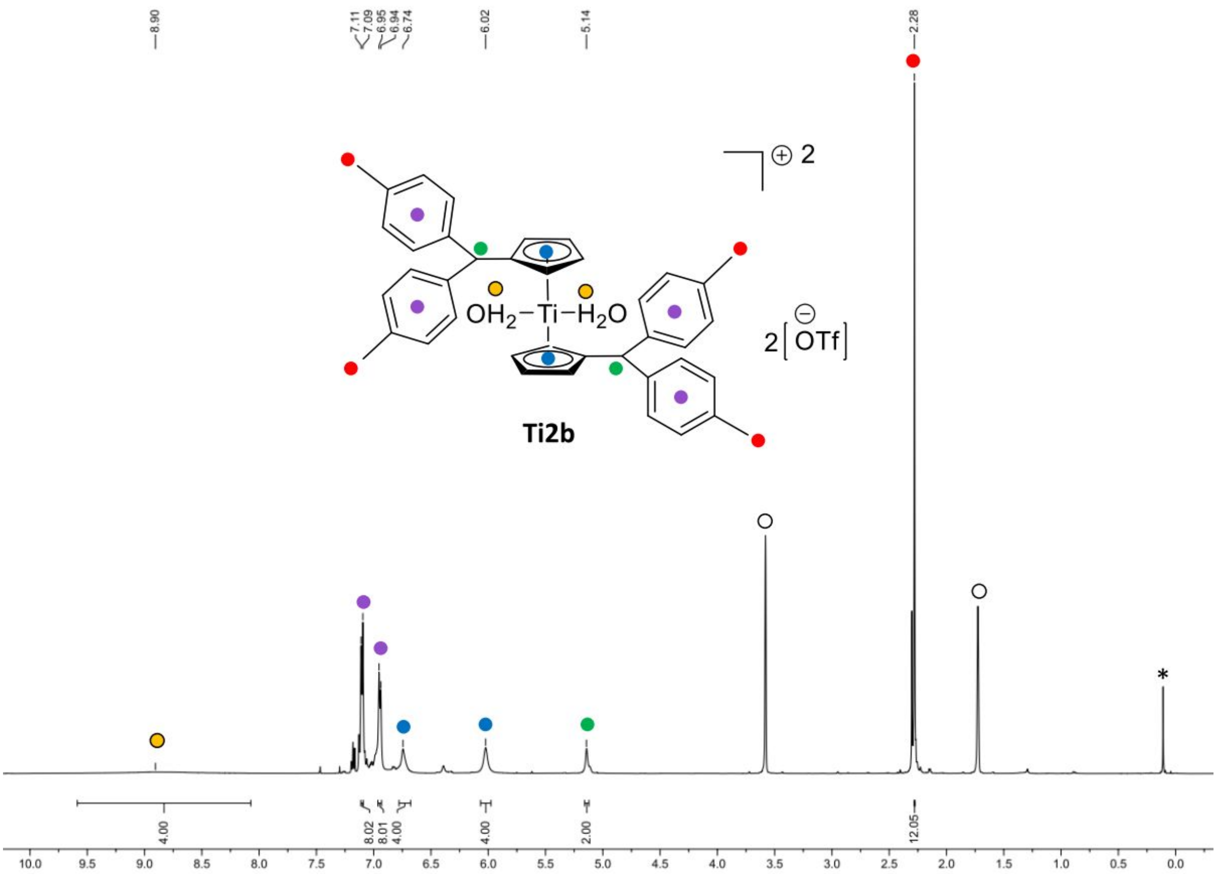

Figure S5: ^{1}H NMR spectrum (500 MHz, THF-D_8, 305 K) of **Ti2b**. Product signals given in colours (^{o} = THF-D_7H, * = grease).

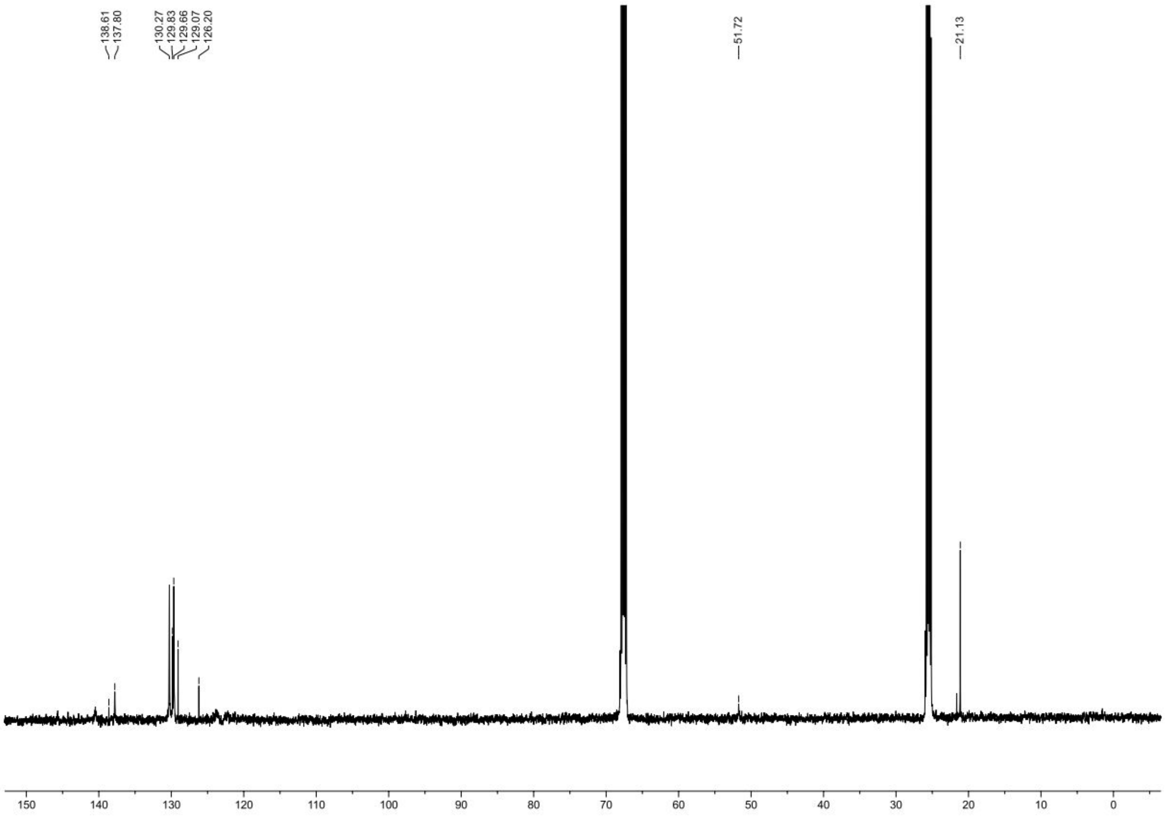

Figure S6: ^{13}C NMR spectrum (126 MHz, THF-D_8, 305 K) of **Ti2b**.

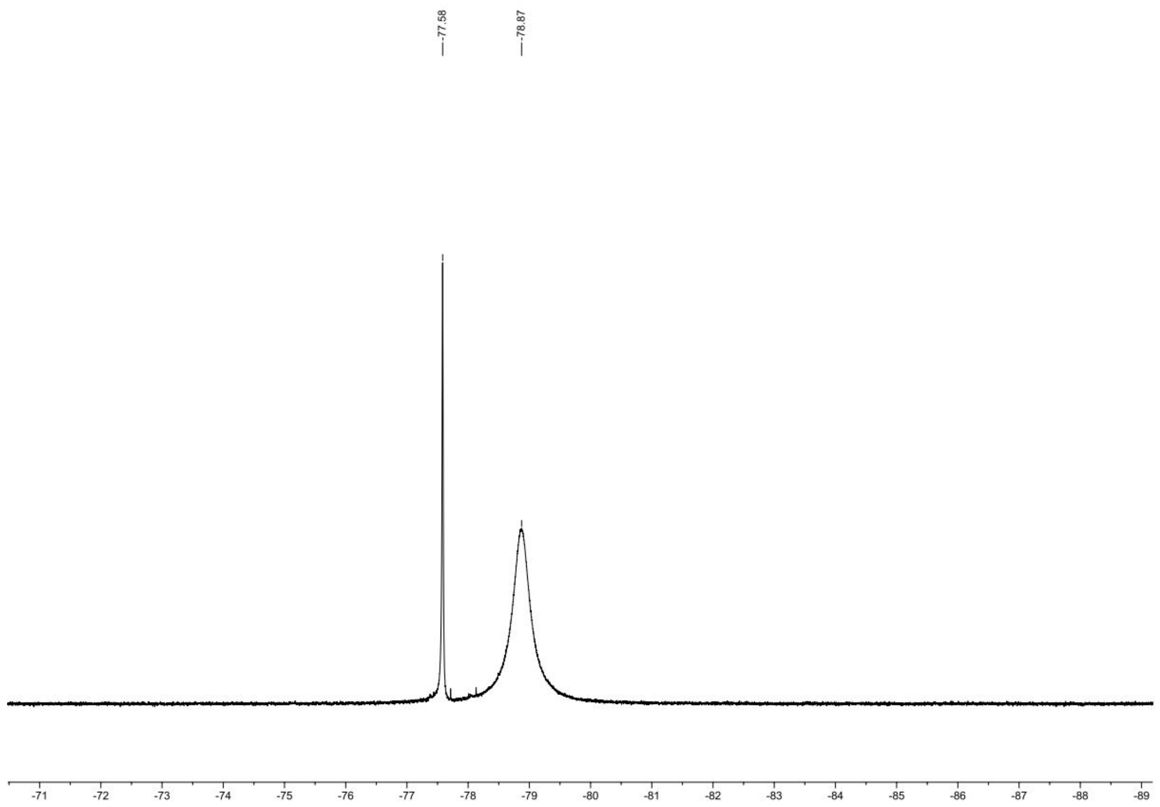

Figure S7: $^{19}F\{^{1}H\}$ NMR spectrum (470 MHz, THF-D_8, 305 K) of **Ti2b**.

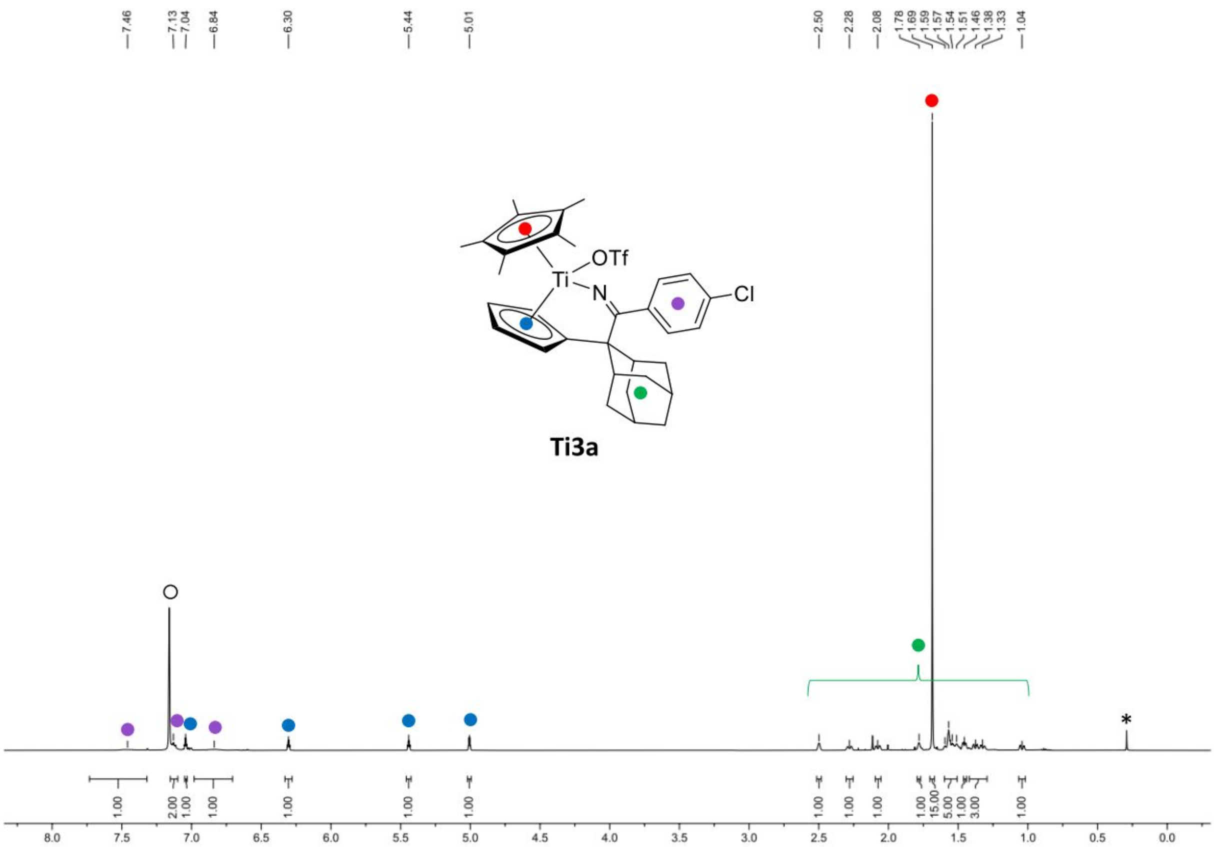

Figure S8: ^{1}H NMR spectrum (500 MHz, C_6D_6, 305 K) of **Ti3a**. Product signals given in colours (^{o} = C_6D_5H, * = grease).

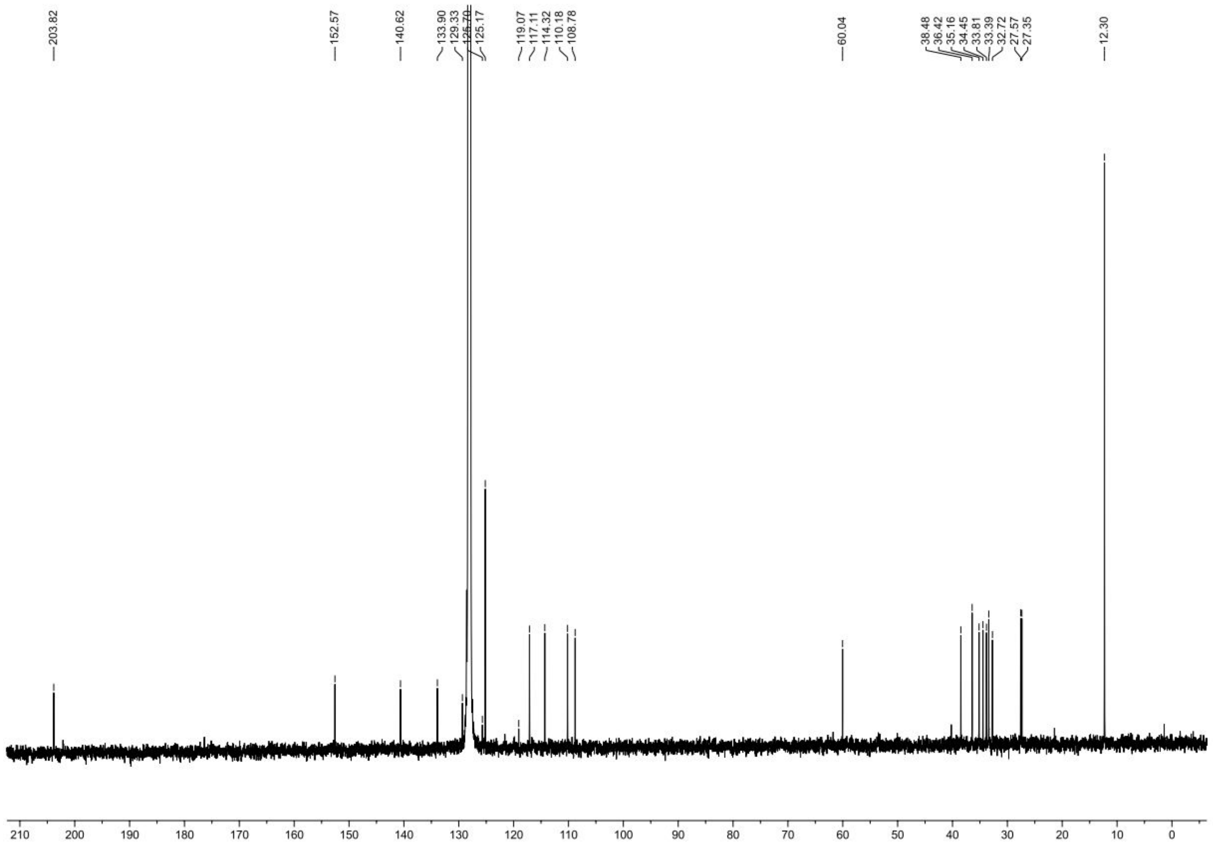

Figure S9: ^{13}C NMR spectrum (126 MHz, C_6D_6, 305 K) of **Ti3a**.

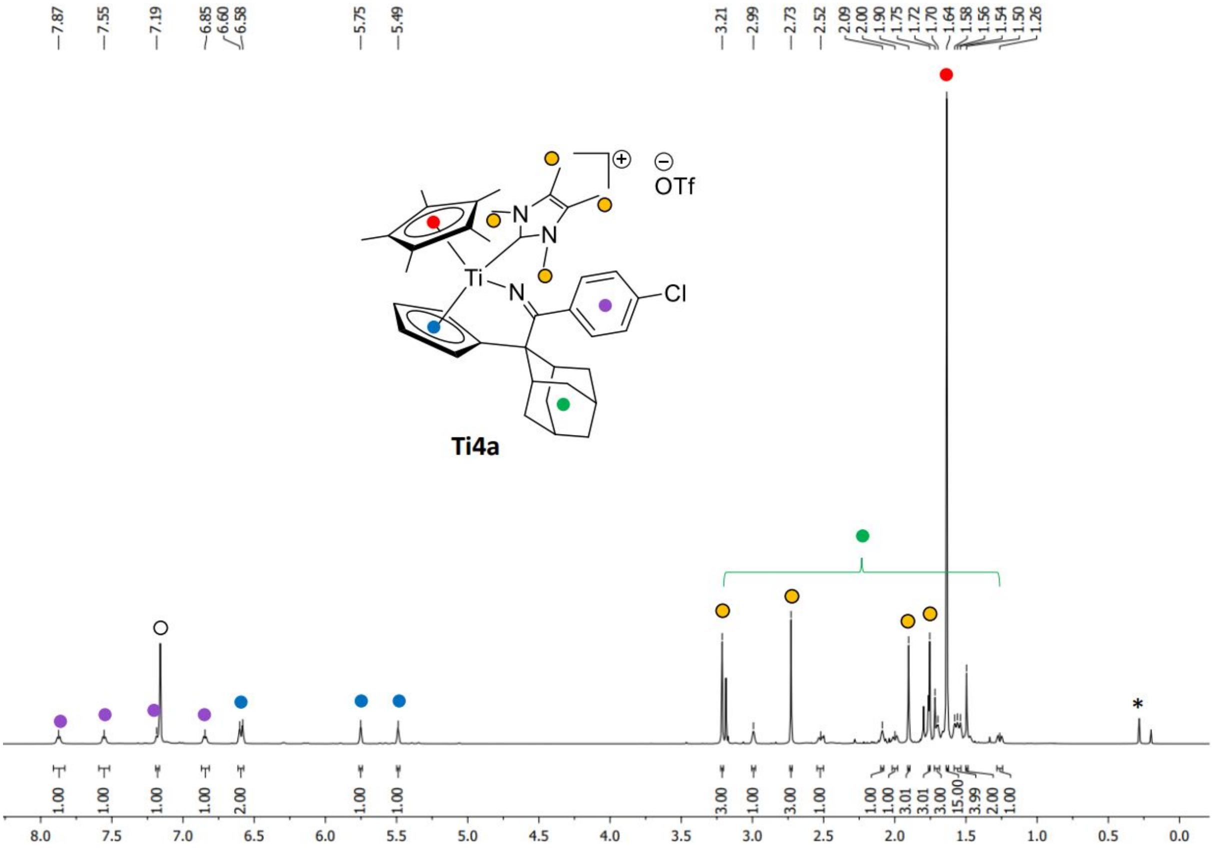

Figure S10: ^{1}H NMR spectrum (500 MHz, C_6D_6, 305 K) of **Ti4a**. Product signals given in colours (° = C_6D_5H, * = grease).

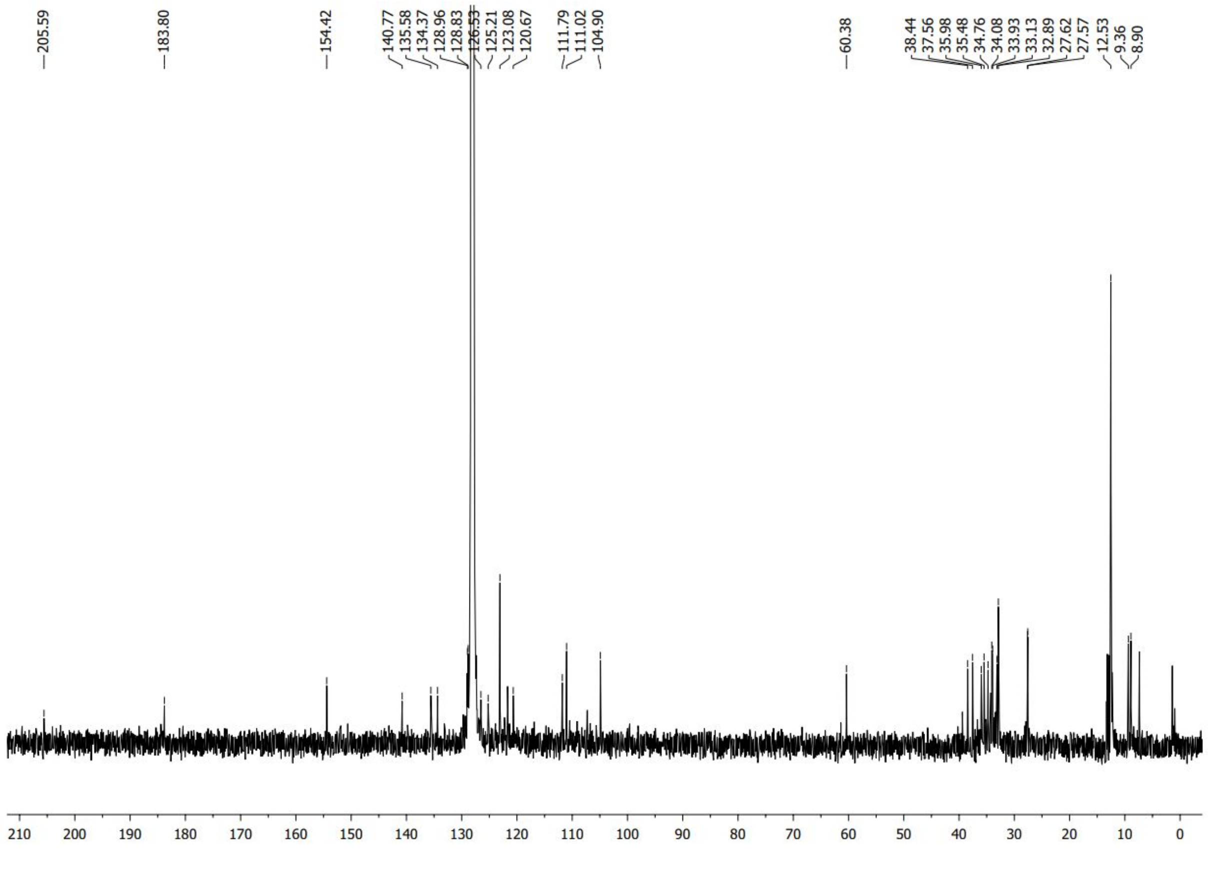

Figure S11: ^{13}C NMR spectrum (125 MHz, C_6D_6, 305 K) of **Ti4a**.

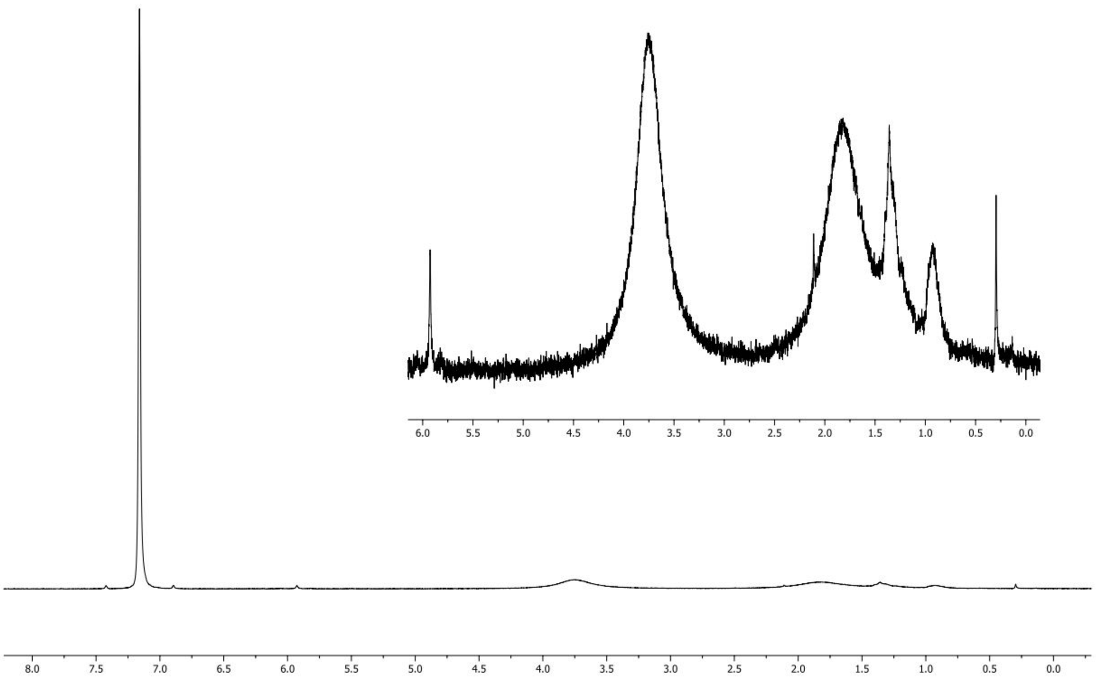

Figure S12: ^{1}H NMR spectrum (300 MHz, C_6D_6, 305 K) of **Ti5a**.

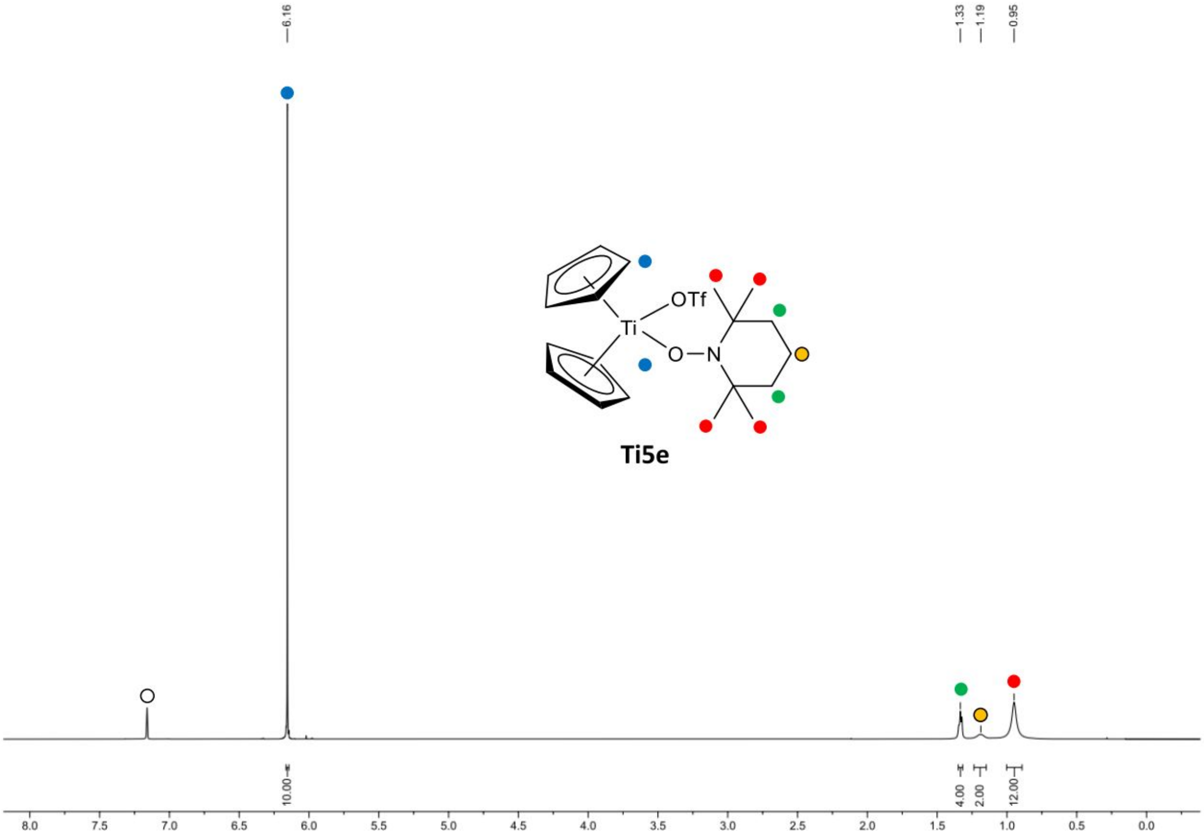

Figure S13: ^{1}H NMR spectrum (500 MHz, C_6D_6, 305 K) of **Ti5e**. Product signals given in colours (° = C_6D_5H).

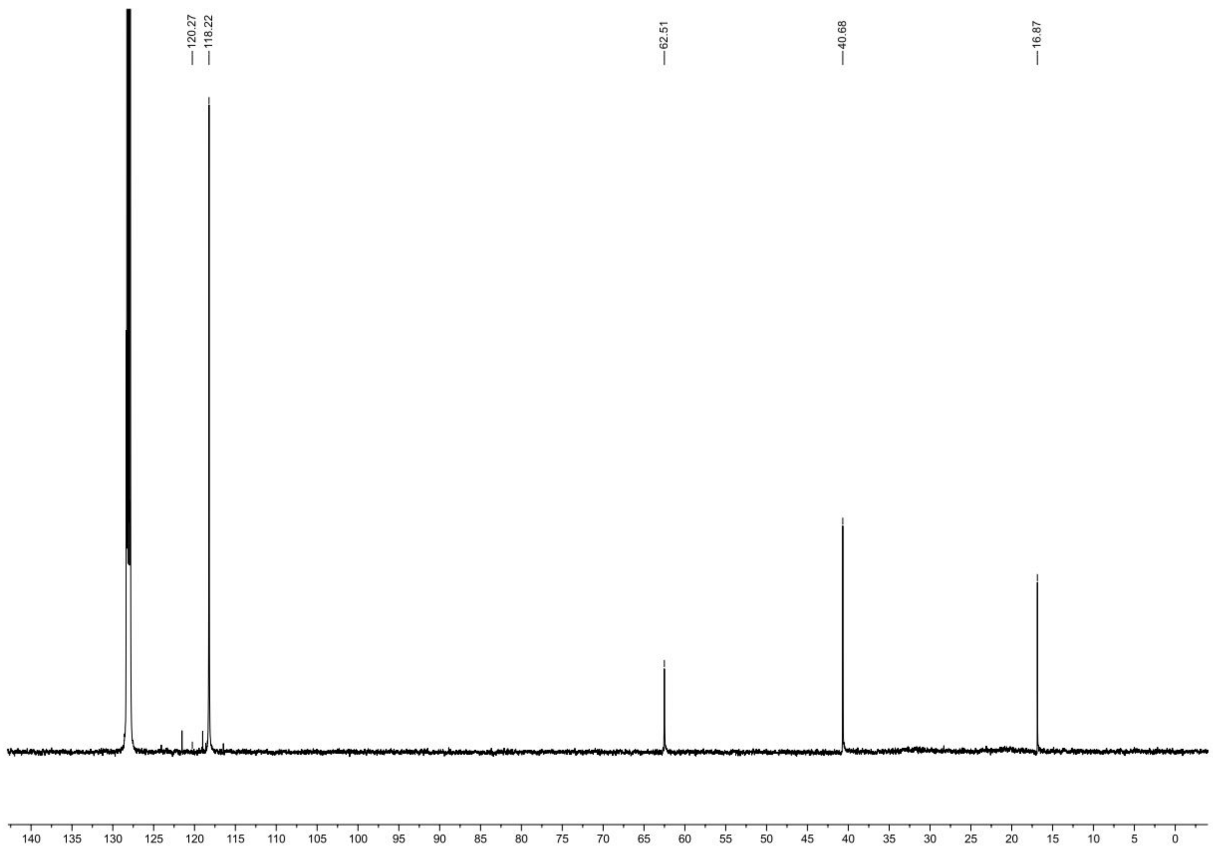

Figure S14: ^{13}C NMR spectrum (125 MHz, C_6D_6, 305 K) of **Ti5e**.

Additional molecular structures

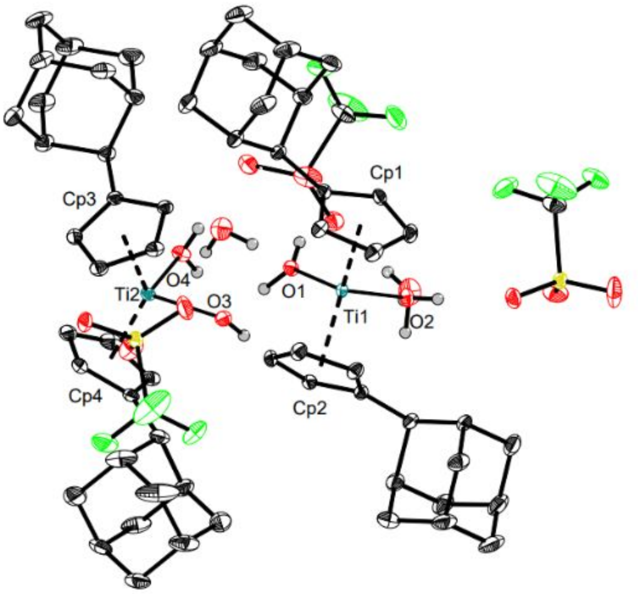

Figure S15: The molecular structure of complex **Ti2a**. Displacement ellipsoids are drawn at the 50% probability level. Abundant H atoms (apart from H_2O H atoms) and solvent molecules have been omitted for clarity. Selected bond lengths (Å) and angles (deg): Ti1–O1 1.9584(17), Ti1–O2 2.0409(15), Ti2–O3 1.9806(17), Ti2–O4 2.0343(16), O3–Ti1–O4 91.999(67) O1–Ti1–O2 90.543(66), Cp1–Ti1–Cp2 132.7, Cp3–Ti1–Cp4 132.5 (Ct1 = centroid of C1–C5; Ct2 = centroid of C16–C20, Ct3 = centroid of C31–C35; Ct4 = centroid of C46–C50).

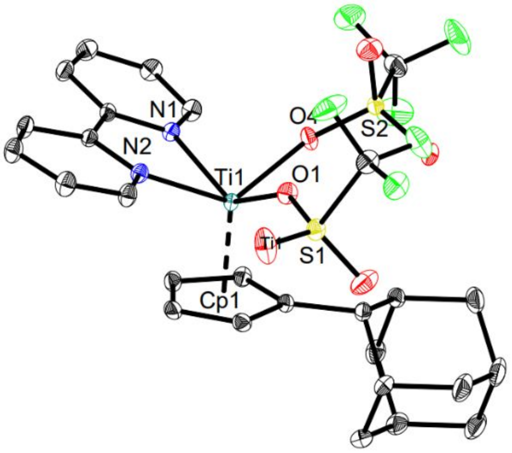

Figure S16: The molecular structure of complex **Ti1a,bipy**. Displacement ellipsoids are drawn at the 50% probability level. Abundant H atoms and solvent molecules have been omitted for clarity. Selected bond lengths (Å) and angles (deg): Ti1–O1 2.0871, Ti1–O4 2.846, Ti1–N1 2.1764, Ti1–N2 2.1773, O1–Ti1–O4 82.82(1), N1–Ti1–N2 74.228(1), O1–Ti1–N2 81.257(1), O4–Ti1–N1 82.423(1).

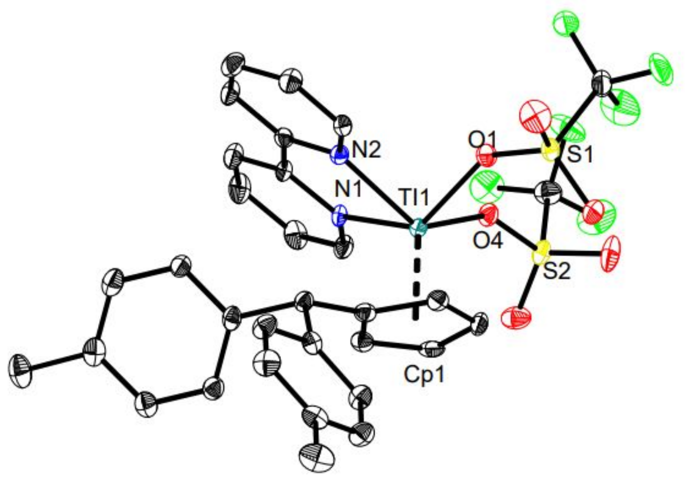

Figure S17: The molecular structure of complex **Ti1b,bipy**. Displacement ellipsoids are drawn at the 50% probability level. Abundant H atoms and solvent molecules have been omitted for clarity. Selected bond lengths (Å) and angles (deg): Ti1–O1 2.10000(1), Ti1–O4 2.0665(1), Ti1–N1 2.1896(1), Ti1–N2 2.2011(1), O1–Ti1–O4 80.828(2), O1–Ti1–N2 86.917(1), O1–Ti1–N1 80.577(2), N1–Ti1–N2 73.516(1).

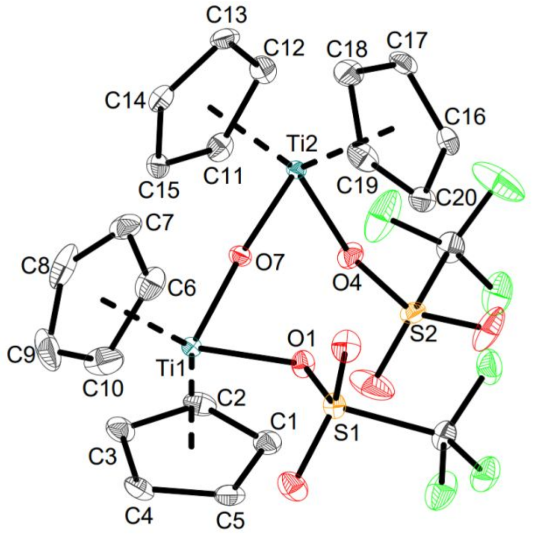

Figure S18: The molecular structure of complex **Ti5oxo**. Displacement ellipsoids are drawn at the 50% probability level. Abundant H atoms and solvent molecules have been omitted for clarity. Selected bond lengths (Å) and angles (deg): Ti1–O1 2.0846(8), Ti2–O4 2.0767(8), Ti1–O7 1.8276(8), Ti2–O7 1.8384(8), O1–Ti1–O7 91.56(3), O4–Ti2–O7 93.10(4), Ti1–O1–Ti2 175.54(5), Ct1–Ti1–Ct2 132.2, Ct3–Ti2–Ct4 131.9 (Ct1 = centroid of C1–C5; Ct2 = centroid of C6–C10, Ct3 = centroid of C11–C15; Ct4 = centroid of C16–C20).

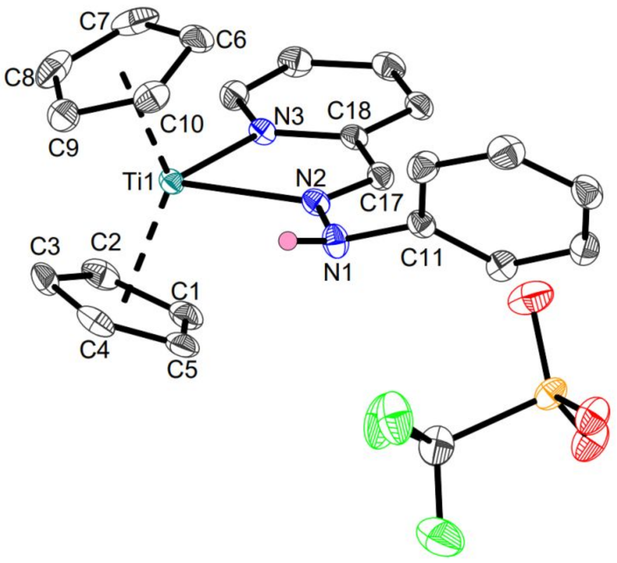

Figure S19: The molecular structure of complex **Ti5x**. Displacement ellipsoids are drawn at the 50% probability level. Abundant H atoms (apart from N1–H) have been omitted for clarity. Selected bond lengths (Å) and angles (deg): Ti1–N2 2.2353(12), Ti1–N3 2.2009(12), N1–N2 1.3773(16), N1–C11 1.4321(18), N2–C17 1.2896(18), N3–C18 1.3562(18), C17–C18 1.4510(19), N2–Ti1–N3 73.24(4), Ti1–N2–N1 122.18(9), Ct1–Ti1–Ct2 132.2, Ct3–Ti2–Ct4 136.0 (Ct1 = centroid of C1–C5; Ct2 = centroid of C6–C10).

Supramolecular features

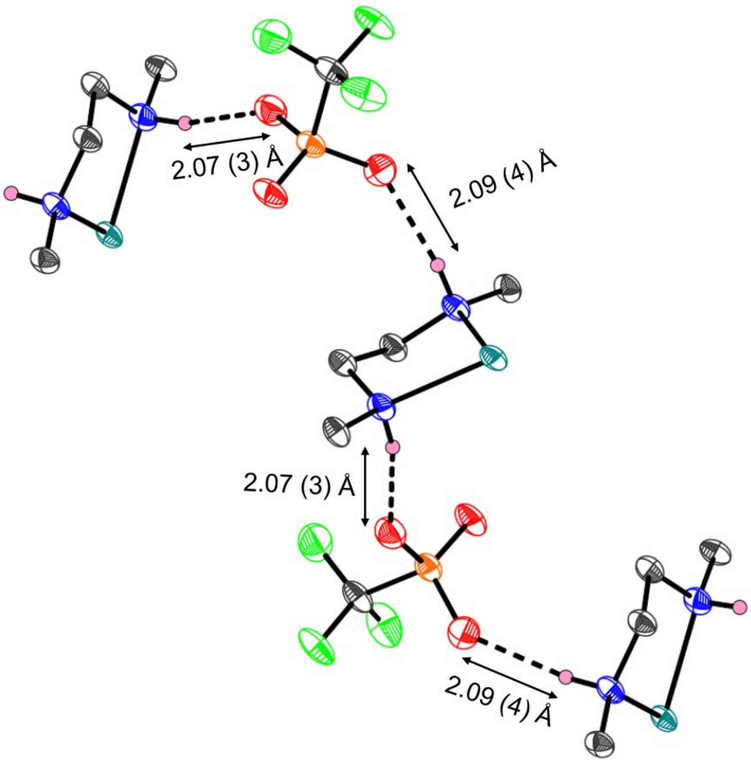

Figure S20: NH···O Hydrogen bonding in **Ti5h**.

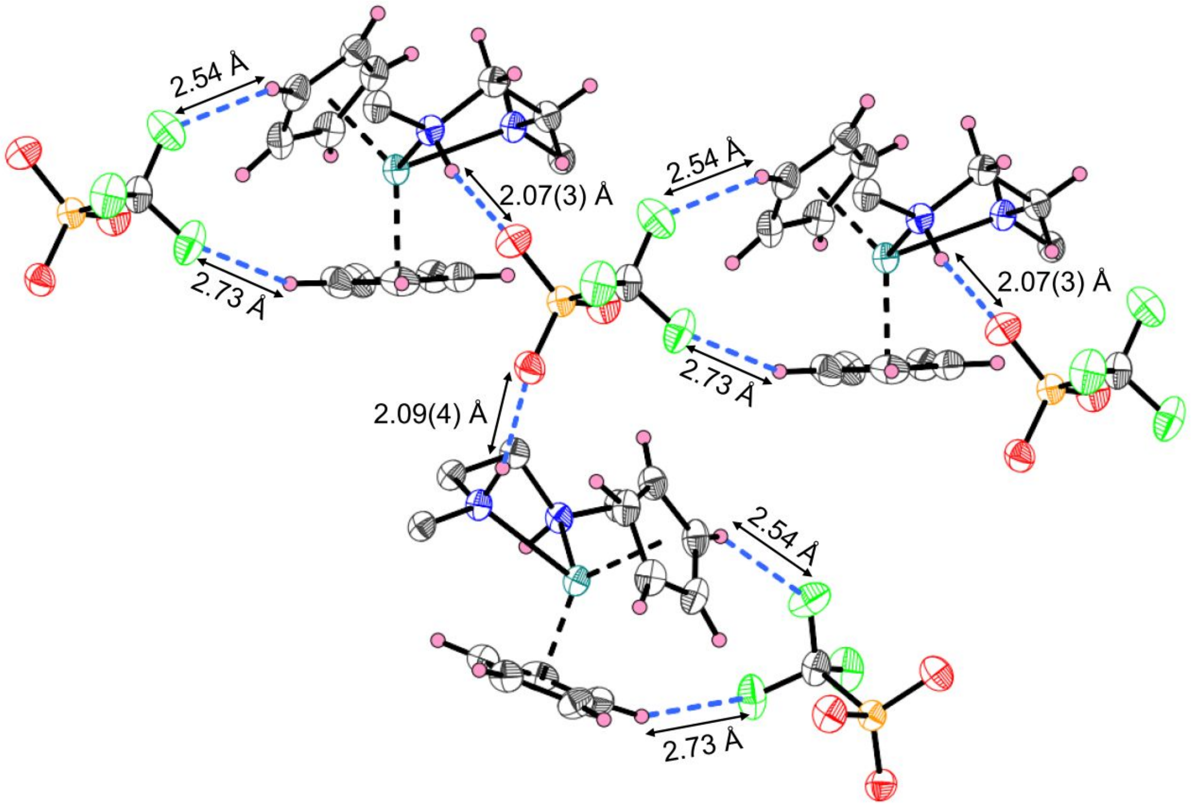

Figure S21: Intermolecular connectivity of **Ti5h** including NH···O Hydrogen bonding and CH···F bonding.

Crystallographic data

Single crystal X-ray data for **Ti5c** and **Ti5oxo** were measured on a Bruker AXS Apex II diffractometer (graphite monochromator, Mo-Kα radiation, λ = 0.71073 Å, Kappa 4-circle goniometer, Apex II CCD detector). All other data were measured on a Bruker AXS D8 Venture diffractometer (multilayer optics, Mo-Kα and Cu-Kα radiation with λ = 0.71073 Å and 1.54178 Å respectively, Kappa 4-circle goniometer, Photon III C14 CPAD detector). The crystal of **Ti5e** was measured at a temperature of 150 K; the crystals crack below 150 K. All other crystals were measured at a temperature of 100 K. Absorption corrections using equivalent reflections were performed with the program SADABS.[1] For **Ti5c** and **Ti5oxo** numerical absorption corrections were performed using the same program. The crystals of **Ti5** and **Ti5d** were non-merohedral twins, and the absorption corrections were performed with the program TWINABS.[2] All structures were solved with the program SHELXS[3] and refined with SHELXL[4] using the OLEX2[5] GUI.

All non H atoms were refined using anisotropic atomic displacement parameters (ADPs). H atoms bonded to C were located in the difference Fourier maps and placed on idealized geometric positions with idealized atomic displacement parameters using the riding model. H atoms bonded to N, O and P were refined freely.

The crystals of **Ti5b** tested undergo a slow phase transition at low temperature, resulting in a poor diffraction pattern, which is (partially) reversible at room temperature. For this reason a quick 2 h data collection was performed on the crystal at 100 K to yield the current dataset.

The structure of **Ti5** exhibits whole molecule disorder. The disordered sites were refined using restraints on the anisotropic ADPs (RIGU instruction within SHELXL). In **Ti5e** the Cp and TEMPO ligands are disordered with refined site occupancies of 0.887 : 0.113. The disordered sites were refined using restraints on the geometry (SAME). In **Ti5g** the triflate anions are disordered with refined site occupancies of 0.820 : 0.180 and 0.874 : 0.126 respectively. The disordered sites were refined using restraints on the geometry (SAME), and the isotropic ADPs were constrained to be the same (EADP).

The crystallographic data can be obtained free of charge from https://www.ccdc.cam.ac.uk/structures/ quoting the CCDC numbers 2334639-2334656.

	**Ti1a**	**Ti2b**	**Ti3a**	**Ti4a**	**Ti5**
CCDC	2334656	2334654	2334652	2334649	2334639
Lab-ID	mazyx	mazy90	mazy130	mazy159	kesc155
empirical formula	$C_{32}H_{38}F_6O_6S_2Ti$	$C_{42}H_{46}F_6O_{10}S_2Ti$ * 0.5 C_6D_6	$C_{33}H_{37}ClF_3NO_3STi$	$C_{40}H_{49}ClF_3N_3O_3STi$ * C_6D_6	$C_{22}H_{20}F_6O_6S_2Ti_2$
Fw	744.64	978.88	668.04	876.37	654.30
Colour	red	yellow orange	red	orange	Green
Habit	block	block	block	plate	Plate
cryst. dimens. mm	0.07 x 0.06 x 0.04	0.14 x 0.13 x 0.09	0.14 x 0.13 x 0.10	0.10 x 0.06 x 0.025	0.10 x 0.07 x 0.03
cryst. system	monoclinic	triclinic	monoclinic	monoclinic	Monoclinic
space group	$P2_1/n$	P-1	$P2_1/n$	$P2_1/c$	$P2_1/n$
a, Å	13.8757(6)	11.9360(10)	11.0115(4)	10.5135(5)	8.2986(5)
b, Å	16.7951(6)	14.1413(12)	24.5461(8)	16.8880(9)	8.1109(4)
c, Å	27.1401(11)	14.1897(11)	11.9377(4)	24.3029(13)	19.0689(10)
α, deg	90	99.889(3)	90	90	90
β, deg	92.5781(16)	91.659(3)	108.4242(13)	98.6672(18)	97.985(2)
γ, deg	90	101.691(3)	90	90	90
V, Å^3	6318.4(4)	2305.5(3)	3061.24(18)	4265.8(4)	1271.07(12)
Z	8	2	4	4	2
$D_{calc.}$, g cm^{-3}	1.566	1.410	1.449	1.365	1.710
μ, mm^{-1}	0.484	0.356	0.489	0.370	0.874
T, K	100(2)	100(2)	100(2)	100(2)	100(2)
λ, Å	0.71073	0.71073	0.71073	0.71073	0.71073
θ range, deg	1.426– 33.728	1.460 – 36.318	1.659 – 34.970	1.474 – 28.699	2.157 – 30.067
reflections collected	291583	207151	210604	143085	5767
Indep. Reflecions R(int)	25244<br>0.0747	22366<br>0.0354	13452<br>0.0475	11018<br>0.0730	5767<br>?
Observed reflections (I > 2(I))	17765	20181	12299	9843	5154
Absorption correction	semi-empirical	semi-empirical	semi-empirical	semi-empirical	semi-empirical
max, min transm.	1.0000, 0.9302	1.0000, 0.9431	1.0000, 0.9022	1.0000, 0.9116	1.0000, 0.867742
final R indices [$I>2\sigma(I)$]	R1 = 0.0579, wR2 = 0.1205	R1 = 0.0415, wR2 = 0.1090	R1 = 0.0372, wR2 = 0.0918	R1 = 0.0783, wR2 = 0.1530	R1 = 0.0478, wR2 = 0.0910
R indices (all data)	R1 = 0.0883, wR2 = 0.1335	R1 = 0.0463, wR2 = 0.1122	R1 = 0.0418, wR2 = 0.0944	R1 = 0.0876, wR2 = 0.1563	R1 = 0.0597, wR2 = 0.0950
GOF on F^2	1.080	1.070	1.132	1.267	1.157
largest diff peak / hole (e.Å^{-3})	1.298 / -0.542	0.996 / -0.832	0.816 / -0.391	0.810 / -0.479	0.351 / -0.408

	Ti5b	Ti5c	Ti5d	Ti5e	Ti5f
CCDC	2334644	2334651	2334640	2334645	2334641
Lab-ID	kesc171b	kesc166	kesc164	kesc172	kesc216
empirical formula	$C_{16}H_{15}F_3NO_3STi$	$C_{29}H_{25}F_3O_4PSTi$	$C_{18}H_{14}F_4NO_3STi$	$C_{23}H_{31}F_3NO_4STi$	$C_{17}H_{22}F_3O_5STi$
Fw	406.25	605.42	448.26	522.45	443.30
Colour	green	green	green	yellow green	green yellow
Habit	block	block	block	plate	block
cryst. dimens. mm	0.20 x 0.16 x 0.06	0.40 x 0.40 x 0.30	0.14 x 0.09 x 0.05	0.16 x 0.16 x 0.04	0.14 x 0.08 x 0.03
cryst. system	monoclinic	monoclinic	monoclinic	triclinic	orthorhombic
space group	$P2_1/c$	$P2_1/c$	$P2_1/n$	P-1	$P2_12_12_1$
a, Å	7.8378(4)	10.5965(3)	12.1102(12)	8.0525(7)	8.1799(3)
b, Å	8.0458(4)	18.2443(6)	8.4665(8)	9.4180(8)	9.0486(3)
c, Å	26.3260(14)	14.2169(5)	17.5108(18)	16.5961(14)	27.0247(10) Å
α, deg	90	90	90	92.752(3)	90
β, deg	94.507(2)	105.7651(12)	96.209(4)	95.565(3))	90
γ, deg	90	90	90	95.954(3)	90
V, Å^3	1655.02(15)	2645.10(15)	1784.9(3)	1243.79(18)	2000.28(12)
Z	4	4	4	2	4
$D_{calc.}$, g cm^{-3}	1.630	1.520	1.668	1.395	1.472
μ, mm^{-1}	0.690	0.520	0.657	0.479	0.584
T, K	100(2)	100(2)	100(2)	150(2)	100(2)
λ, Å	0.71073	0.71073	1.54178	0.71073	0.71073
θ range, deg	1.552 – 30.033	1.860 – 40.248	1.950 – 36.318	2.178 – 34.971	1.507 – 32.031
reflections collected	29926	174547	16836	86950	31040
Indep. Reflecions R(int)	4828 0.0497	16646 0.0280	16836 ?	10905 0.0458	6973 0.0512
Observed reflections (I > 2(I))	4311	14131	15730	9820	6445
Absorption correction	semi-empirical	numerical	semi-empirical	semi-empirical	semi-empirical
max, min transm.	1.0000, 0.9152	0.8984, 0.8356	1.000000, 0.922780	1.0000, 0.9006	1.0000, 0.7572
final R indices [$I>2\sigma(I)$]	R1 = 0.0505, wR2 = 0.1139	R1 = 0.0341, wR2 = 0.0893	R1 = 0.0365, wR2 = 0.0844	R1 = 0.0437, wR2 = 0.1122	R1 = 0.0454, wR2 = 0.1148
R indices (all data)	R1 = 0.0574, wR2 = 0.1165	R1 = 0.0432, wR2 = 0.0948	R1 = 0.0402, wR2 = 0.0864	R1 = 0.0487, wR2 = 0.1151	R1 = 0.0503, wR2 = 0.1177
GOF on F^2	1.143	1.046	1.121	1.088	1.113
largest diff peak / hole (e.Å^{-3})	1.006 / -0.523	1.208 / -0.971	0.679 / -0.365	0.717 / -0.472	1.240 / -0.280

	**Ti5g**	**Ti5h**	**Ti5i**	**Ti2a**	**Ti1a,bipy**
CCDC	2334646	2334642	2334647	2334650	2334655
Lab-ID	kesc190	kesc245	kesc233	mazy89	mazy65
empirical formula	$C_{23}H_{22}F_3FeO_4STi$	$C_{25}H_{26}F_3N_2O_3STi$	$C_{33}H_{30}F_3FeO_3P_2STi$	$C_{63}H_{87}F_9O_{15}S_3Ti_2$	$C_{34}H_{35}F_6N_2O_6S_2Ti$
Fw	555.21	39.44	729.32	1447.30	793.66
Colour	red	pale green	yellow brown	yellow green	green
Habit	plate	needle	block	plate	block
cryst. dimens. mm	0.12 x 0.06 x 0.03	0.12 x 0.02 x 0.01	0.09 x 0.07 x 0.05	0.13 x 0.10 x 0.04	0.12 x 0.09 x 0.08
cryst. system	triclinic	monoclinic	orthorhombic	monoclinic	monoclinic
space group	P-1	$P2_1/n$	$Pna2_1$	Cc	$P2_1/n$
a, Å	11.1914(7)	9.5743(3)	18.2224(4)	11.3086(5)	19.3714(9)
b, Å	13.8998(9)	13.7595(5)	10.4483(3)	28.7802(14)	17.8339(9)
c, Å	14.8856(10)	18.3144(6)	15.7587(4)	20.6772(10)	20.8671(10)
α, deg	78.545(3)	90	90	90	90
β, deg	89.530(3)	99.193(2)	90	101.294(2)	105.6382(17)
γ, deg	84.040(3)	90	90	90	90
V, $Å^3$	2257.0(3)	2381.70(14)	3000.35(13)	6599.3(5)	6942.1(6)
Z	4	4	4	4	8
$D_{calc.}$, g cm^{-3}	1.634	1.504	1.615	1.457	1.519
μ, mm^{-1}	1.142	4.348	0.980	0.427	0.447
T, K	100(2)	100(2)	100(2)	100(2)	100(2)
λ, Å	0.71073	1.54178	0.71073	0.71073	0.71073
θ range, deg	1.396 – 28.700	4.037 – 74.493	2.235 – 30.034	1.415 – 34.971	1.274 – 36.319
reflections collected	87140	35968	88797	149904	446519
Indep. Reflecions R(int)	11649<br>0.0458	4832<br>0.0971	8776<br>0.0470	28952<br>0.0446	33656<br>0.0435
Observed reflections (I > 2(I))	9040	3714	8416	27115	29834
Absorption correction	semi-empirical	semi-empirical	semi-empirical	semi-empirical	semi-empirical
max, min transm.	1.0000, 0.8422	1.0000, 0.8010	1.0000, 0.9225	1.0000, 0.9271	1.0000, 0.9521
final R indices [$I>2\sigma(I)$]	R1 = 0.0436, wR2 = 0.1106	R1 = 0.0548, wR2 = 0.1389	R1 = 0.0354, wR2 = 0.0882	R1 = 0.0403, wR2 = 0.0951	R1 = 0.0406, wR2 = 0.0937
R indices (all data)	R1 = 0.0603, wR2 = 0.1217	R1 = 0.0744, wR2 = 0.1534	R1 = 0.0377, wR2 = 0.0896	R1 = 0.0444, wR2 = 0.0975	R1 = 0.0475, wR2 = 0.0969
GOF on F^2	1.049	1.027	1.071	1.052	1.092
largest diff peak / hole (e.Å- ³)	0.689 / -0.837	1.098 / -0.529	1.268 / -0.409	0.756 / -0.585	0.846 / -0.575

	**Ti1b,bipy**	**Ti5oxo**	**Ti5x**
CCDC	2334653	2334648	2334643
Lab-ID	mazyx2	kesc175	kesc142
empirical formula	$C_{47}H_{27}D_{15}F_6N_2O_6S_2Ti$	$C_{22}H_{20}F_6O_7S_2Ti_2$	$C_{23}H_{21}F_3N_3O_3STi$
Fw	971.94	670.30	524.39
Colour	pink green	orange	pale pink
Habit	plate	block	plate
cryst. dimens. mm	0.12 x 0.06 x 0.02	0.35 x 0.35 x 0.25	0.07 x 0.07 x 0.015
cryst. system	monoclinic	orthorhombic	monoclinic
space group	$P2_1/n$	Iba2	$P2_1/n$
a, Å	25.2194(10)	15.7811(8)	10.1104(3)
b, Å	10.3730(4)	16.1295(8)	13.8556(3)
c, Å	33.5019(13)	20.1995(10)	16.4309(4)
α, deg	90	90	90
β, deg	91.4604(15)	90	99.3201(12)
γ, deg	90	90	90
V, $Å^3$	8761.3(6)	5141.6(4)	2271.35(10)
Z	8	8	4
$D_{calc.}$, g cm^{-3}	1.474	1.732	1.533
μ, mm^{-1}	0.368	0.869	4.557
T, K	100(2)	100(2)	100(2)
λ, Å	0.71073	0.71073	1.54178
θ range, deg	1.615 – 28.699	1.805 – 40.247	4.197 – 74.495
reflections collected	235493	122221	39222
Indep. Reflecions R(int)	22595<br>0.0684	15998<br>0.0273	4646<br>0.0368
Observed reflections (I > 2(I))	18931	15300	4315
Absorption correction	semi-empirical	numerical	semi-empirical
max, min transm.	1.0000, 0.9230	0.8573, 0.7537	1.0000, 0.8394
final R indices [$I>2\sigma(I)$]	R1 = 0.0542, wR2 = 0.1037	R1 = 0.0206, wR2 = 0.0523	R1 = 0.0281, wR2 = 0.0725
R indices (all data)	R1 = 0.0689, wR2 = 0.1091	R1 = 0.0227, wR2 = 0.0534	R1 = 0.0308, wR2 = 0.0745
GOF on F^2	1.138	1.038	1.027
largest diff peak / hole (e.$Å^{-3}$)	0.437 / -0.471	0.505 / -0.294	0.528 / -0.470

EPR Spectra

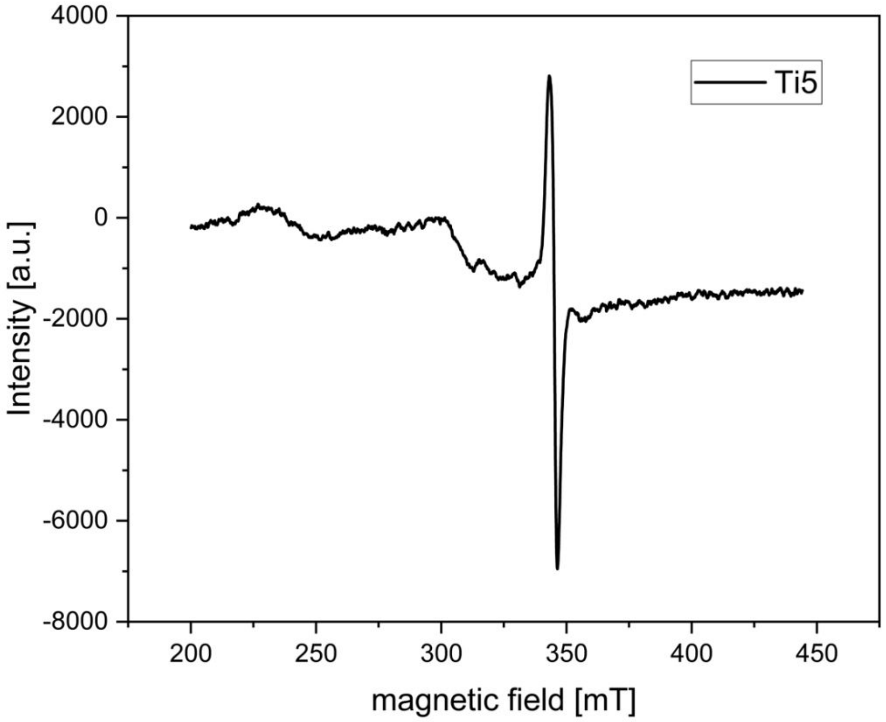

Figure S22: EPR spectrum of complex **Ti5** in toluene at room temperature.

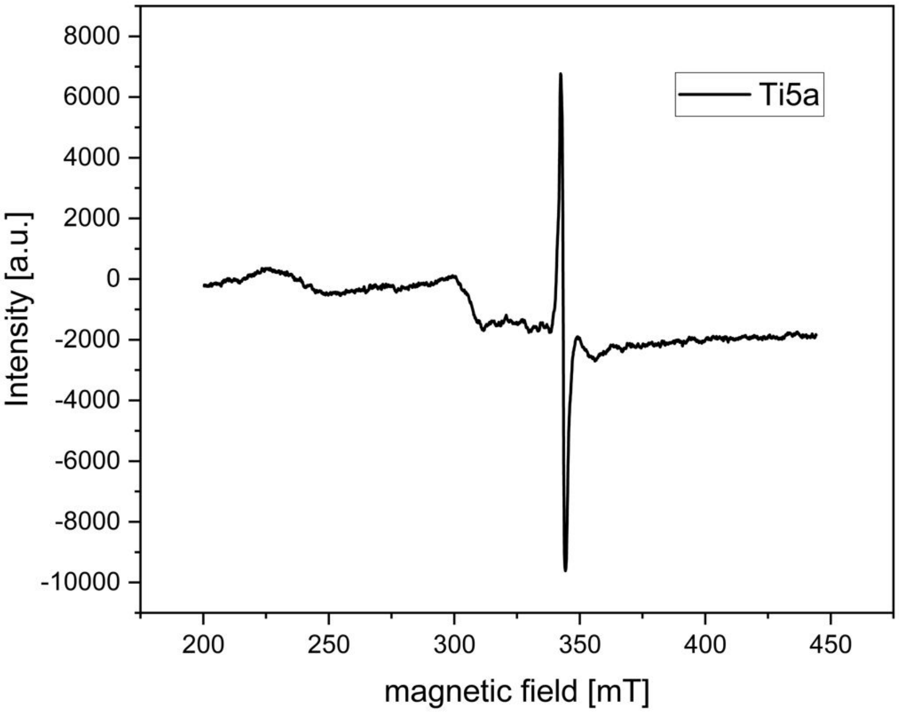

Figure S23: EPR spectrum of complex **Ti5a** in toluene at room temperature.

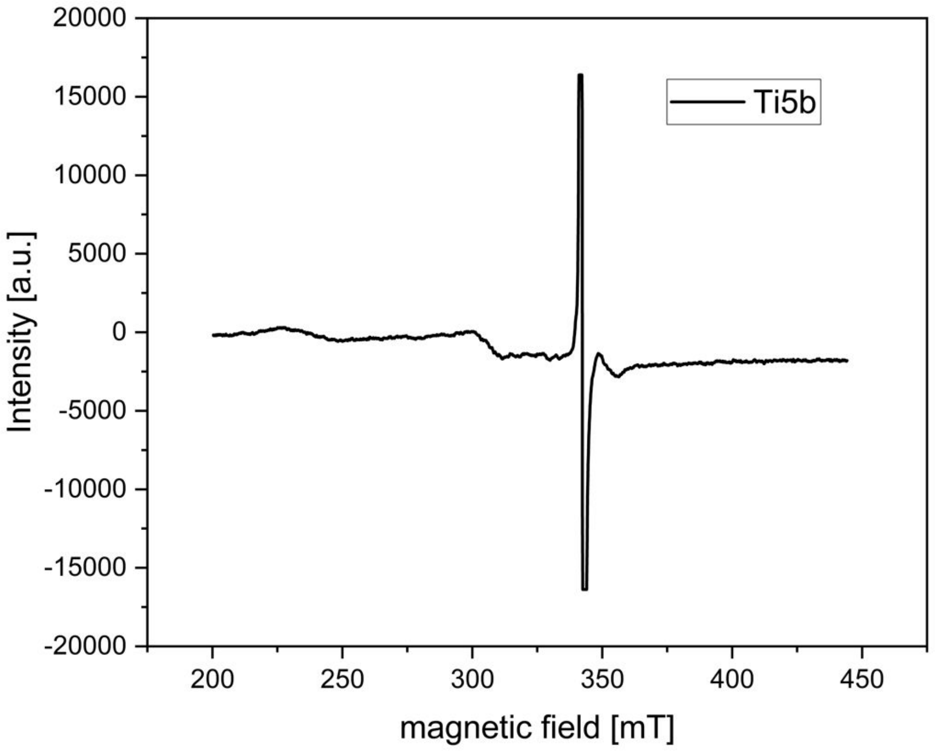

Figure S24: EPR spectrum of complex **Ti5b** in toluene at room temperature.

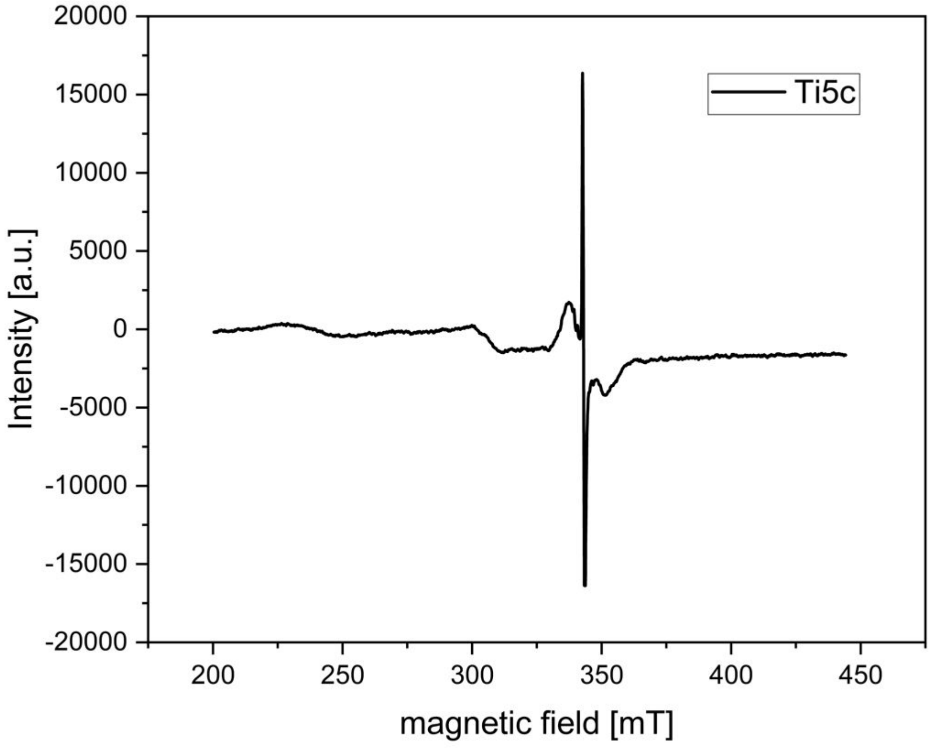

Figure S25: EPR spectrum of complex **Ti5c** in toluene at room temperature.

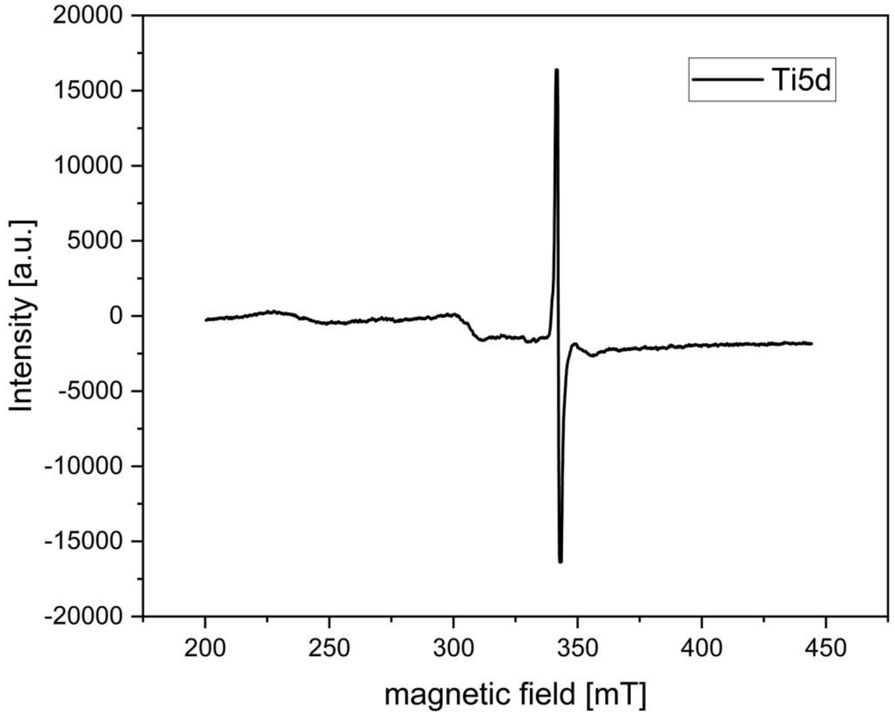

Figure S26: EPR spectrum of complex **Ti5d** in toluene at room temperature.

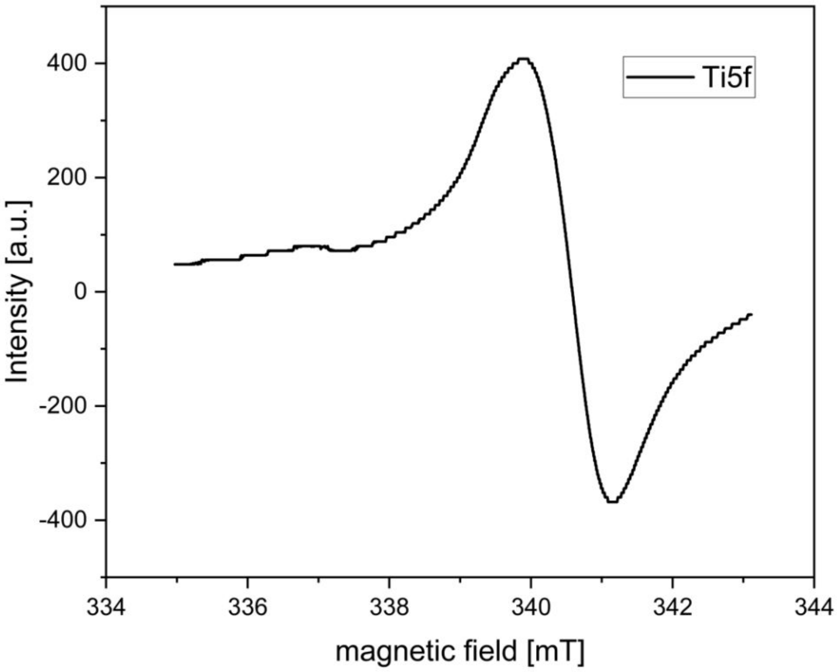

Figure S27: EPR spectrum of complex **Ti5f** in toluene at room temperature.

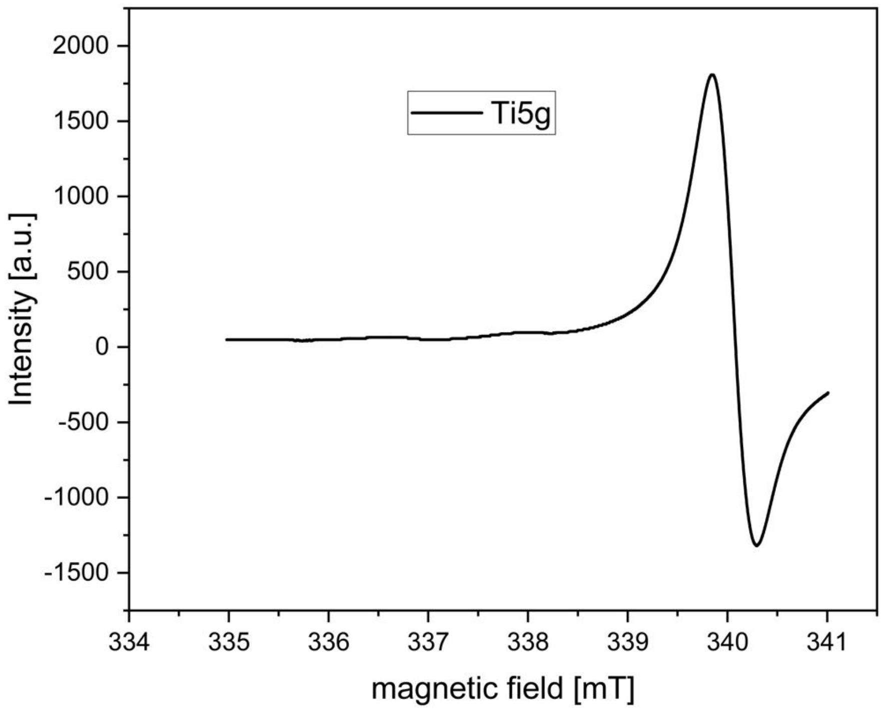

Figure S28: EPR spectrum of complex **Ti5g** in toluene at room temperature.

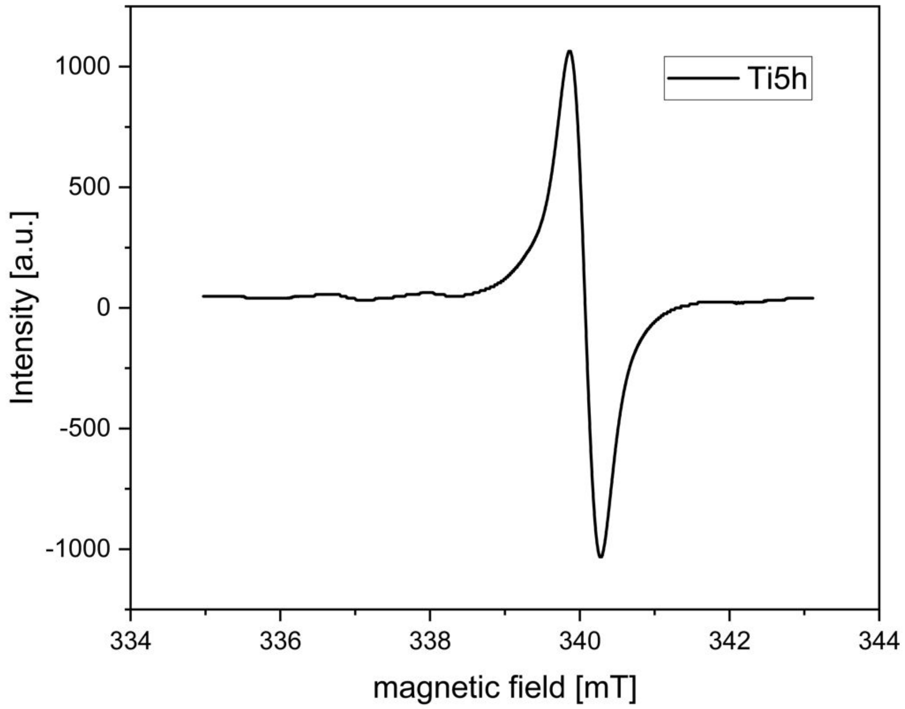

Figure S29: EPR spectrum of complex **Ti5h** in toluene at room temperature.

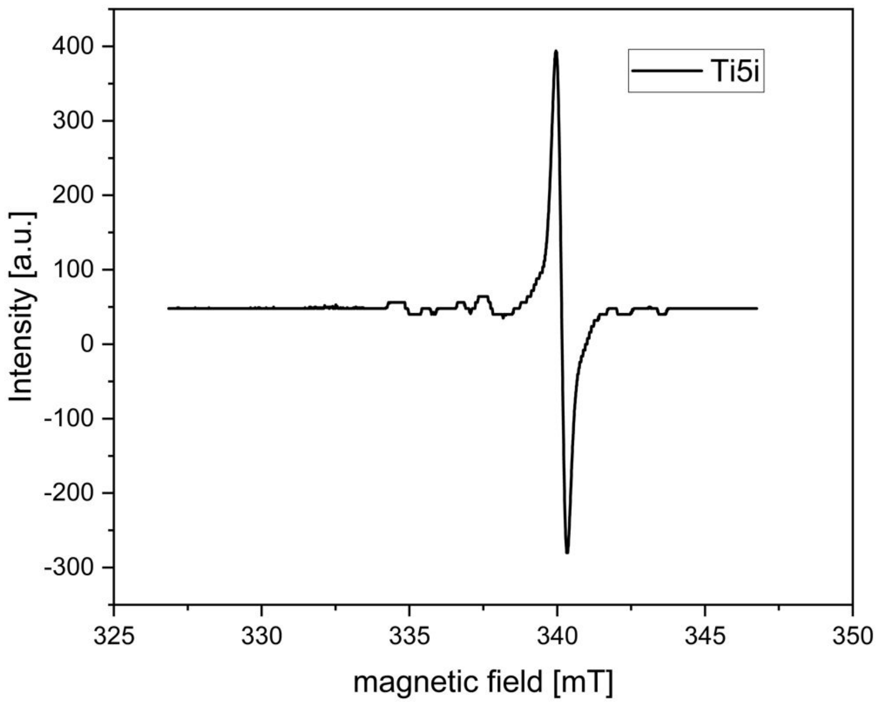

Figure S30: EPR spectrum of complex **Ti5i** in toluene at room temperature.

IR Spectra

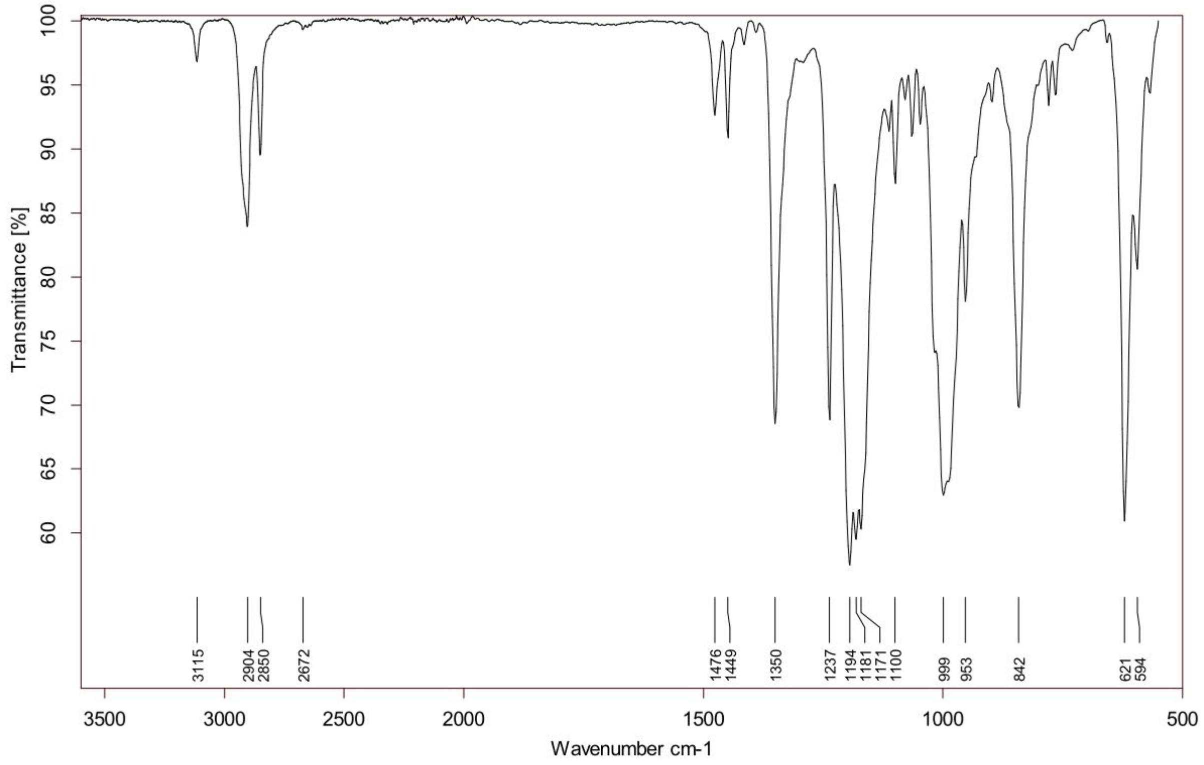

Figure S31: IR Spectrum of **Ti1a**.

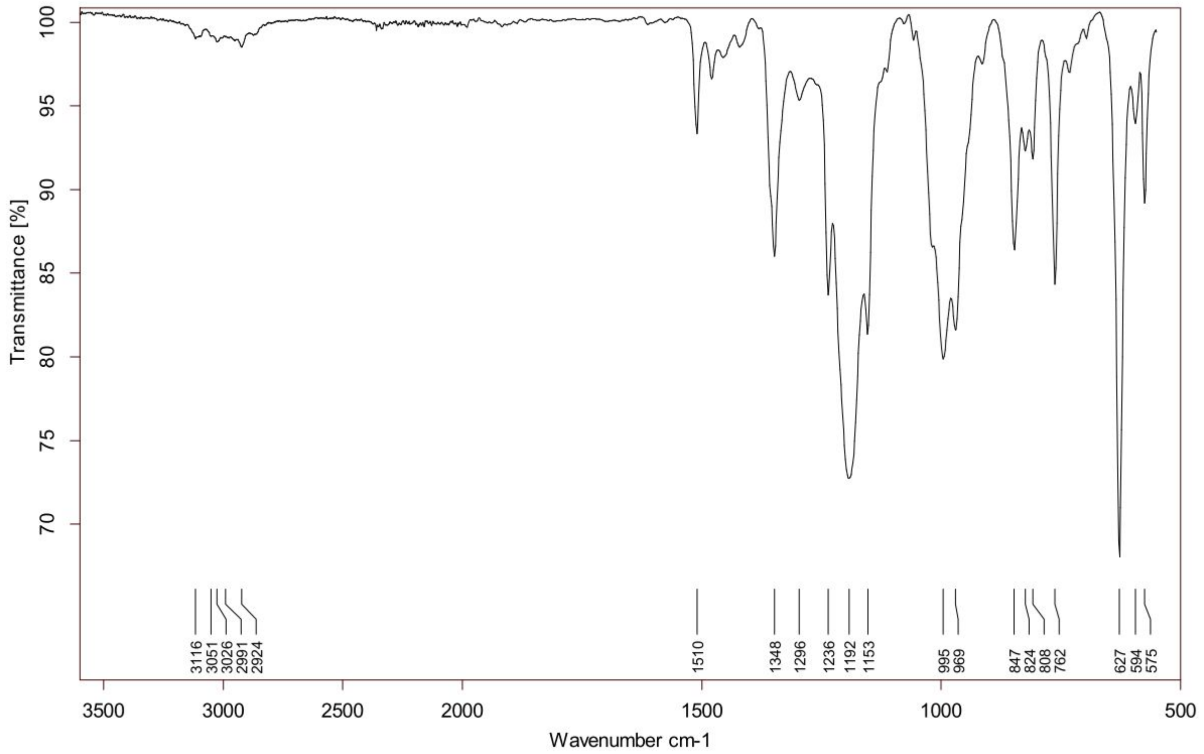

Figure S32: IR Spectrum of **Ti1b**.

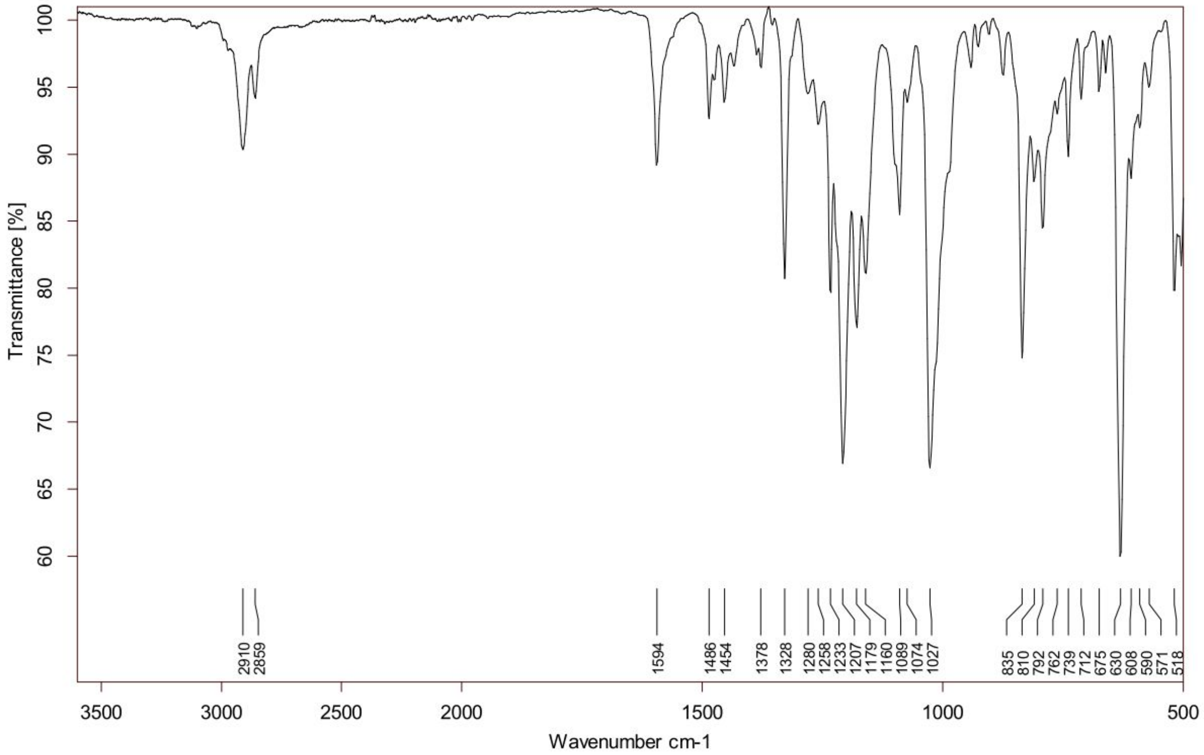

Figure S33: IR Spectrum of **Ti3a**.

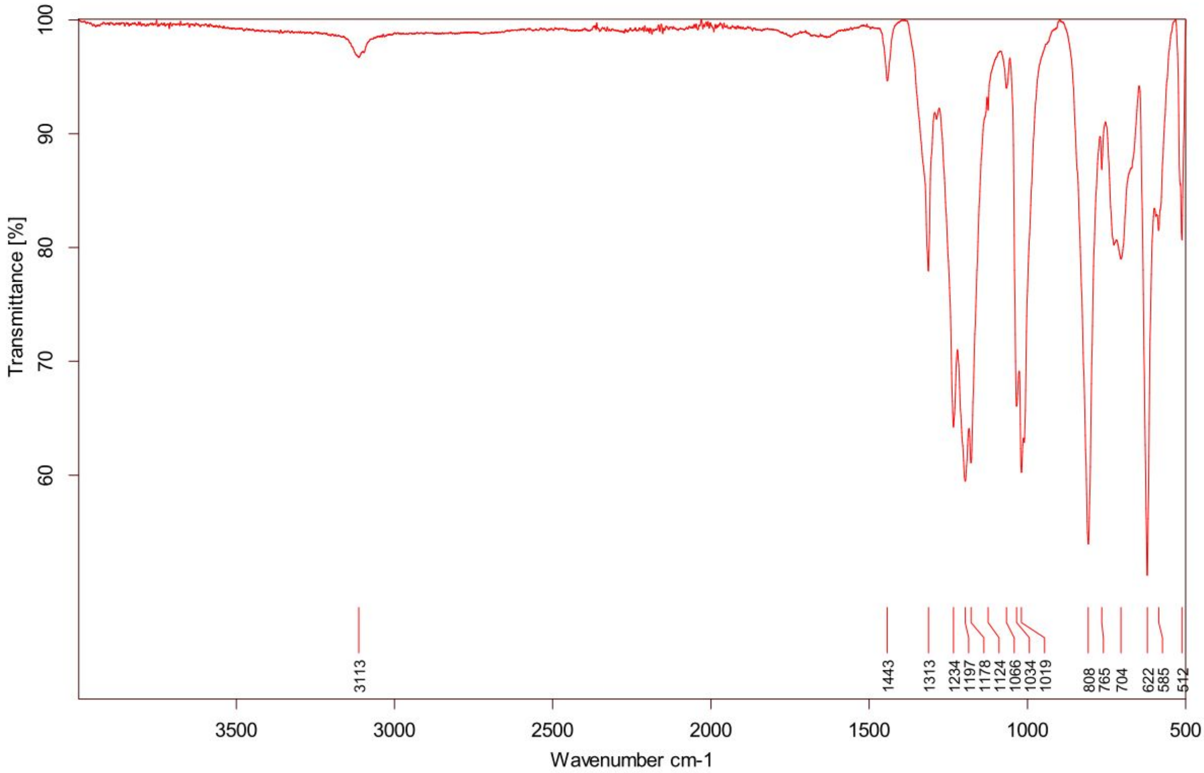

Figure S34: IR Spectrum of **Ti5**.

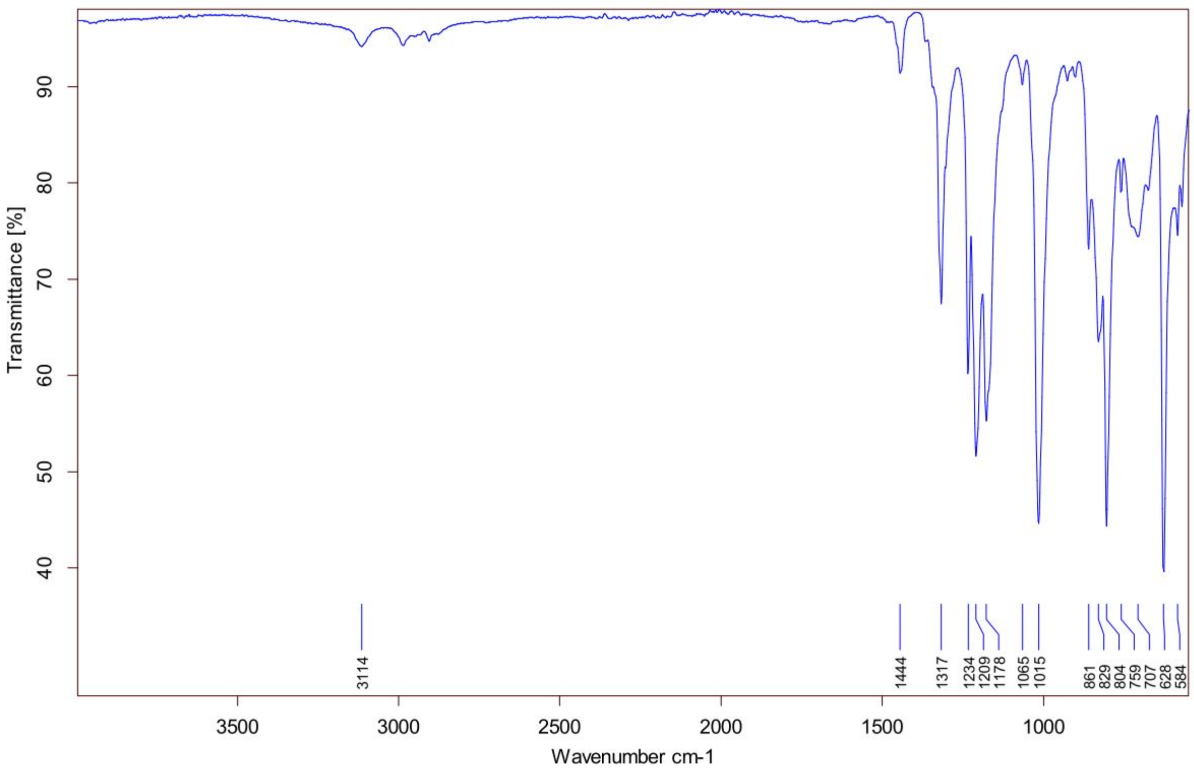

Figure S35: IR Spectrum of **Ti5a**.

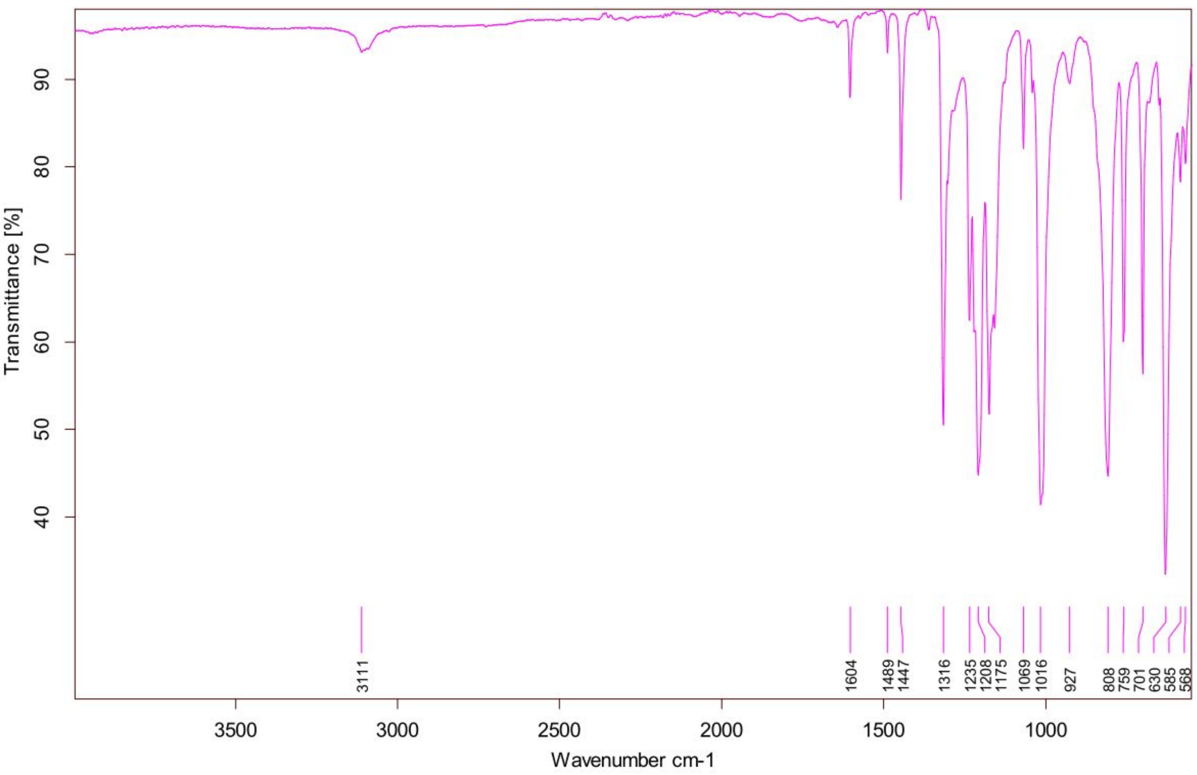

Figure S36: IR Spectrum of **Ti5b**.

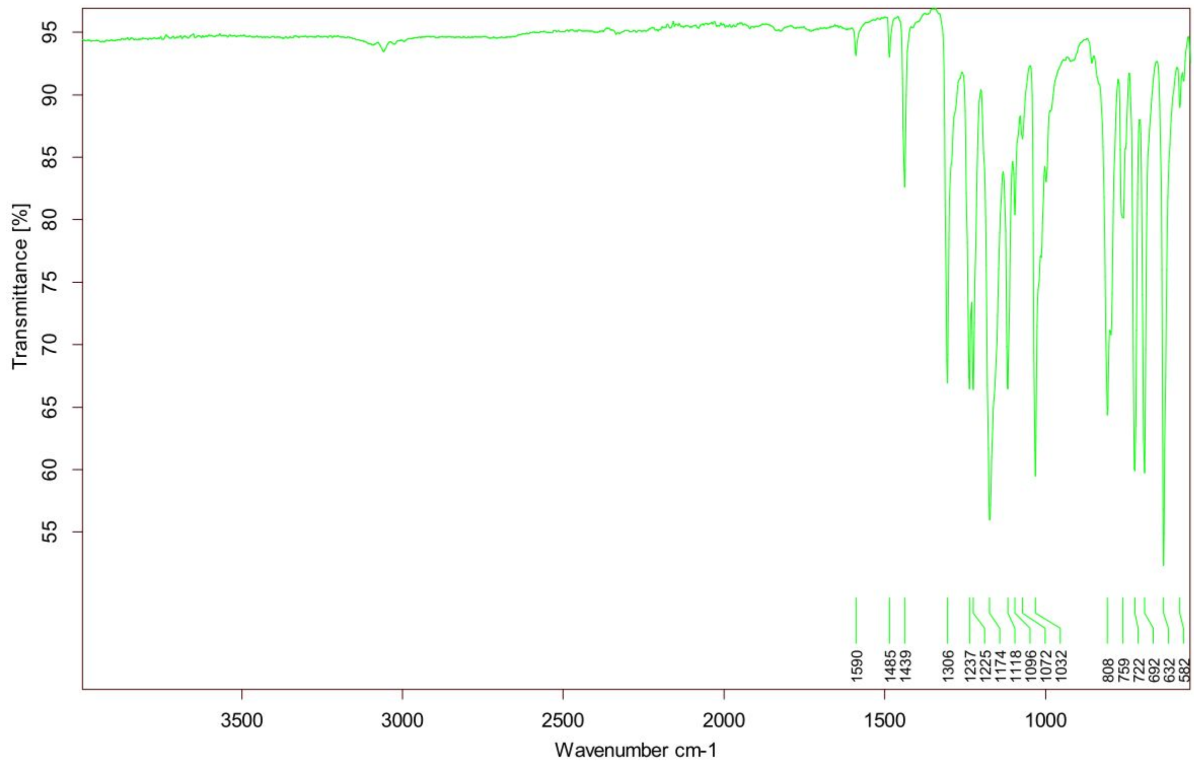

Figure S37: IR Spectrum of **Ti5c**.

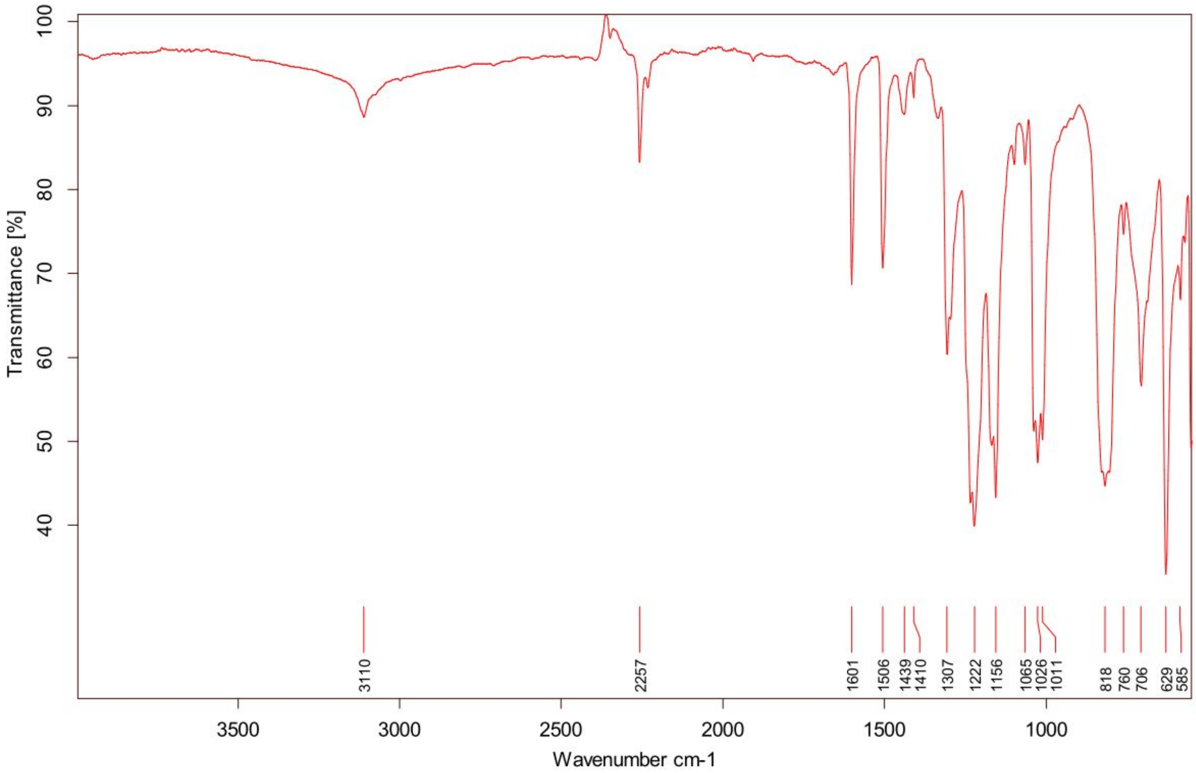

Figure S38: IR Spectrum of **Ti5d**.

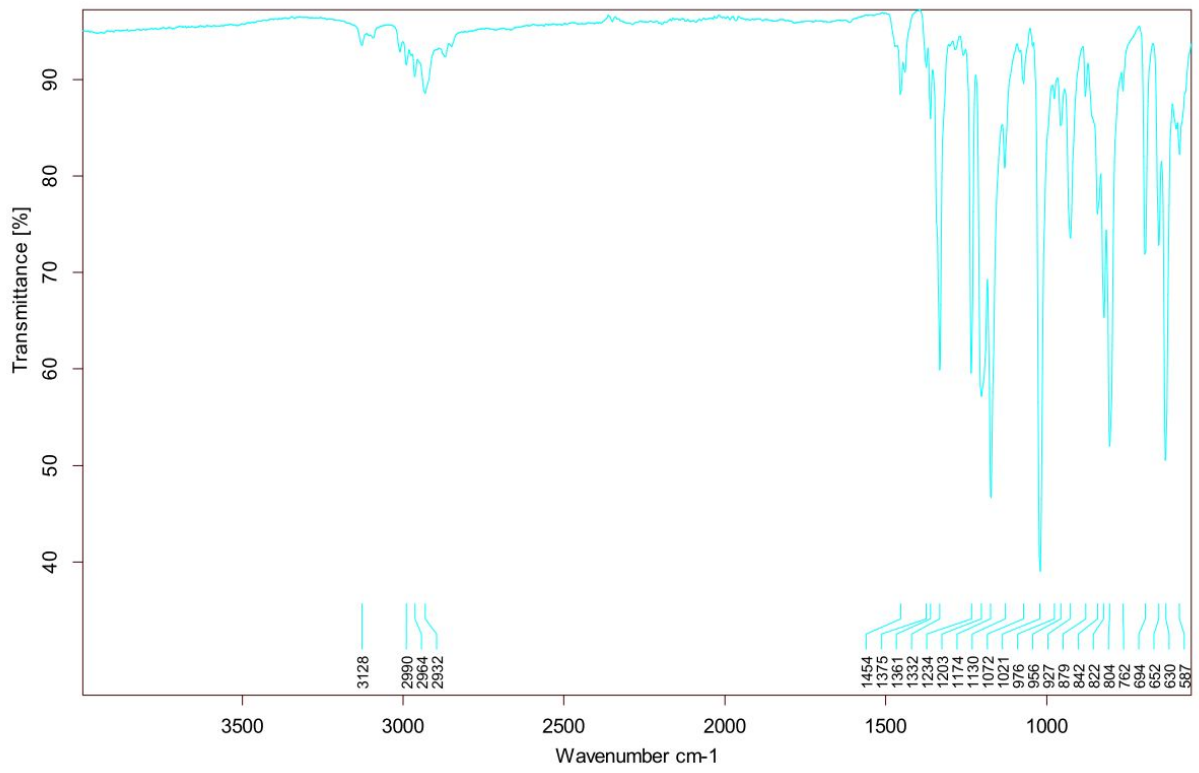

Figure S39: IR Spectrum of **Ti5e**.

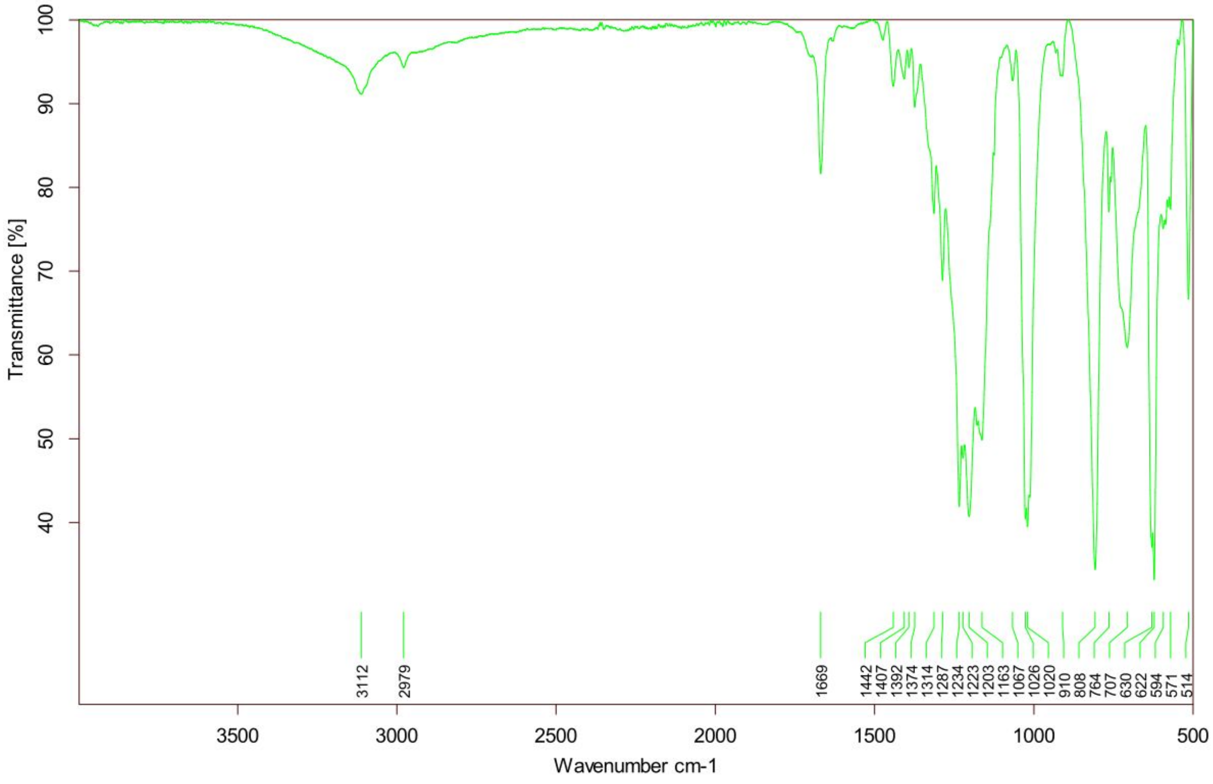

Figure S40: IR Spectrum of **Ti5f**.

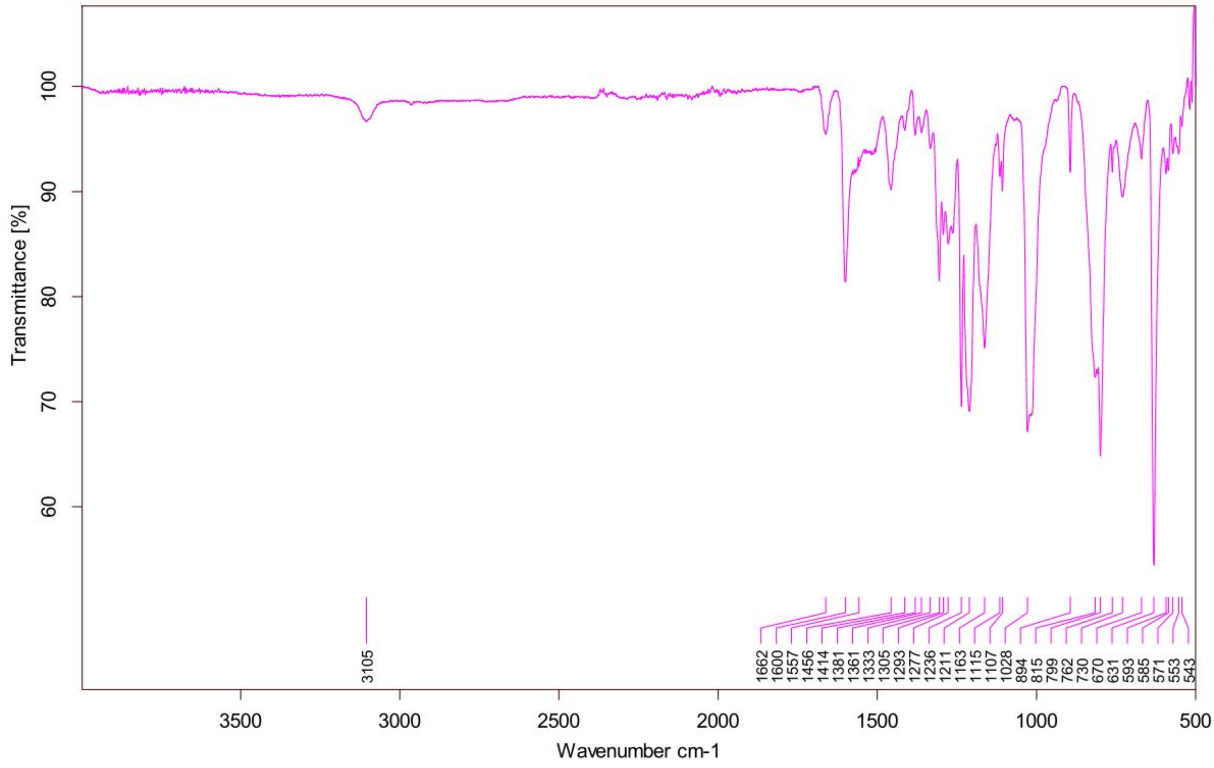

Figure S41: IR Spectrum of **Ti5g**.

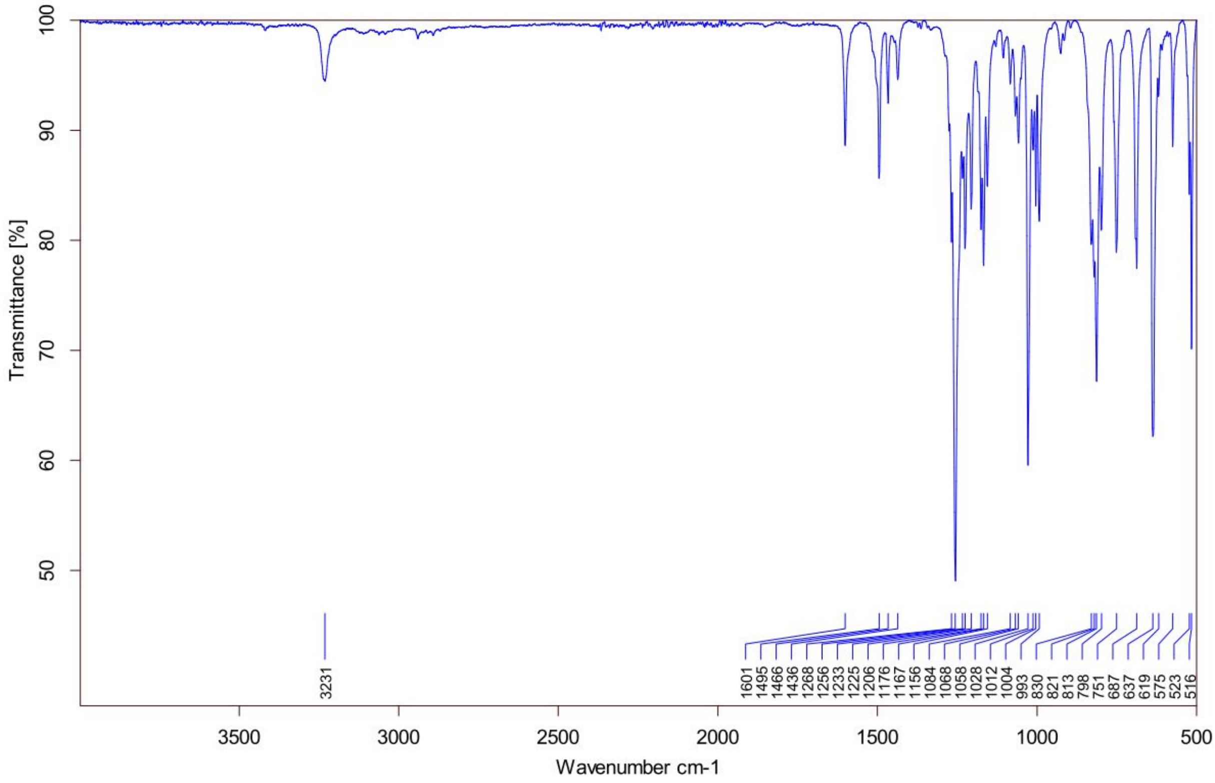

Figure S42: IR Spectrum of **Ti5h**.

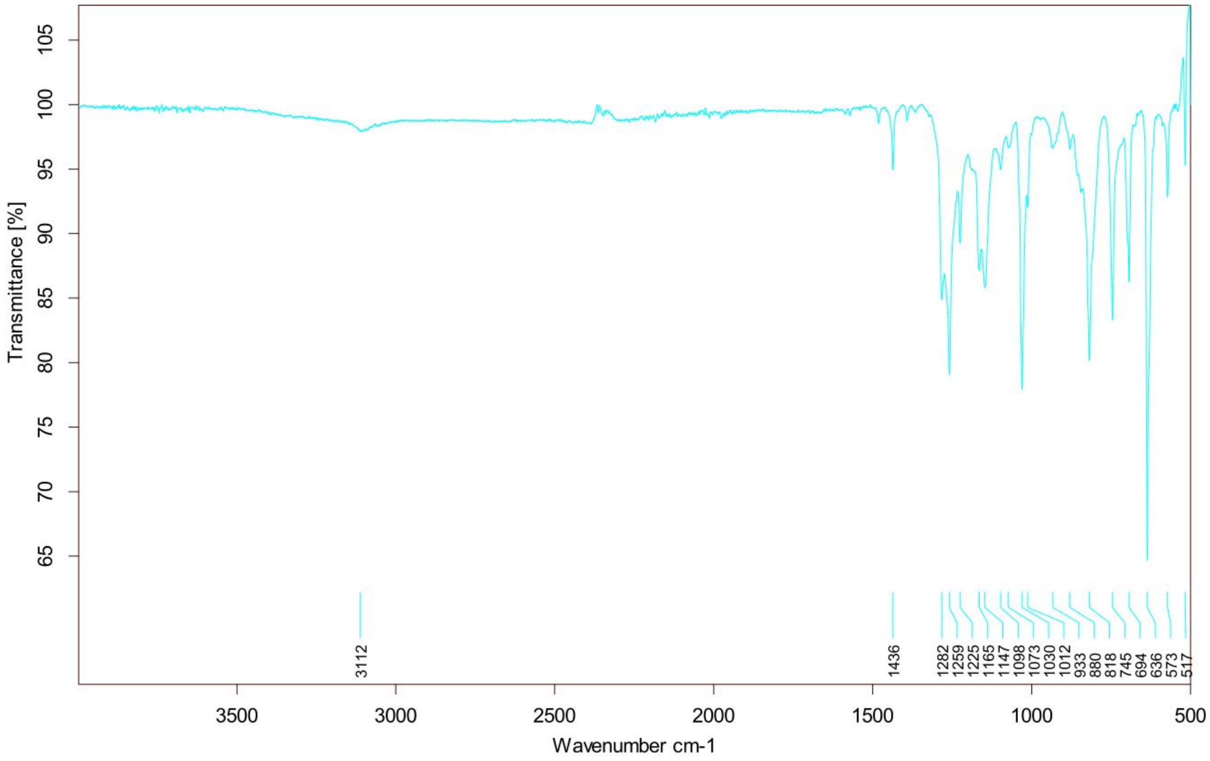

Figure S43: IR Spectrum of **Ti5i**.

References

[1] L. Krause, R. Herbst-Irmer, G. M. Sheldrick, D. Stalke, Comparison of silver and molybdenum microfocus X-ray sources for single-crystal structure determination, *Journal of Applied Crystallography* **2015**, *48*, 3-10.

[2] G. M. Sheldrick, TWINABS 2012/1, Bruker, Madison, Wisconsin, USA, **2012**.

[3] G. M. Sheldrick, A short history of SHELX, *Acta Crystallographica Section A* **2008**, *64*, 112-122.

[4] G. M. Sheldrick, SHELXT – Integrated space-group and crystal-structure determination, *Acta Crystallographica Section C* **2015**, *71*, 3-8.

[5] O. V. Dolomanov, L. J. Bourhis, R. J. Gildea, J. A. K. Howard, H. Puschmann, OLEX2: a complete structure solution, refinement and analysis program, *Journal of Applied Crystallography* **2009**, *42*, 339-341.
